# Interactions of selected cardiovascular active natural compounds with CXCR4 and CXCR7 receptors: a molecular docking, molecular dynamics, and pharmacokinetic/toxicity prediction study

**DOI:** 10.1186/s12906-021-03488-8

**Published:** 2022-02-04

**Authors:** Hussam Aly Sayed Murad, Thamer Mohammed Ahmed Alqurashi, Mostafa Aly Hussien

**Affiliations:** 1 Department of Pharmacology, Faculty of Medicine, Rabigh, King Abdulaziz University, Jeddah, 21589 Saudi Arabia; 2 Department of Chemistry, Faculty of Science, King Abdulaziz University, Jeddah, 21589 Saudi Arabia; 3 Department of Chemistry, Faculty of Science, Port-Said University, Port-Said, 42521 Egypt

**Keywords:** ADME, Coronary artery disease, Curcumin, CXCR4, CXCR7, Docking, Eucalyptol, HOMO–LUMO, Quercetin, Resveratrol

## Abstract

**Background:**

The chemokine CXCL12 and its two receptors (CXCR4 and CXCR7) are involved in inflammation and hematopoietic cell trafficking. This study was designed to investigate molecular docking interactions of four popular cardiovascular-active natural compounds; curcumin, resveratrol, quercetin, and eucalyptol; with these receptors and to predict their drug-like properties. We hypothesize that these compounds can modify CXCL12/CXCR4/CXCR7 pathway offering benefits for coronary artery disease patients.

**Methods:**

Docking analyses were carried and characterized by Molecular Environment (MOE) software. Protein Data Bank (http://www.rcsb.org/) has been retrieved from protein structure generation and crystal structures of CXCR4 and CXCR7 receptors (PDB code = 3ODU and 6K3F). The active sites of these receptors were evaluated and extracted from full protein and molecular docking protocol was done for compounds against them. The presented parameters included docking scores, ligand binding efficiency, and hydrogen bonding. The pharmacokinetic/toxic properties (ADME/T) were calculated using SwissADME, ProTox-II, and Pred-hERG softwares to predict drug-like properties of the compounds. The thermochemical and molecular orbital analysis, and molecular dynamics simulations were also done.

**Results:**

All compounds showed efficient interactions with the CXCR4 and CXCR7 receptors. The docking scores toward proteins 3ODU of CXCR4 and 6K3F of CXCR7 were − 7.71 and − 7.17 for curcumin, − 5.97 and − 6.03 for quercetin, − 5.68 and − 5.49 for trans-resveratrol, and − 4.88 and − 4.70 for (1 s,4 s)-eucalyptol respectively indicating that all compounds, except quercetin, have more interactions with CXCR4 than with CXCR7. The structurally and functionally important residues in the interactive sites of docked CXCR4-complex and CXCR7-complex were identified. The ADME analysis showed that the compounds have drug-like properties. Only (1 s,4 s)-Eucalyptol has potential weak cardiotoxicity. The results of thermochemical and molecular orbital analysis and molecular dynamics simulation validated outcomes of molecular docking study.

**Conclusions:**

Curcumin showed the top binding interaction against active sites of CXCR4 and CXCR7 receptors, with the best safety profile, followed by quercetin, resveratrol, and eucalyptol. All compounds demonstrated drug-like properties. Eucalyptol has promising potential because it can be used by inhalation or skin massage. To our knowledge, this is the first attempt to find binding interactions of these natural agents with CXCR4 and CXCR7 receptors and to predict their druggability.

**Supplementary Information:**

The online version contains supplementary material available at 10.1186/s12906-021-03488-8.

## Background

The homeostatic chemokine stromal-cell-derived factor-1 (SDF-1) or CXCL12 and its two receptors CXCR4 and CXCR7 are considered important therapeutic targets that control inflammation. CXCR4 and CXCR7 are G protein coupled receptors (GPCR) that have similar trans-membrane helices and distinct conformation of the N- and C-terminals as well as of three extracellular loops [1, 2]. CXCL12 is abundant in many normal tissues. Blood vascular endothelial cells express both CXCL12 and its receptors [3]. The CXCL12/CXCR4/CXCR7 pathway induces migration of hematopoietic progenitor cells, endothelial cells, and leukocytes [4]. When CXCL12 interacts with CXCR4, the classical GPCR signaling occurs through phosphoinositide-3 kinase (PI3K/Akt), phospholipase C/inositol 1,4,5, triphosphate (PLC/IP3), and extracellular regulated kinase 1/2 (ERK1/2) pathways, and Ca^2+^ mobilization from endoplasmic reticulum through inhibition of adenylyl cyclase mediated c-AMP production. On the other hand, when CXCL12 interacts with CXCR7, this GPCR mobilization of Ca^2+^ does not happen, but β-arrestin is activated leading to scavenging of CXCL12 and internalization of CXCR4. Also, CXCR4 and CXCR7 can form either homodimers or heterodimers and the later induce conformational changes of the CXCR4/G proteins and block signaling [5]. Therapeutics targeting this axis can block the CXCL12/CXCR4 interaction or suppress the downstream intracellular signaling [6].

Coronary artery disease (CAD) is characterized by inadequate vasculogenic reaction to ischemia and dysfunction of endothelial outgrowth cells (EOCs). CXCR7 is a crucial modulator of neovascularization of EOCs to the perfusion defect area. In CAD patients, CXCR7 expression of EOCs was downregulated and phosphorylation of its downstream signaling (ERK) was reduced. CXCR7 upregulation increased phosphorylation of ERK and improved vasculogenic function of EOCs. Thus, CXCR7 signaling may be a new target for therapeutic vasculogenesis in CAD patients [7]. Effective improving of cardiac blood flow and controlling the atherosclerotic plaque are fundamental points in management of CAD. Angiogenesis (which is an important pathway for revascularization of ischemic tissues in CAD) improves blood flow but unfortunately, it may destabilize the atherosclerotic plaque. Thus, adjusting the balance of angiogenesis can be a main target for CAD treatment. Recently, herbal medicines are reported to be effective in adjusting such balance [8]. Poly-pharmacological compounds act on multiple targets, thus useful against complex (multifactorial) diseases. Also, through molecular docking, complexes formed between ligands and interesting targets (often many) can be observed for a particular disease [9].

Curcumin ((1E,6E)-1,7-bis (4-hydroxy- 3-methoxyphenyl) -1,6- heptadiene-3,5-dione), a polyphenol compound separated from turmeric, has antioxidant, anti-inflammatory, anticancer, anticoagulant, and lipid regulation effects. The underlying mechanisms of these effects are diverse and involve suppression of numerous molecular targets including nuclear factor-κB (NF-κB), vascular endothelial cell growth factor, pro-inflammatory cytokines, protein kinases (such as mitogen-activated protein kinases; MAPK; and Akt), enzymes (such as cyclooxygenase (COX) 1 & 2 and 5-lipoxygenase) and recently CXCR 4. Curcumin is quite safe in humans with few side effects [10]. Moreover, curcumin has displayed beneficial preventive and therapeutic effects in many neurological diseases including Alzheimer’s disease through several molecular mechanisms such as “antioxidant, amyloid β-binding, anti-inflammatory, tau inhibition, metal chelation, neurogenesis activity, and synaptogenesis promotion”. To overcome its low bioavailability, new nanocurcumin formulations with no toxicity for other body cells are being developed as therapeutic alternatives [11]. The curcumin’s anti-inflammatory effects have been linked to its phenyl and methoxy groups in the ortho position, which strongly inhibit nuclear factor kappa B and hence stop production of TNF-α and IL-6. Also, curcumin can inhibit cyclooxygenase-2, lipoxygenase, and inducible nitric oxide synthase which are key enzymes mediating inflammation. Therefore, curcumin was found to have anti-oral mucosal lesion activity due to its anti-inflammatory, antioxidant, antifungal, antiseptic, analgesic, and anti-neoplastic effects [12].

Quercetin (2-(3,4-Dihydroxyphenyl)-5,7-dihydroxy-4H-1-benzopyran-4-one) and resveratrol (5-[(E)-2-(4-Hydroxyphenyl)ethen-1-yl]benzene-1,3-diol) are natural polyphenolic compounds found in the grapes, red wine, peanuts, and other food products. Trans-resveratrol is the active form which has antioxidant, anti-inflammatory, antiplatelet, anti-carcinogenic, and DNA protective effects. It inhibits pro-inflammatory factors such as platelet-activating factor, tumor necrosis factor-α (TNF-α) and interleukin 1b (IL-1b)-induced NF-kB activation, and enzymes like COX-2. No significant side effect has been reported with it, but due to its experimental anti-platelet effect, caution should be taken with any concomitant use of anti-platelet agents [13]. Quercetin is found in all plant foods. It has antioxidant, anti-inflammatory, cardiovascular protective, anticancer, and antiviral effects. Quercetin suppressed the non-typeable *Haemophilus influenzae*-induced CXCR4 expression levels in cases of otitis media suggesting a potential therapeutic benefit [14]. Quercetin is effective as a preventive and therapeutic agent against “different neuro degenerative brain disorders such as Alzheimer’s disease, Amyloid β peptide, Parkinson’s disease, Huntington’s disease, multiple sclerosis, and amyotrophic lateral sclerosis”. It decreases stress, inflammation, and enhances brain growth. Therefore, quercetin can be considered as a powerful dietary supplement with negligible toxicity. Due to its low bioavailability, studies on drug delivery mechanisms are needed. Also, more clinical trials to assess its effective dose for treatment of the neurodegenerative disorders are recommended [15].

Aromatherapy (or essential oil therapy) is use of essential oils extracted from plants as a complementary health approach. The essential oils are most often used by inhalation or by skin application [16]. Patients with chronic diseases such as CV diseases, respiratory diseases, GI diseases, cancer, and neurological diseases can benefit from aromatherapy in addition to their routine medications to combat chronic inflammation and oxidative stress and improve side effects such as insomnia and nausea. The 1,8-cineole or (1 s,4 s)-eucalyptol (1,3,3-Trimethyl-2-oxabicyclo[2.2.2]octane), a terpenoid oxide present in several plant essential oils, has anti-inflammatory and antioxidant effects. In women with acute coronary syndrome, aromatherapy massage with lavender essential oil (in addition to the routine care) significantly decreased levels of anxiety and depression and reduced blood pressure and heart rate but did not affect the respiratory rate [17]. In coronary care units, inhalation of lavender essential oil for 2 weeks improved sleep quality and alleviated anxiety [18].

Taken together, this study was designed to investigate the molecular docking interactions of four of the most researched anti-inflammatory natural compounds (three polyphenols; curcumin, resveratrol, quercetin; and a terpene; eucalyptol) with the CXCR4 and CXCR7 receptors, and to predict their pharmacokinetic/toxic properties. The thermochemical and molecular orbital analysis and molecular dynamics simulations were done to predict their drug-like properties. We hypothesize that these compounds can modify the CXCL12/CXCR4/CXCR7 pathway commonly involved in regulation of hematopoietic cell trafficking suggesting potential additional benefits of these compounds for CAD patients.

## Methods

The manuscript’s design is summarized in this scheme followed by detailed description.﻿ A supplementary material file is provided which contains all figures presented in high quality.

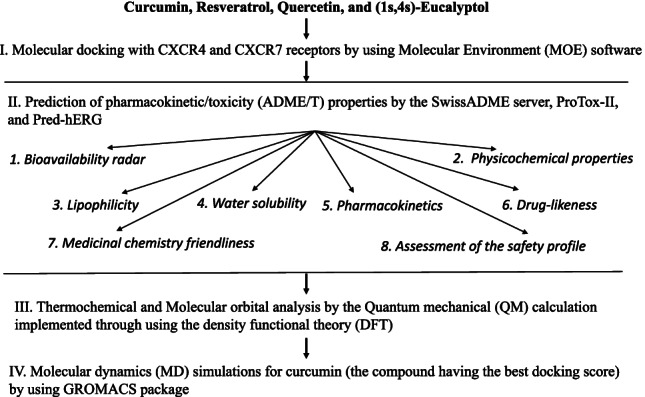



### Molecular docking analysis

The docking analyses were carried and characterized by the Molecular Environment (MOE) software. Chemdraw compound preparation, Chem 3d structures, Chem 3D ultra 12.0 (Molecular Modeling and Analysis; Cambridge Soft Corporation) software, and MOPAC were enhanced and deposited in MDL MolFile (*.mol). All calculations were performed on a (1.80-1.99) GHz-based MS Windows 10 pro 64-bit operating system Intel (R) Core (TM) i7-8550 CPU [19–22]. A Protein Data Bank (http://www.rcsb.org/) has been retrieved from protein structure generation and the crystal structures of the CXCR4 and CXCR7 receptors (PDB codes = 3ODU and 6K3F). The proteins were separated from all bound waters, ligands, and cofactors and then hydrogen atoms were applied to the optimization. Validation of the docking method was done by using the same crystalline compound “(6,6-dimethyl-5,6-dihydroimidazo(2,1-b) thiazol-3-yl)methyl (E)-N,N’-dicyclohexylcarbamimidothioate” with 3ODU protein with the same protocol and the resulting RMSD “root-mean-square deviation” value was 1.28 Å and by comparing our test compounds with the validated compounds which have a docking score of - 5.17 kcal/mol.

The molecular docking protocol was done for the four natural product compounds against the active sites of CXCR4 and CXCR7 receptors (PDB codes = 3ODU and 6K3F) [2]. The active sites were evaluated and extracted from the full protein. The energy minimization was done using MMFF94x force field [19]. London dG scoring was used as a function in the calculated docking score to rank the binding affinity of the synthesized compounds to the protein molecule [20]. The RMSD of the selected compounds position compared to the docking pose was used in the ranking.

All energy interaction and pi-pi stacking calculation were done in MOE program by using the Triangle Matcher where Poses are generated by aligning the ligand triplets of atoms on the triplets of alpha spheres in a more systematic way than in the Alpha Triangle method. The options for this method were: (1) Timeout (seconds): Amount of time allowed for each ligand placement, and (2) Number of Returned Poses: Maximum number of poses returned by each ligand placement. The scoring for high 30 pose was done using London dG Scoring function which estimates the free energy of binding of the ligand from a given pose. The functional form is a sum of terms:

$$\Delta G=c+{E}_{\mathrm{flex}}+\sum \limits_{\mathrm{h}\hbox{-} \mathrm{bonds}}{c}_{\mathrm{HB}}{f}_{\mathrm{HB}}+\sum \limits_{\mathrm{m}\hbox{-} \mathrm{lig}}{c}_{\mathrm{M}}{f}_{\mathrm{M}}+\sum \limits_{\mathrm{atoms}\kern0.5em i}\Delta {D}_i$$

where c represents the average gain/loss of rotational and translational entropy; Eflex is the energy due to loss of flexibility of the ligand (calculated from ligand topology only); fHB measures geometric imperfections of hydrogen bonds and takes a value in [0,1]; cHB is the energy of an ideal hydrogen bond; fM measures geometric imperfections of metal ligations and takes a value in [0,1]; cM is the energy of an ideal metal ligation; and Di is the desolvation energy of atom i. The difference in desolvation energies was calculated according to the formula:

$$\Delta {D}_i={c}_i{R}_i^3\left\{\underset{u\notin A\cup B}{\int \int \int }{\left|u\right|}^{-6} du-\underset{u\notin B}{\int \int \int }{\left|u\right|}^{-6} du\right\}$$

where A and B are the protein and/or ligand volumes with atom i belonging to volume B; Ri is the solvation radius of atom i (taken as the OPLS-AA van der Waals sigma parameter plus 0.5 Å); and ci is the desolvation coefficient of atom i. The coefficients {c, cHB, ci} were fitted from approximately 400 X-ray crystal structures of protein-ligand complexes with available experimental pKi data. Atoms are categorized into about a dozen atom types for the assignment of the ci coefficients. The triple integrals were approximated using Generalized Born integral formulas.

The highest best five score is calculated using GBVI/WSA dG Scoring: The GBVI/WSA ΔG is a forcefield-based scoring function which estimates the free energy of binding of the ligand from a given pose. It has been trained using the MMFF94x and AMBER99 forcefield on the 99 protein-ligand complexes of the SIE training set [Naim]. The functional form is a sum of terms:

$$\Delta G\approx c+\alpha \left[\frac{2}{3}\left(\Delta {E}_{\mathrm{Coul}}+\Delta {E}_{\mathrm{sol}}\right)+\Delta {E}_{\mathrm{vdW}}+\beta \Delta {SA}_{\mathrm{weighted}}\right]$$

where: c represents the average gain/loss of rotational and translational entropy; α, β are constants which were determined during training (along with c and are forcefield-dependent. If not using an AMBER forcefield, the parameters were set by default to the MMFF trained parameters; ECoul is the coulombic electrostatic term which is calculated using currently loaded charges, using a constant dielectric of εi = 1; Esol is the solvation electrostatic term which is calculated using the GB/VI solvation model; EvdW is the van der Waals contribution to binding; Aweighted is the surface area, weighted by exposure. This weighting scheme penalizes exposed surface area.

The hydrogen bonding pattern and hydrophobic interaction pattern between the ligand and the receptor were considered. A pair of ligand-receptor atoms of the appropriate types are considered to have a hydrogen bond interaction if they are less than 3.5 Å apart. A hydrophobic atom on the ligand is considered to have a hydrophobic interaction with a receptor residue if that atom is under 4.5 Å away from a hydrophobic atom of that residue. Poses are considered as duplicates if the same set of ligand-receptor atom pairs are involved in hydrogen bond interactions and the same set of ligand atom receptor residue pairs are involved in hydrophobic interactions.

Use the enthalpy-based Affinity dG scoring function to score generated poses. The following options may be adjusted: Hydrogen Bond: The ideal hydrogen bond affinity in energy units; Ionic Contact: The ionic Coulomb coefficient; Metal Ligation: The metal ligation affinity; Hydrophobic Contact: The hydrophobic-hydrophobic contact energy; Hydrophobic-Polar: The hydrophobic-polar contact energy; Atom-Atom: The general atom-atom contact energy.

### Prediction of pharmacokinetic/toxicity (ADME/T) properties

It was done using the SwissADME server [23, 24], ProTox-II [25], and Pred-hERG as follows [26, 27]:

#### Bioavailability radar

It is a tool for rapid appraisal of drug-likeness of a molecule. Six physicochemical properties (lipophilicity, size, polarity, solubility, flexibility, and saturation) were considered. On each axis, a physicochemical range was determined by descriptors as previously explained [28, 29] and illustrated as a pink area in which the radar plot of the compound has to fall wholly to be considered drug-like.

#### Physicochemical properties

These include simple molecular and physicochemical descriptors such as molecular weight (MW), number of specific atom types, Fraction Csp3 (carbon bond saturation as defined by fraction sp3) which measures of complexity of the molecule, number of specific bond types, and molecular refractivity (MR). Also the topological polar surface area (TPSA) was used to calculate the polar surface area (PSA) which quickly estimated some ADME properties, especially in relation to passing through the biological barriers like absorption and brain entry [29, 30].

#### Lipophilicity

It was described by calculating the partition coefficient between *n*-octanol and water (log *P*_o/w_). Five free predictors; iLOGP, XLOGP3, WLOGP, MLOGP, and SILICOS-IT are given access by SwissADME to generate the consensus log *P*_o/w_ which is the mean of the values predicted by them [31, 32].

#### Water solubility

The SwissADME uses three methods to predict water solubility (ESOL (Estimated Solubility) model [33], that of Ali et al. [34], and SILICOS-IT). The output is the Log S values which are the decimal log of the molar solubility in water. The water solubility was provided in mg/ml and mol/l along with the qualitative solubility classes.

#### Pharmacokinetics

SwissADME uses specialized models to evaluate ADME behaviors of the test compound. The first model is prediction of passive gastrointestinal absorption and blood-brain barrier (BBB) penetration [30]. The second model is prediction of being substrate or non-substrate of the permeability glycoprotein (P-gp) which is essential to evaluate active efflux through membranes e.g., from the gastrointestinal wall to the lumen or from the brain [35]. The third model is prediction of interaction of compounds with cytochrome P450 (CYP) major isoenzymes (CYP1A2, CYP2C19, CYP2C9, CYP2D6, CYP3A4) which is an important contributor in drug elimination through metabolic biotransformation. Also, inhibition of these isoenzymes is a cause of drug interactions [36] leading to toxic or other adverse effects. The fourth model is prediction of the skin permeability coefficient (*K*_p_) which is linearly correlated with molecular size and lipophilicity [37]. The more negative the log *K*_*p*_ (cm/s), the less skin permeation.

#### Drug-likeness

It is the qualitative evaluation of the opportunity for a molecule to become an oral drug with respect to oral bioavailability. It relates the physicochemical characters of a compound with its biopharmaceutical aspect in human body [38]. SwissADME uses five commonly used drug-likeness rules or filters with different ranges of properties inside of which the molecule is defined as a drug-like. These are the Lipinski [39], Ghose [40], Veber [41], Egan [42], and Muegge [43] methods. Any violation of any rule shows clearly in the output panel. Also the bioavailability score [44] is used which defines four classes of compounds with probabilities of oral bioavailability of 11, 17, 56%, or 85%.

#### Medicinal chemistry friendliness

SwissADME uses two pattern recognition filters for removal of PAINS (pan assay interference compounds) that appear as promiscuous compounds which cause potential problems in assays regardless of the protein target and can give false positive biological output [45]. In addition, “Structural Alert” is concerned with problematical functional groups which could cause toxicity, metabolic imbalance, or poor pharmacokinetics. Moreover, “leadlikeness” is similar to drug-likeness, but it focuses on physicochemical limits that define a good lead [46]. The “Synthetic accessibility” score; which ranges from 1.0 (very easy) to 10.0 (very difficult); guides selection of the most promising virtually-tested molecules to be synthetized and subjected to biological assays [47].

#### Assessment of the safety profile

The ProTox-II software predicts different toxicity endpoints such as acute toxicity, hepatotoxicity, carcinogenicity, mutagenicity, and others [25]. The Pred-hERG (human Ether-à-go-go-Related Gene) software was used to assess cardiotoxicity. It depends on statistically significant and externally predictive quantitative structure-activity relationship (QSAR) models of hERG blockage which is closely associated with severe and potentially fatal cardiac dysrhythmia. The SDF (structure data file) and SMILES (simplified molecular-input line-entry system) strings were used throughout the generation process [26, 27].

### Thermochemical and molecular orbital analysis

Quantum mechanical (QM) methods keep an important role for the calculation of thermal and molecular orbital properties. In this study, optimization of compounds was done using the density functional theory (DFT). The QM calculation was implemented through using the DFT by Gaussian 09 program package for all compounds [48]. People’s 6-31G basis set was used to optimize the compounds and other calculations for global and absolute chemical reactivity parameter were done as previously described [49, 50].

### Molecular dynamics (MD) simulations

Due to huge time consumption and limitation of our server, the 100 ns scale molecular dynamics simulation has been done for the compound having the best docking score (curcumin). The best docked complex was selected for 100 ns MD simulations using GROMACS package [51] in the explicit water conditions. The protein and ligand were separated and the topology of protein was generated using GROMOS96 53a6 force field [52]. Afterwards, the PRODRG server [53] was used for the ligand structure and the partial charges were corrected using of DFT based methods available in GAUSSIAN software suite in combination with B3LYP 6-31G (d, p) basis set and CHELPG program. Consequently, the solvation was performed using the SPC/E water model [54] and then neutralization step was proceeded by adding appropriate number of counter ions. Thereafter, the energy minimization was performed using steepest descent algorithm followed by positions restraining and then equilibration in NVT (constant volume) and NPT (constant pressure) ensemble conditions, each at 100 ps time scale. The temperature of 300 K was maintained for the system using Berendsen weak coupling method, and the pressure of 1 bar was maintained utilizing Parrinello-Rahman barostat in the equilibration stage. Furthermore, the final production stage was carried out using the LINCS algorithm and the generated trajectories were analysed for the changes in the pattern of protein-inhibitor distances, Hydrogen bonds (H-bonds), Root Mean Square Deviations (RMSD), and Radius of Gyration (Rg). The Molecular mechanics Poisson–Boltzmann surface area (MM-PBSA) protocols implemented in g_mmpbsa package [55–57] were used for the calculation of free energy of binding between the docked protein and inhibitor.

## Results

### Molecular docking

In this study, virtual screening via molecular docking was performed on four natural compounds with anti-inflammatory effects. These compounds were screened based on their interactions with the CXCR4 and CXCR7 receptor to find the most predicted compound–ligand interactions. The presented parameters include the docking scores, ligand binding efficiency and hydrogen bonding interactions. The docking scores toward the proteins 3ODU of CXCR4 and 6K3F of CXCR7 were − 7.71 and − 7.17 for curcumin, − 5.97 and − 6.03 for quercetin, − 5.68 and − 5.49 for trans-resveratrol, and − 4.88 and − 4.70 for (1 s,4 s)-eucalyptol respectively (Tables 1 and 3) indicating that all compounds, except quercetin, have more interactions with CXCR4 than with CXCR7. The curcumin docking scores were shown to be the highest compared to the other three drugs.

**Table 1:** Docking scores (kcal/mol) of curcumin, trans-resveratrol, quercetin and (1 s,4 s)-eucalyptol with protein 3ODU of CXCR4 receptor

Compound	Score	rmsd_refine	E_conf	E_place	E_score1	E_refine	E_score2
Curcumin	− 7.71	2.79	−31.72	− 59.10	−10.67	−42.34	− 7.71
Trans-resveratrol	−5.68	2.95	−44.35	−64.70	−10.42	−28.16	−5.68
Quercetin	−5.97	1.37	4.83	−70.31	−12.61	−33.13	−5.97
(1 s,4 s)-Eucalyptol	−4.88	1.26	−28.53	−37.41	−7.55	−15.62	−4.88

#### Molecular docking with CXCR4 receptor protein

The docking studies of curcumin, trans-resveratrol, quercetin and (1 s,4 s)-eucalyptol compounds have been screened to “3ODU” of CXCR4 receptor protein [2]. Curcumin was found to have the best activity compared with others in docking with 6K3F protein with scores of − 7.71, − 5.68, − 5.97, and − 4.88 respectively (Table 1).

**Fig. 1:**
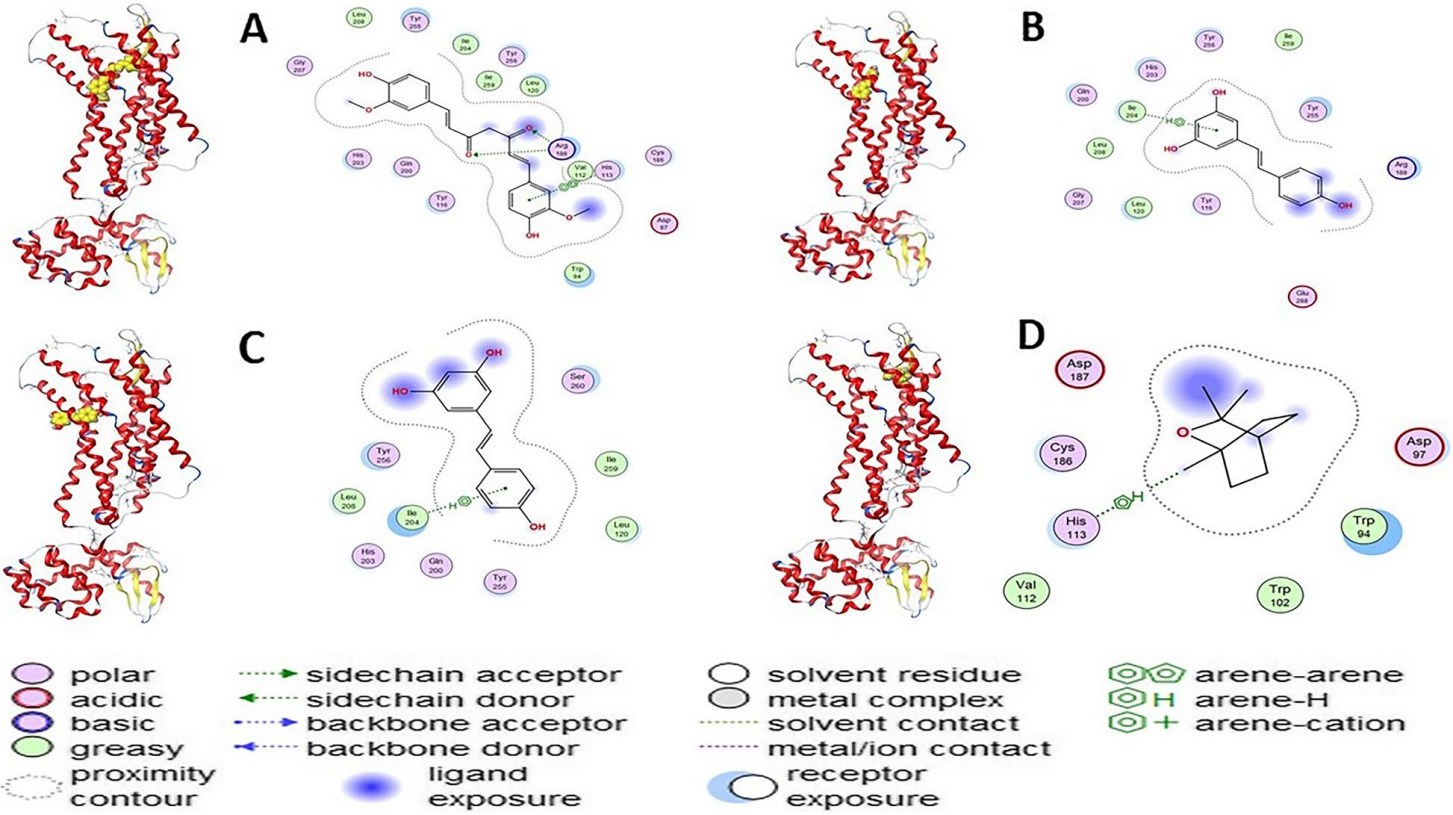
The 2D and 3D docking interaction of **A** Curcumin, **B** Trans-resveratrol, **C** Quercetin, and **D** (1 s,4 s)-Eucalyptol with protein 3ODU of CXCR4 receptor (A 3D high quality figure, S1, is presented in the supplementary material)

**Fig. 2:**
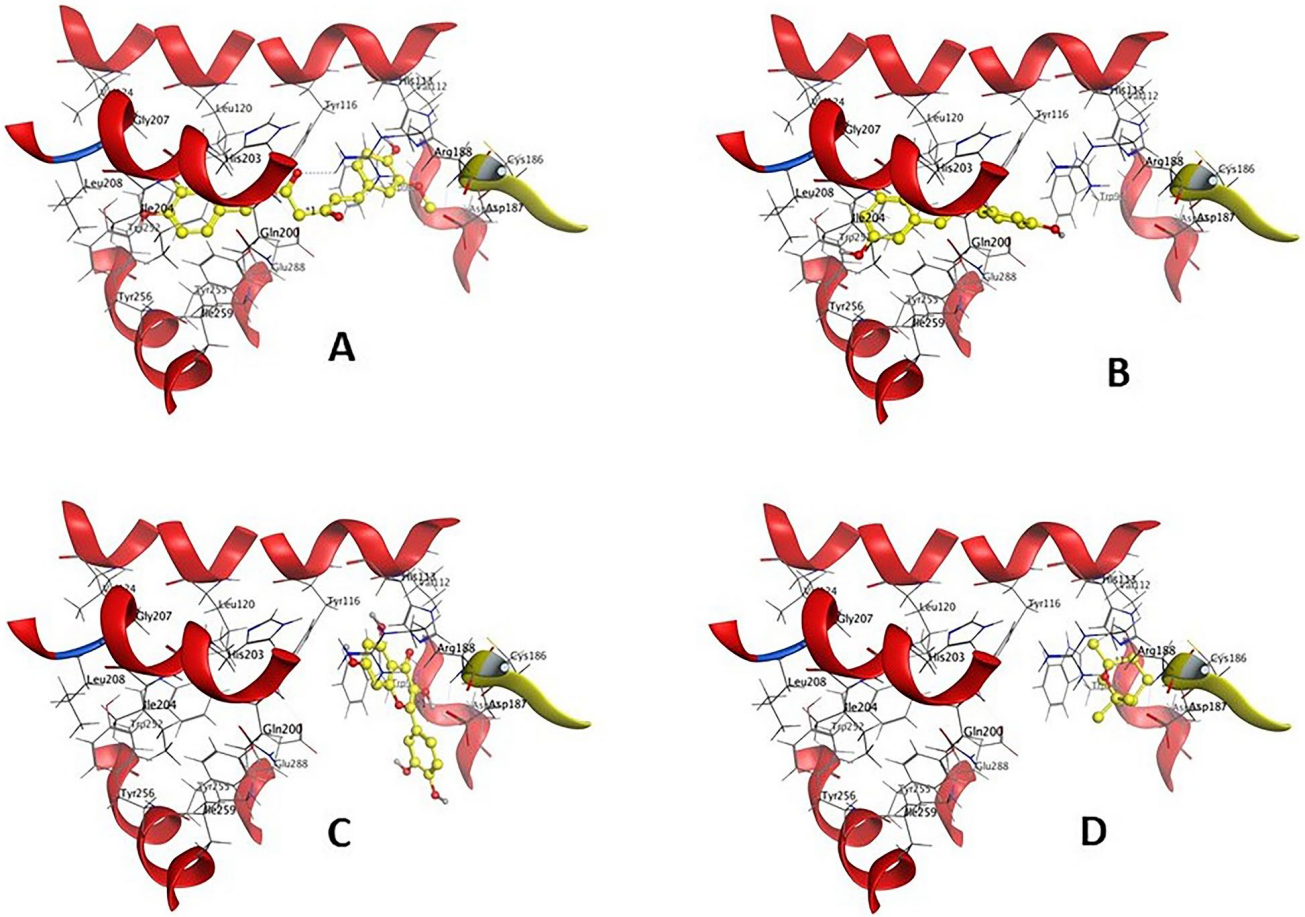
The docking sites of **A** Curcumin, **B** Trans-resveratrol, **C** Quercetin, and **D** (1 s,4 s)-Eucalyptol with protein 3ODU of CXCR4 receptor

The main structure activity relationship is contributed to the orientation of curcumin compound inside the cavity of the corresponding protein. In case of curcumin, it oriented inside the (His203-Gln200-Tyr116-Trp94-Asp97-His113-Val112-Arg188-Leu120-Lle259-Lle204-Tyr255-Leu208-Gly207) cavity due to the H-Acceptor of O1 atom of curcumin with NH(1) of Arg188 with bond length of 3.21 Å and − 2.40 Kcal/Mol, the H-Acceptor of O27 atom of curcumin with NH(2) of Arg188 with bond length of 3.13 Å and − 0.50 Kcal/Mol, and *π*- *π* stacking of 6-membered ring of curcumin with 5-membered ring of His113 with bond length of 3.88 Å and − 0.00 Kcal/Mol. In case of trans-resveratrol, it oriented inside the cavity of (His203-Lle204-Leu208-Gly207-Leu120-Tyr116-Glu288-Arg188-Tyr255-Lle259-Tyr256) by the H- *π* stacking interaction of 6-membered ring of trans-resveratrol with C atom of Ile204 with bond length of 4.33 Å and − 0.80 Kcal/Mol. In case of quercetin the compound oriented inside the cavity of (Tyr256-Lle204-Gin200-Tyr255-Leu120-Lle259-Ser260) by the *π*-H stacking interaction of 6-membered ring of quercetin with C atom of Ile204 with bond length of 4.39 Å and − 0.80 Kcal/Mol, but in case of (1 s,4 s)-eucalyptol the compound oriented inside the cavity of (Asp187-Cys186-His113-Trp102-Asp97) by the H- *π* stacking interaction between 5 membered ring of (1 s,4 s)-eucalyptol with His113 with bond length of 4.08 Å and − 0.70 kcal/mol (Figs. 1 and 2) and (Table 2).

**Table 2:** Docking interaction of curcumin, trans-resveratrol, quercetin and (1 s,4 s)-eucalyptol with protein 3ODU of CXCR4 receptor

Compound	Ligand	Receptor	Interaction	Distance (Å)	E (Kcal/Mol)
Curcumin	O 1	NH1 ARG 188 (A)	H-acceptor	3.21	−2.40
	O 27	NH2 ARG 188 (A)	H-acceptor	3.13	−0.50
	6-ring	5-ring HIS 113 (A)	pi-pi	3.88	0.00
Trans-resveratrol	6-ring	CA ILE 204 (A)	pi-H	4.33	−0.80
Quercetin	6-ring	CA ILE 204 (A)	pi-H	4.39	−0.80
(1 s,4 s)-eucalyptol	5-ring	HIS 113 (A)	H-pi	4.08	−0.70

#### Molecular docking with CXCR7 receptor protein

The docking studies of curcumin, trans-resveratrol, quercetin and (1 s,4 s)-eucalyptol compounds have been screened to “6K3F” of CXCR7 receptor protein [58]. Curcumin was found to have the highest activity compared with others in docking with 3ODU protein with scores of − 7.17, − 5.49, and − 6.03 and − 4.70 respectively (Table 3).

**Table 3:** Docking scores (kcal/mol) of curcumin, trans-resveratrol, quercetin and (1 s,4 s)-eucalyptol with protein 6K3F of CXCR 7 receptor

Compound	Score	rmsd_refine	E_conf	E_place	E_score1	E_refine	E_score2
Curcumin	−7.17	1.05	−33.09	− 81.95	−10.17	− 33.42	− 7.17
Trans-resveratrol	−5.49	1.71	−46.85	−67.60	−9.93	−27.15	− 5.49
Quercetin	−6.03	1.20	5.11	−102.43	−11.59	−36.25	−6.03
(1 s,4 s)-Eucalyptol	−4.70	2.15	−29.44	−29.89	−6.55	− 20.54	−4.70

**Fig. 3:**
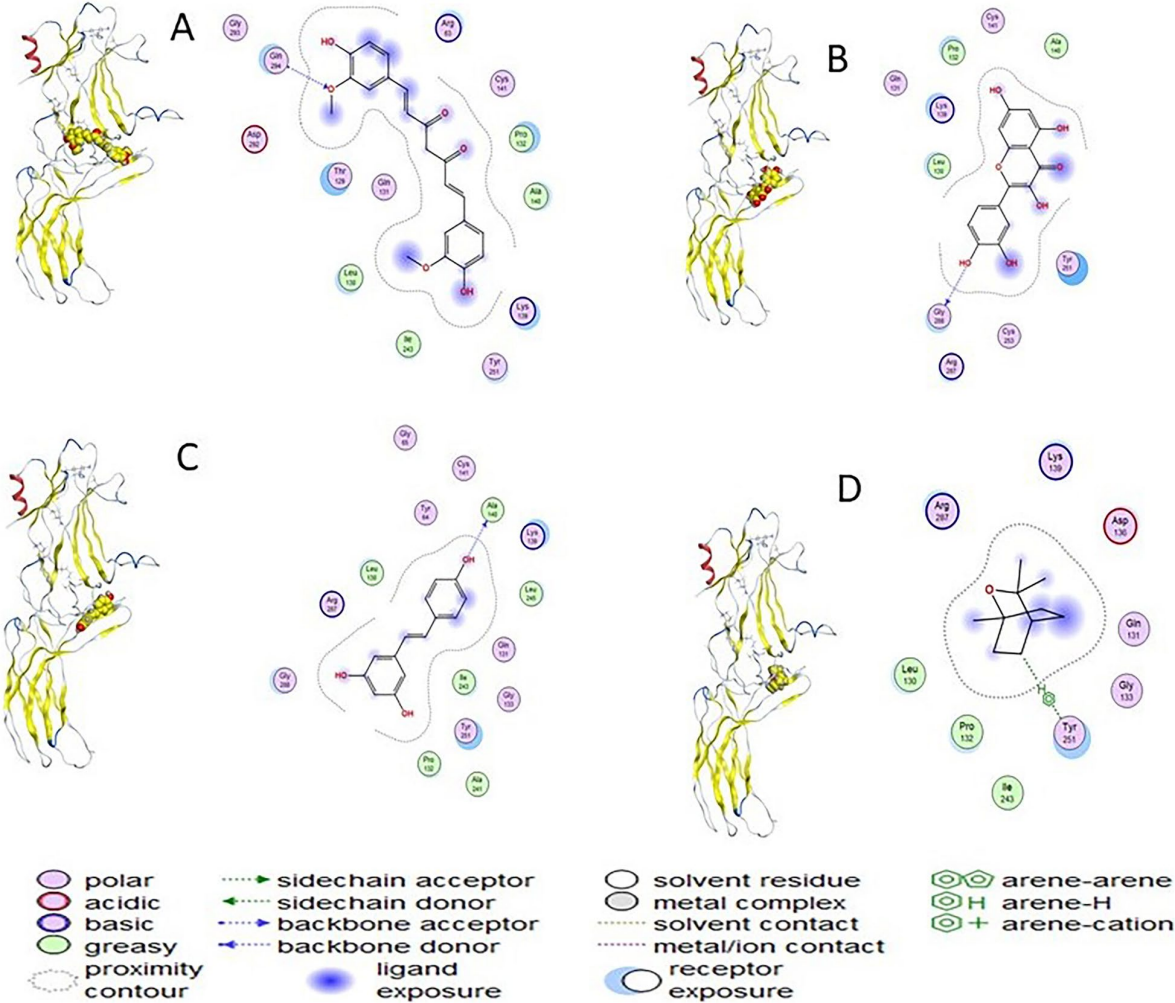
The 2D and 3D docking interaction of **A** Curcumin, **B** Trans-resveratrol, **C** Quercetin, and **D** (1 s,4 s)-Eucalyptol with protein 6K3F of CXCR7 receptor (A 3D high quality figure, S3﻿, is presented in the supplementary material)

**Fig. 4:**
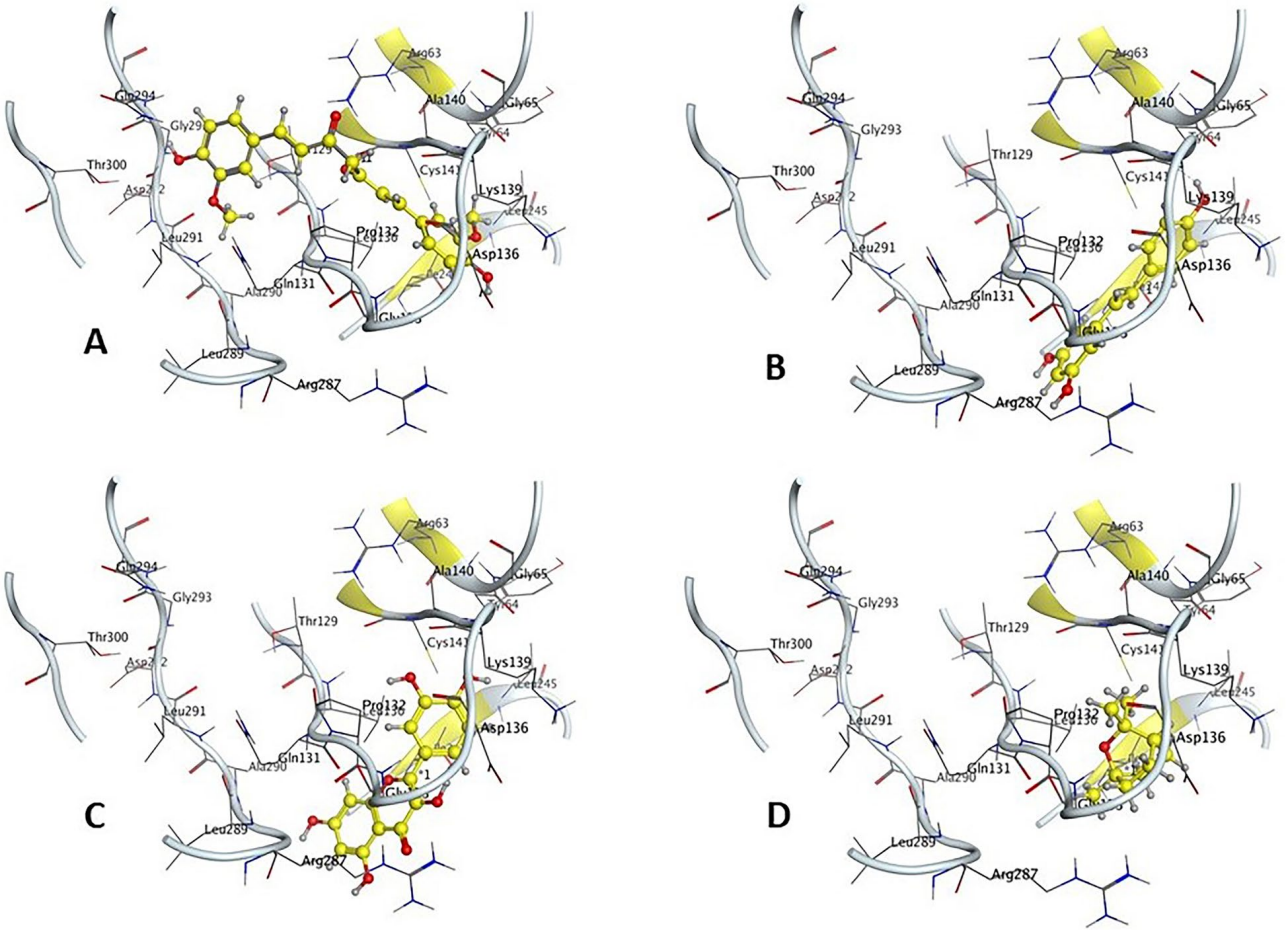
The docking sites of **A** Curcumin, **B** Trans-resveratrol, **C** Quercetin, and **D** (1 s,4 s)-Eucalyptol with protein 6K3F of CXCR7 receptor

The main structure activity relationship is contributed to the orientation of the curcumin inside the cavity of the corresponding protein. In case of curcumin, the compound oriented inside the (Gln294-Asp292-Thr129-Gln131-Leu130-Lle243-Thr251-Lys139-Ala140-Pro132-Cys141-Arg63) cavity due to the H-Acceptor of O16 atom of curcumin with N atom of Gln294 with bond length of 3.28 Å and − 0.60 Kcal/Mol. In case of trans-resveratrol, the compound oriented inside the cavity of (Gly286-Arg287-Cys253-Tyr251-Ala140-Cys141-Pro132-Lys132-Leu130) by the H-Donor interaction of O28 atom of trans-resveratrol with O atom of Ala140 with bond length of 2.99 Å and − 1.40 Kcal/Mol. Moreover, in case of quercetin the compound oriented inside the cavity of (Gly288-Arg287-Cys253-Tyr251-Ala140-Cys141-Pro132-Lys139-Leu130) by the H-Donor interaction of O29 atom of quercetin with O atom of Gly288 with bond length of 3.29 Å and − 0.50 Kcal/Mol, but in case of (1 s,4 s)-eucalyptol the compound oriented inside the cavity of (Leu130-Pro132-Lie243-Tyr251-Gly133-Gln131-Asp136-Lys139-Arg287) by the H- *π* stacking interaction between C6 atom of (1 s,4 s)-eucalyptol with 6-membered ring of Tyr251 with bond length of 4.29 Å and − 0.60 kcal/mol (Figs. 3 and 4) and (Table 4).

**Table 4:** Docking interaction of curcumin, trans-resveratrol, quercetin and (1 s,4 s)-eucalyptol with protein 6K3F of CXCR7 receptor

Compound	Ligand	Receptor	Interaction	Distance (Å)	E (Kcal/Mol)
Curcumin	O 16	N GLN 294 (A)	H-acceptor	3.28	− 0.60
Trans-resveratrol	O 28	O ALA 140 (A)	H-donor	2.99	−1.40
Quercetin	O 29	O GLY 288 (A)	H-donor	3.29	−0.50
(1 s,4 s)-eucalyptol	C 6	6-ring TYR 251 (A)	H-pi	4.29	−0.60

#### Conformation of ligands at the inhibition binding site of receptor protein

**Fig. 5:**
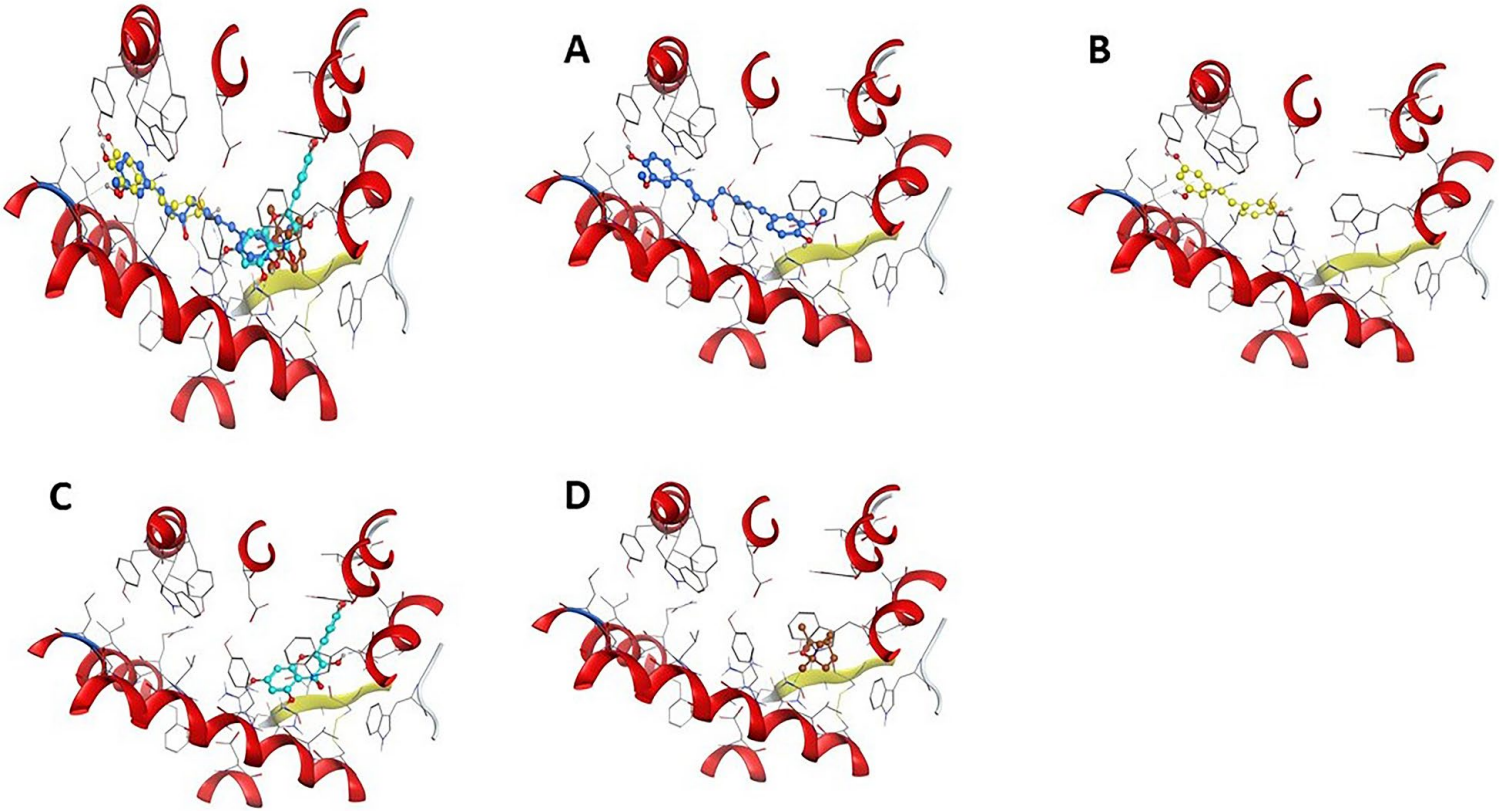
The docked conformation of **A** Curcumin, **B** Trans-resveratrol, **C** Quercetin, and **D** (1 s,4 s)-Eucalyptol at the inhibition binding site of receptor protein and superimposed structures of docked ligands with protein 3ODU of CXCR4 receptor

**Fig. 6:**
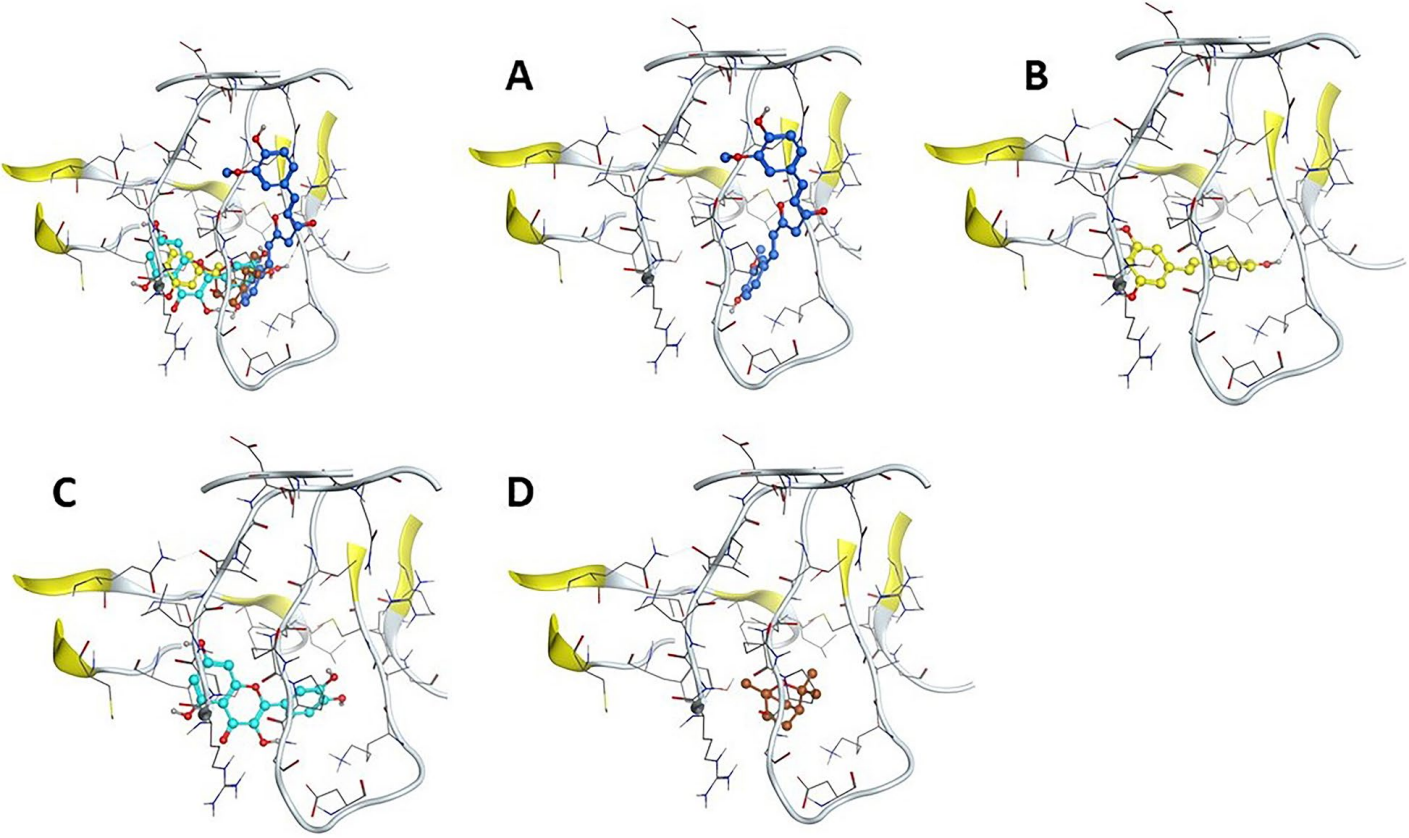
The docked conformation of **A** Curcumin, **B** Trans-resveratrol, **C** Quercetin, and **D** (1 s,4 s)-Eucalyptol at the inhibition binding site of receptor protein and superimposed structures of docked ligands with protein 6K3F of CXCR 7 receptor

The conformation of ligands at the inhibition binding site of receptor protein and superimposed structures of docked ligands are shown in Figs. 5 and 6.

### Pharmacokinetic/toxic (ADME/T) properties

#### Bioavailability radar

**Fig. 7:**
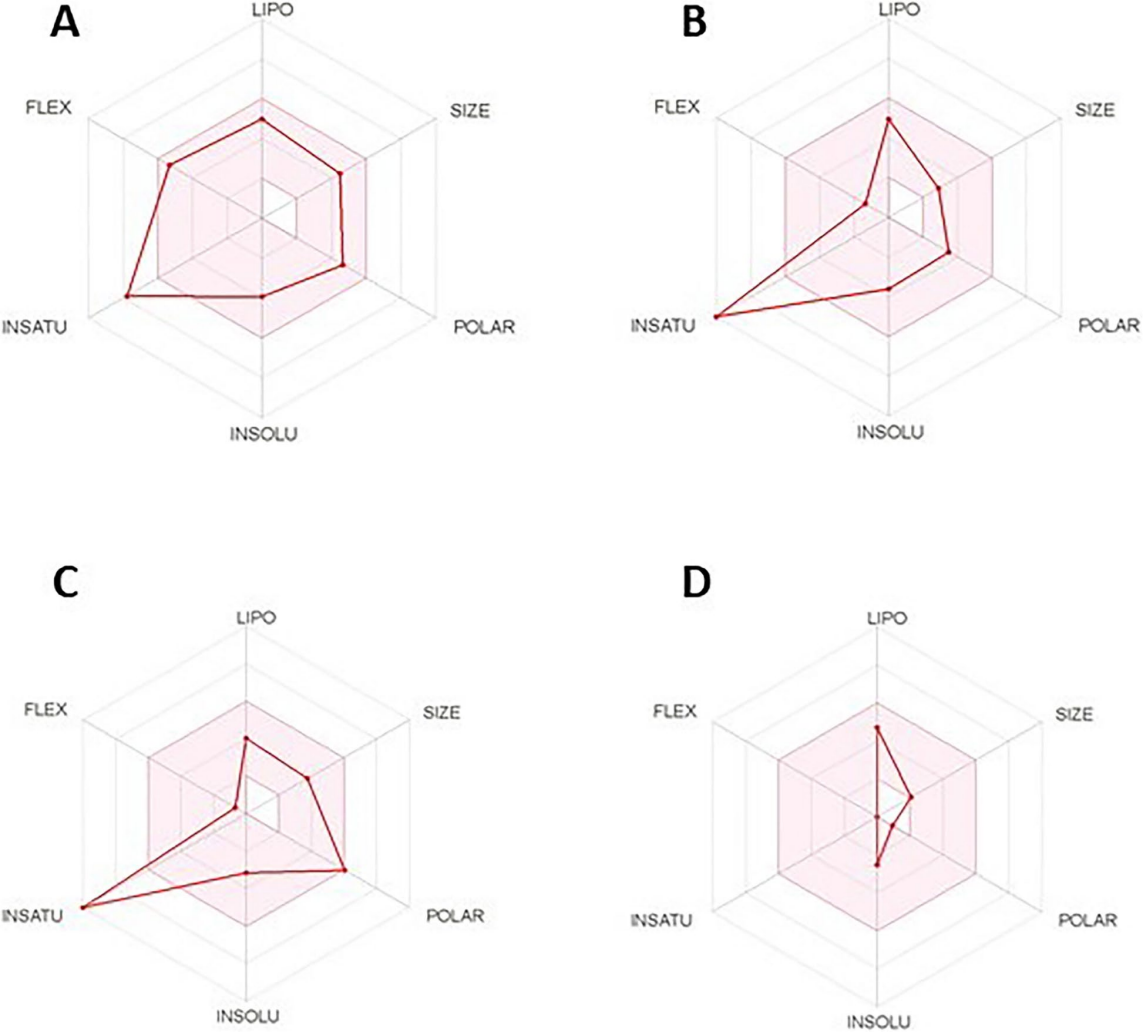
The bioavailability radar with the pink area. It represents the optimal range for physicochemical properties for oral bioavailability. Lipophilicity (− 0.7 < XlogP3 < + 0.5), size (150 < MW < 500 g/mol), polarity (20A^2^ <TPSA <130A^2^), Insolubility (0 < Log S (ESOL) < 6), Insaturation (0.25 < Fraction Csp^3^ < 1), and flexibility (number of rotatable bonds < 9). The compounds, **A** Curcumin, **B** Trans-resveratrol, **C** Quercetin, and **D** (1 s,4 s)-Eucalyptol, are predicted orally bioavailable

The four compounds are predicted orally bioavailable (Fig. 7).

#### Physicochemical properties

Simple molecular and physicochemical properties of the four compounds are shown in Table 5.

**Table 5:** Molecular and physicochemical descriptors of curcumin, trans-resveratrol, quercetin and (1 s,4 s)-eucalyptol

	Curcumin	﻿Trans-resveratrol	Quercetin	(1 s,4 s)-Eucalyptol
Formula	C21H20O6	C14H12O3	C15H10O7	C10H18O
Molecular weight (MW)	368.38 g/mol	228.24 g/mol	302.24 g/mol	154.25 g/mol
Number of heavy atoms	27	17	22	11
Number of aromatic heavy atoms	12	12	16	0
Carbon bond saturation (Fraction Csp3)	0.14	0.00	0.00	1.00
Number of rotatable bonds	8	2	1	0
Number of H-bond acceptors	6	3	7	1
Number of H-bond donors	2	3	5	0
Molar Refractivity (MR)	102.80	67.88	78.04	47.12
Topological polar surface area (TPSA)	93.06 Å^2^	60.69 Å^2^	131.36 Å^2^	9.23 Å^2^

#### Lipophilicity

The values of the log *P*_o/w_ of the five predictors of lipophilicity and the consensus (log *P*_o/w_) for curcumin, trans-resveratrol, quercetin and (1 s,4 s)-eucalyptol are shown in Table 6.

**Table 6:** Values of the partition coefficient between *n*-octanol and water (log *P*_o/w_) of the five predictors of lipophilicity and the consensus (log *P*_o/w_) of curcumin, trans-resveratrol, quercetin and (1 s,4 s)-eucalyptol

	Curcumin	Trans-resveratrol	Quercetin	(1 s,4 s)-Eucalyptol
Log *P*_o/w_ (iLOGP)	3.27	1.71	1.63	2.58
Log *P*_o/w_ (XLOGP3)	3.20	3.13	1.54	2.74
Log *P*_o/w_ (WLOGP)	3.15	2.76	1.99	2.74
Log *P*_o/w_ (MLOGP)	1.47	2.26	−0.56	2.45
Log *P*_o/w_ (SILICOS-IT)	4.04	2.57	1.54	2.86
Consensus Log *P*_o/w_	3.03	2.48	1.23	2.67

#### Water solubility

The predicted values are the decimal log of the molar solubility in water (log *S*) for the three methods which predict water solubility of the four compounds (ESOL, Ali, and SILICOS-IT) with qualitative solubility classes (Table 7).

**Table 7:** Water Solubility (Log S) values of the three predictors for curcumin, trans-resveratrol, quercetin and (1 s,4 s)-eucalyptol. The water solubility is provided in mg/ml and mol/l along with qualitative solubility classes

	Curcumin	Trans-resveratrol	Quercetin	(1 s,4 s)-Eucalyptol
Log *S* (ESOL)	−3.94	−3.62	− 3.16	− 2.52
Solubility	4.22e-02 mg/ml 1.15e-04 mol/l	5.51e-02 mg/ml 2.41e-04 mol/l	2.11e-01 mg/ml 6.98e-04 mol/l	4.63e-01 mg/ml 3.00e-03 mol/l
Class	Soluble	Soluble	Soluble	Soluble
Log *S* (Ali)	−4.83	− 4.07	−3.91	− 2.59
Solubility	5.50e-03 mg/ml 1.49e-05 mol/l	1.93e-02 mg/ml 8.44e-05 mol/l	3.74e-02 mg/ml 1.24e-04 mol/l	3.98e-01 mg/ml 2.58e-03 mol/l
Class	Moderately soluble	Moderately soluble	Soluble	Soluble
Log *S* (SILICOS-IT)	−4.45	−3.29	− 3.24	− 2.45
Solubility	1.31e-02 mg/ml 3.56e-05 mol/l	1.18e-01 mg/ml 5.16e-04 mol/l	1.73e-01 mg/ml 5.73e-04 mol/l	5.45e-01 mg/ml 3.53e-03 mol/l
Class	Moderately soluble	Soluble	Soluble	Soluble

#### Pharmacokinetics

Prediction of the gastrointestinal absorption, blood-brain barrier (BBB) penetration, effect on permeability glycoprotein (P-gp) and cytochromes P450 (CYP) major isoenzymes and skin permeation (log *K*_*p*_) for curcumin, trans-resveratrol, quercetin and (1 s,4 s)-eucalyptol are shown in Table 8.

**Table 8:** Prediction of the main pharmacokinetics parameters for curcumin, trans-resveratrol, quercetin and (1 s,4 s)-eucalyptol. These include gastrointestinal absorption, blood-brain barrier (BBB) penetration, effect on permeability glycoprotein (P-gp) and cytochromes P450 (CYP) major isoenzymes and skin permeation (log *K*_*p*_)

	Curcumin	Trans-resveratrol	Quercetin	(1 s,4 s)-Eucalyptol
GI absorption	High	High	High	High
Blood-brain barrier (BBB) permeant	No	Yes	No	Yes
P-gp substrate	No	No	No	No
CYP1A2 inhibitor	No	Yes	Yes	No
CYP2C19 inhibitor	No	No	No	No
CYP2C9 inhibitor	Yes	Yes	No	No
CYP2D6 inhibitor	No	No	Yes	No
CYP3A4 inhibitor	Yes	Yes	Yes	No
Log *K*_p_ (skin permeation)	−6.28 cm/s	−5.47 cm/s	−7.05 cm/s	− 5.30 cm/s

#### Drug-likeness

Only (1 s,4 s)-Eucalyptol showed a violation of Ghose rule and two violations of Muegge rule. The four compounds reached a bioavailability score value of 0.55 (Table 9).

**Table 9:** Prediction of the drug likeness for curcumin, trans-resveratrol, quercetin and (1 s,4 s)-eucalyptol. Five rule-based filters and the bioavailability score were used

	Curcumin	Trans-resveratrol	Quercetin	(1 s,4 s)-Eucalyptol
Lipinski rule	Yes; 0 violation	Yes; 0 violation	Yes; 0 violation	Yes; 0 violation
Ghose rule	Yes	Yes	Yes	No; 1 violation: MW < 160
Veber rule	Yes	Yes	Yes	Yes
Egan rule	Yes	Yes	Yes	Yes
Muegge rule	Yes	Yes	Yes	No; 2 violations: MW < 200, Heteroatoms< 2
Bioavailability Score	0.55	0.55	0.55	0.55

#### Medicinal chemistry friendliness

Regarding PAINS filter, only quercetin maintains undesirable moiety while regarding Brenk filter, all compounds, except (1 s,4 s)-Eucalyptol, maintain undesirable moieties. Regarding leadlikeness, all compounds, except quercetin, show violations (Table 10).

**Table 10:** The medicinal chemistry friendliness filters for curcumin, trans-resveratrol, quercetin and (1 s,4 s)-eucalyptol

	Curcumin	Trans-resveratrol	Quercetin	(1 s,4 s)-Eucalyptol
PAINS	0 alert	0 alert	1 alert: catechol	0 alert
Brenk	2 alerts: beta_keto_anhydride, michael_acceptor_1	1 alert: stilbene	1 alert: catechol	0 alert
Leadlikeness	No; 2 violations: MW > 350, Rotors> 7	No; 1 violation: MW < 250	Yes	No; 1 violation: MW < 250
Synthetic accessibility	2.97	2.02	3.23	3.65

#### Safety profile

**Fig. 8:**
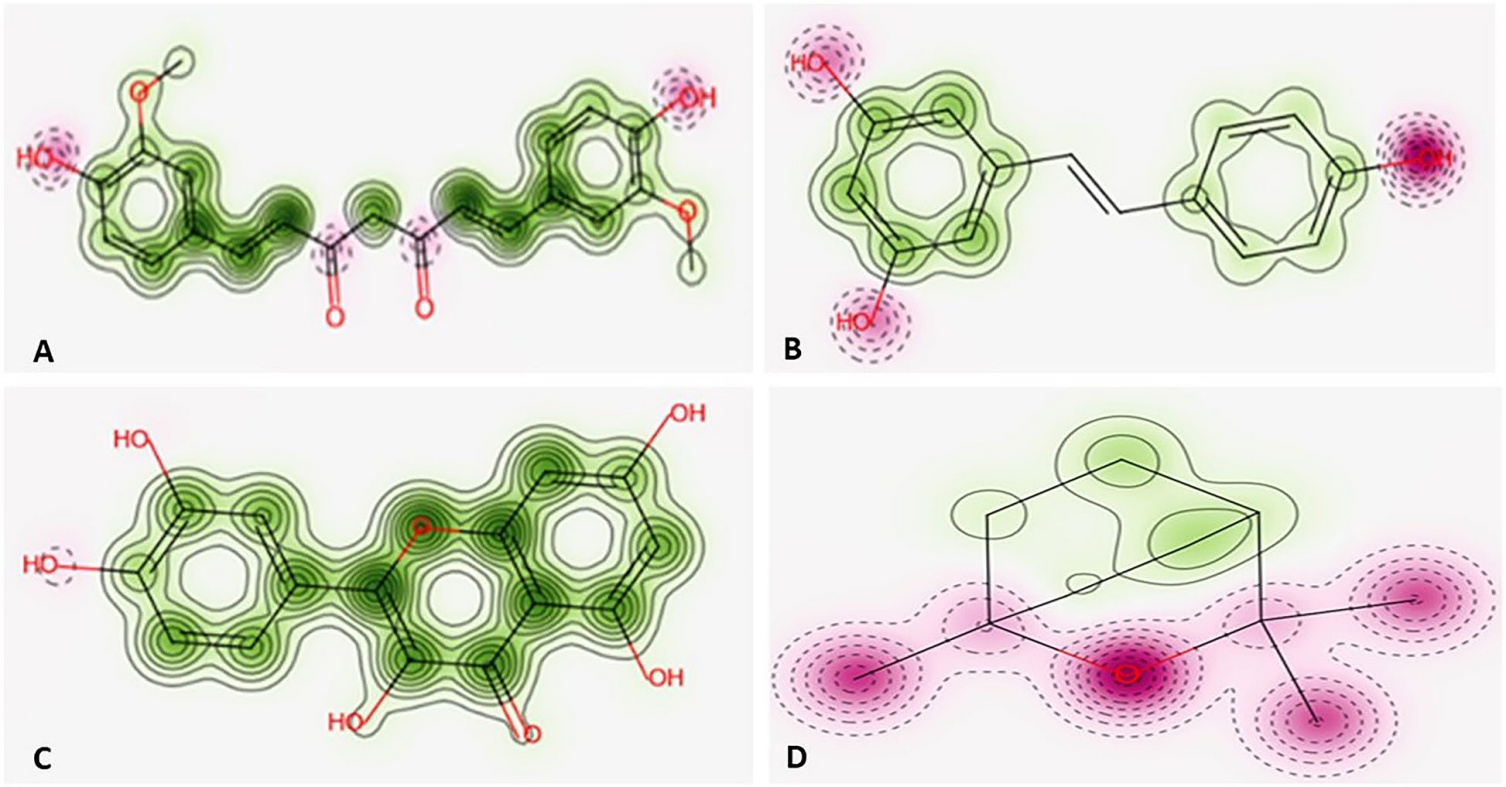
The predicted probability maps of **A** Curcumin, **B** Trans-resveratrol, **C** Quercetin, and **D** (1 s,4 s)-Eucalyptol. The more contour lines and intense green color means a higher positive contribution of an atom or a fragment to the hERG blockage, while pink means that it contributes to decrease of hERG blockage, and gray means no contribution

The ProTox-II showed that the four compounds are predicted to have oral LD50 value ranging from 159 to 2480 mg/kg in a rat model with (1 s,4 s)-Eucalyptol bearing the highest values and quercetin bearing the lowest one. The Pred-hERG showed that curcumin, trans-resveratrol, and quercetin are non-cardiotoxic while (1 s,4 s)-Eucalyptol may have cardiotoxicity. In addition, only curcumin has a good applicability domain (AD) (Table 11). The predicted probability maps are shown in Fig. 8.

**Table 11:** The predicted toxicity for curcumin, trans-resveratrol, quercetin and (1 s,4 s)-eucalyptol using: (1) ProTox-II and (2) Pred-hERG softwares

	Curcumin	Trans-resveratrol	Quercetin	(1 s,4 s)-Eucalyptol
**(1) ProTox-II**				
Predicted LD50 (mg/kg)	2000	1560	159	2480
Predicted toxicity class	4	4	3	5
Average similarity (%)	100	69.97	100	100
Prediction accuracy (%)	100	68.07	100	100
**(2) Pred-hERG**				
Prediction/Potency	Non-cardiotoxic	Non-cardiotoxic	Non-cardiotoxic	Potential cardiotoxic (Weak or moderate)
Confidence (%)	70	60	60	80
Applicability domain (AD)	Yes (Value = 0.28 and limit = 0.26)	No (Value = 0.21 and limit = 0.26)	No (Value = 0.19 and limit = 0.26)	No (Value = 0.16 and limit = 0.26)

### Thermochemical and molecular orbital results

**Fig. 9:**
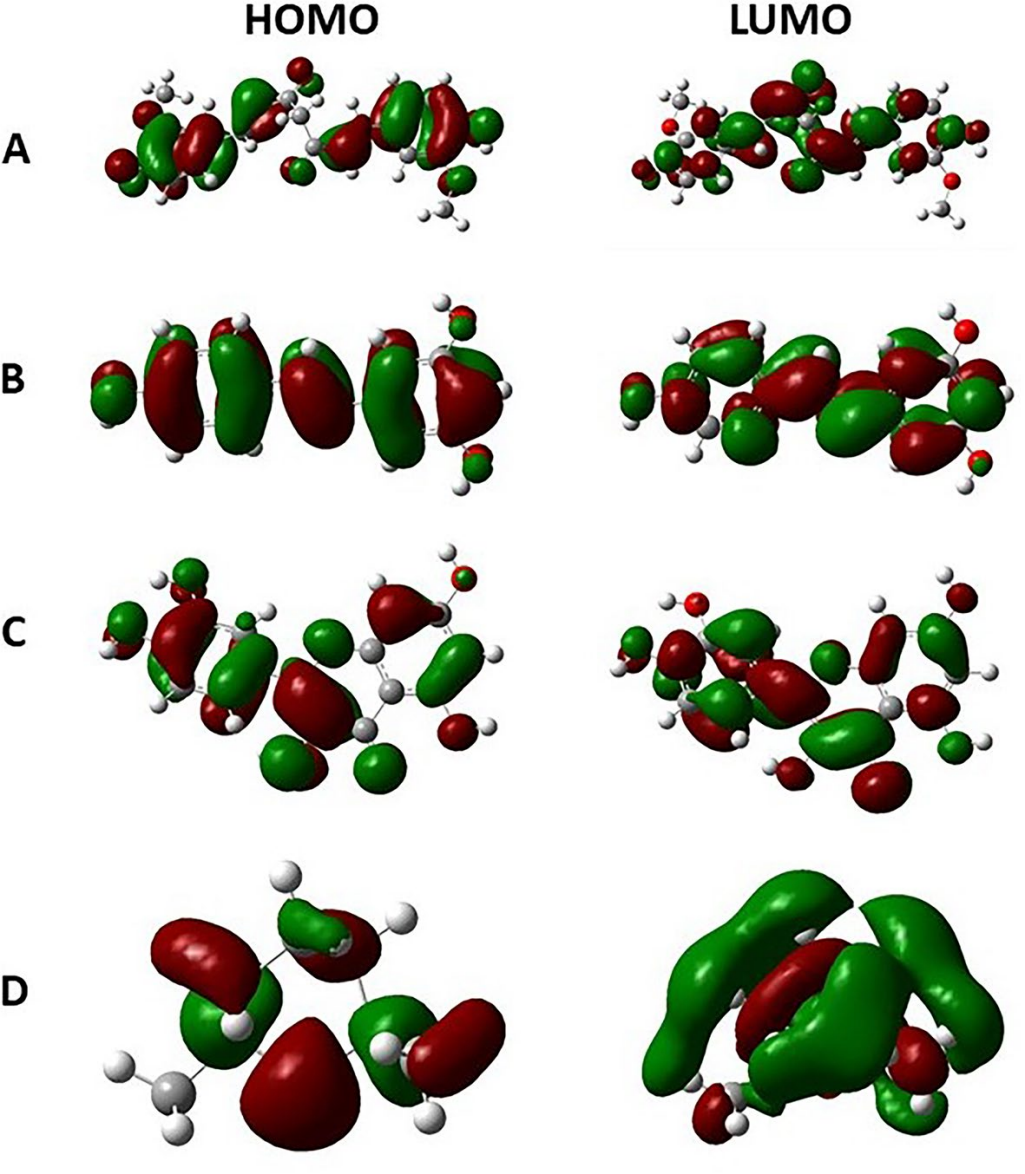
The 3D HOMO and LUMO frontier molecular orbital of **A** Curcumin, **B** Trans-resveratrol, **C** Quercetin, and **D** (1 s,4 s)-Eucalyptol

The HOMO (highest occupied molecular orbital) and LUMO (lowest unoccupied molecular orbital) energies, gap, absolute electronegativities, absolute and global hardness, absolute and global softness, chemical potentials, global electrophilicity, and additional electronic charge of all compounds are presented in Tables 12 and 13 and Fig. 9.

**Table 12:** Energy (eV) of HOMO, LUMO, and global chemical reactivity parameters of curcumin, trans-resveratrol, quercetin, and (1 s,4 s)-eucalyptol

Compound	HOMO	LUMO	∆ E	I	A	χ	ɳ	σ	Pi	S	H
Curcumin	− 157.034	−30.183	126.851	157.034	30.183	93.609	63.426	0.016	−93.609	0.008	126.851
Trans-resveratrol	− 124.950	−32.022	92.928	124.950	32.022	78.486	46.464	0.022	− 78.486	0.011	92.928
Quercetin	−132.436	−36.709	95.727	132.436	36.709	84.573	47.863	0.021	− 84.573	0.010	95.727
(1 s,4 s)-Eucalyptol	− 142.821	−41.780	101.042	142.821	41.780	92.300	50.521	0.020	−92.300	0.010	101.042

*HOMO* Highest occupied molecular orbital (EHOMO), *LUMO* Lowest unoccupied molecular orbital, *∆ E* HOMO–LUMO energy gap, *I* Ionization potential, *A* Electron affinity, *χ* Absolute electronegativities, *ɳ* Absolute hardness, *σ* Absolute softness, *Pi* Chemical potentials, *S* Global softness, *H* Global hardness

**Table 13:** Enthalpies and free energies of curcumin, trans-resveratrol, quercetin and (1 s,4 s)-eucalyptol

	Hartree × 10^3^	Kcal/mol × 10^5^	Hartree × 10^2^	Kcal/mol × 10^5^	Hartree × 10^3^	Kcal/mol ×10^5^	Hartree ×10^2^	Kcal/mol × 10^5^
Compound	Curcumin		Trans-resveratrol		Quercetin		(1 s,4 s)-Eucalyptol	
Enthalpies (ΔH)	−1.26	− 7.92	− 7.66	−4.81	−1.10	−6.92	−4.67	−2.93
Free Energies (ΔG)	−1.26	− 7.92	− 7.66	− 4.81	−1.10	−6.93	−4.67	− 2.93

### Molecular dynamics (MD) simulations

**Fig. 10:**
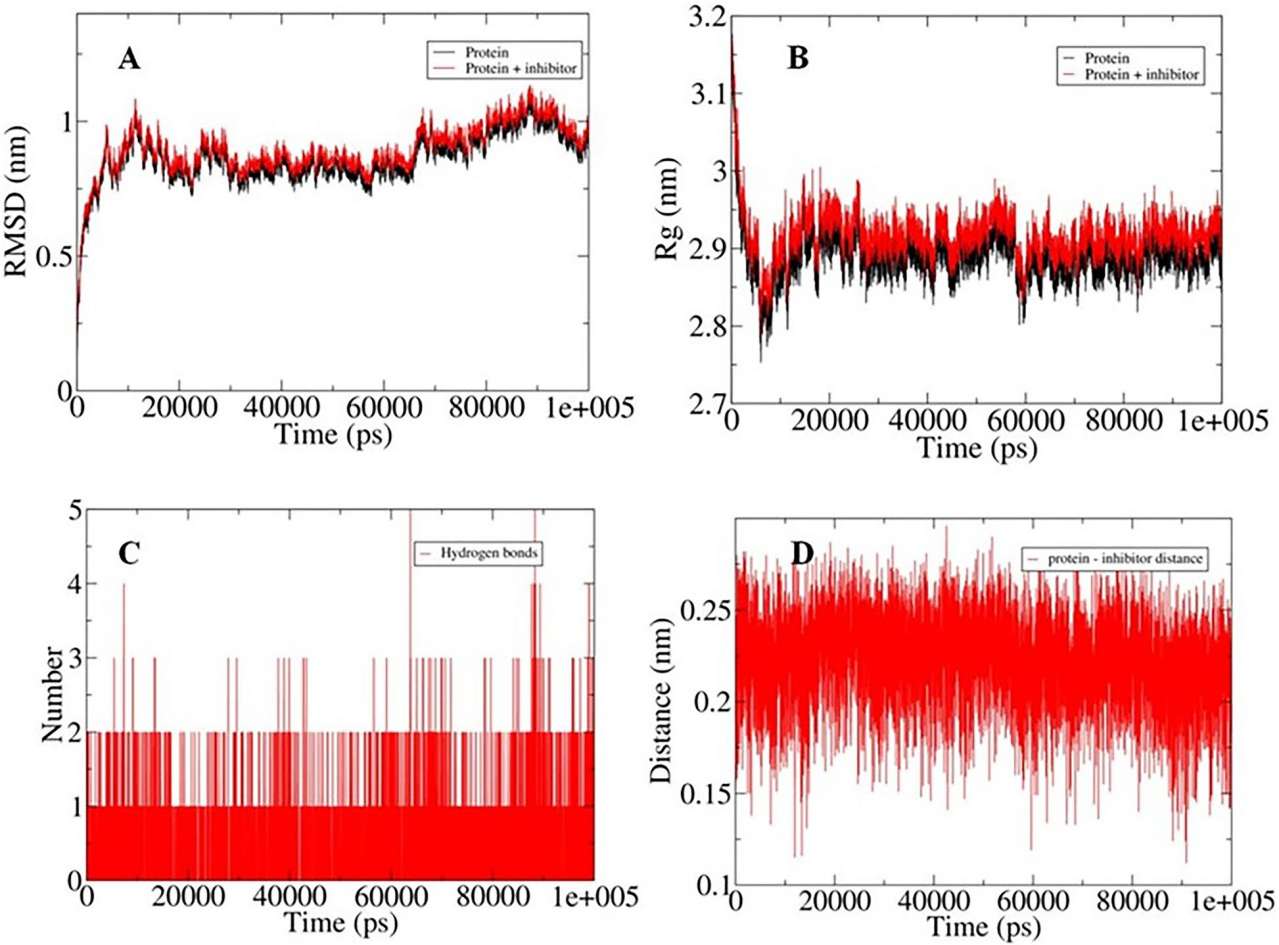
The results obtained from 100 ns molecular dynamics (MD) simulations with: **A** Changes in the root mean square deviation (RMSD) values for free protein and docked system, **B** Changes in radius of gyration (Rg), **C** Changes in the pattern of hydrogen binding, **D** Calculated distance between the protein and inhibitor

The protein complex with the highest binding affinities was selected for MD simulations, with the RMSD values provided the insight into the stability profile with the values for both the protein and docked system was fluctuating between 0.5 nm and 1.0 nm (Fig. 10A). There is a little perturbation in the RMSD values indicating the stability achieved by the binding of inhibitor in the docked complex. Furthermore, the compactness of the studied systems was analyzed in terms of Rg values which showed variation between 2.8 nm and 3.0 nm indicating the stability achieved in the system after 20 ns time period as indicated from the lower fluctuations in the graph (Fig. 10B). On the basis of the hydrogen bonds and calculated protein-inhibitor distances, it can be observed that the protein and ligand achieved a high degree of interactions (Fig. 10C & D). The docked complex showed up to five hydrogen bonds and the distance between the two molecules fluctuated between 0.15 nm and 0.25 nm.

## Discussion

Molecular docking is an efficient tool widely used to know the molecular characteristics of protein-ligand interactions [59]. The SwissADME server is used to predict the pharmacokinetics of compounds and their probability to be drug candidates [23]. In addition to their known anti-inflammatory effects, our study shows that the three polyphenols; curcumin, resveratrol, quercetin; and the terpene eucalyptol interact efficiently with the CXCR4 and CXCR7 receptor, revealing different therapeutic modes of action through potentially regulating theses receptors. All compounds, except quercetin, have more interactions with CXCR4 than with CXCR7. Curcumin showed the highest docking scores followed by quercetin, resveratrol, and (1 s,4 s)-Eucalyptol. The study of docking to CXCR4 and CXCR7 has opened a novel aspect in this regard and its results should be validated through experimental studies. The structurally and functionally important residues such as inhibitor-binding residues in the interactive site of docked CXCR4-complex and CXCR7-complex were identified. Recently, it was reported that CXCR7 activation may be a possible therapeutic target in cases of ischemic myocardium. It protected ischemic cells in hypoxic endothelial cells and infarction model by stimulating angiogenesis and decreasing apoptosis in C57BL/6 J mice model of acute myocardial infarction [60].

The structure of curcumin makes it an interesting lead for more development of novel antagonist candidates. Its established efficacy, proven safety, and reduced cost make is a promising agent for prevention and treatment of various human diseases. However, clinical use of curcumin is limited by its low bioavailability and many studied tried to reformulate it to enhance its bioavailability, but full evaluation of efficacy and safety of reformulated curcumin is still lacking [61]. In rat coronary atherosclerosis heart disease model, the permeability of coronary artery was found to be high which may be attributed to up-regulation of matrix metalloproteinase-9 (MMP-9), TNF-α and C-reactive protein (CRP) expression. Administration of oral curcumin (100 mg/kg/d) for 4 weeks inhibited expression of MMP-9, TNF-α and CRP and improved the permeability of coronary artery [62]. In primary human retinal endothelial cell (HREC) culture, SDF-1α increased HREC migration while AMD3100 (an inhibitor of CXCR4) and EGTA (a Ca^++^ chelator) effectively decreased it. Similarly, curcumin decreased SDF-1α-induced HRECs migration. The mechanism of action could be upstream blockage of Ca^2+^ influx which is an important signal for HREC migration or reduction of downstream PI3-K/AKT signals. Thus, curcumin inhibits HREC migration by interfering with SDF-1alpha/CXCR4 signaling [63]. Moreover, docking studies revealed that curcumin inhibited HIV replication through binding with CXCR4 [64].

Both resveratrol and quercetin inhibited angiogenesis in vitro where they inhibited growth of bovine aorta endothelial (BAE) cells, but resveratrol inhibited BAE cells migration more effectively than quercetin. Also, quercetin inhibited the tube formation of vascular endothelial cells. Thus, resveratrol and quercetin could be useful for tumor angiogenesis [65]. Resveratrol retarded development of cardiovascular disease by affecting certain risk factors such as diabetes or atherosclerosis. On human coronary arterial endothelial cells (HCAECs) resveratrol had anti-inflammatory effects via induction of autophagy which confers cardio-protection [66]. In retinal pigment epithelial cells, resveratrol effectively decreased phosphorylation of nuclear factor (NF)-κB (an upstream activator of CXCR4) resulting in suppression of CXCR4 expression and downregulation of vascular endothelial growth factor (VEGF) secretion. Resveratrol also suppressed VEGF secretion induced by SDF-1 [67]. In BTBR and C57BL/6 J mice, resveratrol treatment (20 and 40 mg/kg) significantly decreased CXCR production and expression in CD4^+^ T cells and decreased mRNA expression levels of CCR and CXCR in brain and spleen tissues [68]. Resveratrol and its combination with AMD3100 as a CXCR4 inhibitor significantly inhibited dihydrotestosterone (DHT)-induced progression of prostate cancer cell line via affecting the CXCR4 pathway [69]. Unfortunately, resveratrol has low bioavailability and solubility [70]. Quercetin, in cultured human endothelial cells, showed anti-oxidative and anti-inflammatory properties and protected against lipid peroxidation. Quercetin also decreased the transcriptional activity of NF-κB in human hepatocytes. In a humanized inflammation model (human CRP transgenic mice), quercetin inhibited IL-1β-induced CRP expression (CRP is a cardiovascular risk marker). In a humanized atherosclerosis model (ApoE*3Leiden transgenic mice), quercetin significantly attenuated atherosclerosis. Quercetin affected vascular cell proliferation thus reduced growth of atherosclerotic lesion. Quercetin systemically decreased expression of human CRP and cardiovascular risk factors and locally in aorta showed anti-proliferative effect resulting in attenuation of atherosclerosis [71]. Unfortunately, like curcumin and resveratrol, quercetin has low bioavailability, and its absorption can be affected by macronutrients [72].

Eucalyptol decreases lipopolysaccharide-induced cytokine production via action of NF-κB, TNF-α, IL-6, and IL-1β, and the ERK pathway. Thus, eucalyptol could be a potentially significant agent for treatment of chronic diseases [73]. In the Aβ (25-35) treated PC12 cells (a clonal cell line from an induced, transplantable rat pheochromocytoma), treatment with 1,8-cineole lowered the levels of TNF-α, IL-6, and IL-1β and the expression of nitric oxide synthase (NOS)-2, COX-2 and NF-κB indicating its protective effects and potential benefits in neurodegenerative disease [74]. In both anesthetized and conscious normotensive rats, intravenous 1,8-cineole dose-dependently decreased mean aortic pressure and only at the highest dose decreased heart rate. This hypotension is mostly due to an active vascular relaxation rather than inhibition of the sympathetic system [75].

Although all compounds, except quercetin, have more interactions with CXCR4 than with CXCR7, but the differences in the docking scores are minimal and do not imply great differences in the docking behavior. When a ligand is docked into a receptor protein, the interactions between them act between atoms which are not linked by covalent bonds i.e., there are non-bonded (non-covalent) interactions or bonds. The interactions between them could be hydrogen, electrostatic, van der Walls, polar-polar, and bi-bi staking and in some cases ionic bonds interactions which are all reversible and transient [76]. This means that the binding of the test natural compounds can be displaced by other antagonists or the endogenous ligands (CXCL11 and CXCL12) if their concentrations exceed those of the test natural compounds. Generally, based on the docking scores it can be decided which compound, compared with others, is the most effective toward the desired protein or receptor. In our study, we used a rigid protein and flexible ligands to evaluate the docking of the compounds inside the active site of protein so that the receptors remain unchanged and retain their original structure. The stability of compounds inside the cavity and determination of the duration of their binding to the receptors can be evaluated by using the molecular dynamics, which simulates the dynamic behavior of molecular systems as a function of time [61], but unfortunately, we do not have the programs which can do such study in addition to being time-consuming because it takes too much time to evaluate the stability of compounds inside the cavity to 500 ns or not. This is considered a limitation of our study. Moreover, detection of effect of the conformational rearrangements occurring in the CXCR4 and CXCR7 receptors as a result of their homodimerization and heterodimerization on binding to the test compounds is a second limitation of this study. However, these are good ideas for further studies.

The ADME prediction showed that the compounds possess several desirable ADME properties. The bioavailability radar showed that all compounds are predicted orally bioavailable. The consensus log *P*_o/w_, the classical descriptor for lipophilicity, showed that all the tested compounds are lipophilic because their values are above zero. Curcumin showed the highest lipophilicity and moderate solubility in water while quercetin, which showed the lowest lipophilicity, is soluble in water [77]. The four compounds have high GI absorption while only trans-resveratrol and (1 s,4 s)-Eucalyptol can penetrate the BBB. None of the compounds is a substrate of P-glycoprotein indicating absence of any active efflux for them from the gastrointestinal wall to the lumen or from the brain [35]. While (1 s,4 s)-Eucalyptol has no interactions with cytochromes P450 (CYP) major isoenzymes, other compounds can inhibit a certain CYP isoenzyme or another leading to adverse effects or drug interactions [36]. The skin permeability coefficient (*K*_p_) is linearly correlated with molecular size and lipophilicity and the more negative the log *K*_*p*_ (cm/s), the less skin permeation [37]. Thus, quercetin has the lowest while (1 s,4 s)-Eucalyptol has the highest skin penetration which allows its application through skin massage. Moreover, the four compounds are considered to pass all the five commonly used drug-likeness rules and thus can be categorized as drug-like compounds and all of them reached a bioavailability score value of 0.55. All compounds, except (1 s,4 s)-Eucalyptol, maintain undesirable functional groups which could lead to carcinogenic, mutagenic, and hepatotoxic effects [78]. In agreement with previous studies, curcumin, trans-resveratrol, and quercetin were found non-cardiotoxic [79–81]. Although (1 s,4 s)-Eucalyptol showed a potential weak to moderate cardiotoxicity, but to our knowledge, there is no previous reports of cardiotoxicity of (1 s,4 s)-Eucalyptol. It was reported to cause hypotension [82], however this effect could be useful for hypertensive patients. “Leadlikeness” is similar to drug-likeness, but it focuses on physicochemical limits that define a good lead [46]. All compounds except quercetin have violations, but leads (by definition) can be subjected to chemical modifications which most likely increase their size and lipophilicity, thus leads are needed to be smaller and less hydrophobic than drug-like molecules [83]. All compounds have low “synthetic accessibility” score which is essential in the selection process of the most promising virtually-tested molecules that can be synthetized and provided to biological assays [47].

Based on the frontier molecular orbital theory, energies of HOMO and LUMO play an important role in chemical reactivity. The electronic absorption relates to the transition from the ground to the first excited state and mainly described by one electron excitation from HOMO to LUMO [84]. The HOMO-LUMO gap is related to the global chemical reactivity parameters. Large HOMO-LUMO gap is responsible for high kinetic stability [85]. In the current study, curcumin showed the highest values of ∆ E, ionization potential, absolute electronegativity, absolute and global hardness, global electrophilicity, and additional electronic charge. In another hand, curcumin showed the lowest values in electron affinity, and absolute and global softness which may be attributed to its high expected activity toward the CXCR4 and CXCR7 receptors.

The molecular dynamics simulation with molecular-mechanics-generalized born or Poisson-Boltzmann and surface area (MM/GBSA or MM/PBSA) methods are famous ways to evaluate free energy of binding of small ligands to biological macromolecules [86]. In the current study, MM/PBSA based methods was used for calculation of different interaction energies between the protein and inhibitor during the course of 100 ns MD simulations. It was observed that the free energy of Van der Waals was around − 218.226 kJ/mol, along with electrostatic component of − 27.723 kJ/mol. The free energy associated with the solvent accessibility was around − 18.424 kJ/mol and the free energy of binding was around − 282.797 kJ/mol validating the outcomes generated from the molecular docking. For molecular dynamics, we did that calculation to validate the probability of the compound with the highest docking score (curcumin) to be a good candidate to the active site. We did not make MD calculations for the other three compounds due to their low activities in addition to time, budget, and server limitations.

The current study provides novel visions into regulation of the CXCR4 and CXCR7 receptors by these natural compounds enabling possible explanations for their behavior. Use of these compounds either orally (for the three polyphenols) or by inhalation or skin massage (for eucalyptol) is considered non-invasive, cheap, easily applicable, and cost-effective approach. Therefore, repurposing of both curcumin and eucalyptol against coronary artery disease to combat chronic inflammation and oxidative stress and improve side effects such as insomnia and nausea is suggested for clinical trials.

Limitations of this study include: (1) The 100 ns scale molecular dynamics simulation has been done only for curcumin (the compound having the best docking score). (2) The stability of compounds inside the cavity and determination of the duration of their binding to the receptors can be evaluated by using the molecular dynamics, but unfortunately, we do not have the programs which can do such study in addition to time constraints because it takes too much time to evaluate the stability of compounds inside the cavity to 500 ns. (3) Detection of effect of the conformational rearrangements occurring in the CXCR4 and CXCR7 receptors as a result of their homodimerization and heterodimerization on binding to the test compounds. Further studies are recommended to address these limitations.

Strengths of this study include: (1) To our knowledge, this is the first attempt to find binding interactions of these natural agents with CXCR4 and CXCR7 receptors and to predict their druggability. Curcumin showed the top binding interaction against active sites of CXCR4 and CXCR7 receptors, with the best safety profile, followed by quercetin, resveratrol, and eucalyptol. All compounds demonstrated drug-like properties. Eucalyptol has promising potential because it can be used by inhalation or skin massage. (2) The current study provides novel visions into regulation of the CXCR4 and CXCR7 receptors by these natural compounds enabling possible explanations for their behavior. Recently, it was reported that CXCR7 activation may be a possible therapeutic target in cases of ischemic myocardium.

Some previous studies have reported relationships between CXCR4 and curcumin, quercetin, resveratrol as mentioned before but to the best of our knowledge, this is the first study that reports binding interactions of eucalyptol with CXCR4 and interactions of the four compounds with the CXCR7 receptors. But unfortunately, curcumin, resveratrol, and quercetin have low bioavailability. This is the main clinical gap facing these compounds; thus, studies and clinical trials are needed to overcome this issue.

## Conclusions

Molecular modeling procedures were used to screen for the potential regulatory effects of four cardiovascular active natural compounds; three polyphenols and a terpene; on the CXCL12/CXCR4/CXCR7 pathway. After a virtual screening against the active sites of CXCR4 and CXCR7 receptor, curcumin showed the top binding interaction, with the best safety profile, followed by quercetin, resveratrol, and lastly eucalyptol. The ADME prediction using the SwissADME server showed that all compounds possess drug-like properties. Only (1 s,4 s)-Eucalyptol has potential weak cardiotoxicity. The results of the thermochemical and molecular orbital analysis and the molecular dynamics simulation validated the outcomes of the molecular docking study. The four compounds have low oral bioavailabilities and thus eucalyptol has a promising potential because it can be used by inhalation or skin massage. These results may provide novel visions into CXCR4 and CXCR7 regulation enabling a possible explanation of behavior shown by these natural compounds. Use of these compounds either orally (for the three polyphenols) or by inhalation or skin massage (for eucalyptol) is considered non-invasive, cheap, easily applicable, and cost-effective approach. Therefore, repurposing of both curcumin and eucalyptol against CAD disease to combat chronic inflammation and oxidative stress and improve side effects such as insomnia and nausea is suggested for clinical trials. To the best of our knowledge, this is the first structural attempt to find binding interactions of natural anti-inflammatory agents; curcumin, resveratrol, quercetin, and eucalyptol with the CXCR4 and CXCR7 receptors and to predict their drug-likeness properties.

## Supplementary Information

**Additional file 1: Fig. S1.** The 2D and 3D docking interaction of Curcumin, Trans-resveratrol, Quercetin, and (1 s,4 s)-Eucalyptol with protein 3ODU of CXCR4 receptor. **Fig. S2.** The 2D and 3D docking interaction of Curcumin, Trans-resveratrol, Quercetin, and (1 s,4 s)-Eucalyptol with protein 6K3F of CXCR 7 receptor.

## Data Availability

The data and materials are available from the corresponding author on reasonable request.

## Abbreviations

ADME/T: Absorption, distribution, metabolism, and excretion/toxicity
CXCL12: Chemokine (C-X-C motif) ligand 12
SDF-1: Stromal-cell-derived factor-1
GPCR: G protein coupled receptors
PI3K: Phosphoinositide-3 kinase
PLC/IP3: Phospholipase C/inositol 1,4,5, triphosphate
ERK1/2: Extracellular regulated kinase 1/2
CAD: Coronary artery disease
EOCs: Endothelial outgrowth cells
NF-κB: Nuclear factor-κB
MAPK: Mitogen-activated protein kinases
COX: Cyclooxygenase
TNF-α: Tumor necrosis factor-α
IL-1b: Interleukin 1b
MOE: Molecular environment
MW: Molecular weight
MR: Molecular refractivity
TPSA: Topological polar surface area
PSA: Polar surface area
Log *P*_o/w_: Log partition coefficient between *n*-octanol and water
Log *S*: Log molar solubility in water
ESOL: Estimated solubility
hERG: Human Ether-à-go-go-Related Gene
QSAR: Quantitative structure-activity relationship
SDF: Structure data file
SMILES: Simplified molecular-input line-entry system
QM: Quantum mechanical
DFT: Density functional theory
MD: Molecular dynamics
MM/GBSA or MM/PBSA: Molecular-mechanics-generalized born or Poisson-Boltzmann and surface area
H-bonds: Hydrogen bonds
RMSD: Root mean square deviations
Rg: Radius of gyration
AD: Applicability domain
HOMO: Highest occupied molecular orbital
LUMO: Lowest unoccupied molecular orbital
∆E: HOMO–LUMO energy gap
I: Ionization potential
A: Electron affinity
χ: Absolute electronegativities
ɳ: Absolute hardness
σ: Absolute softness
Pi: Chemical potentials
S: Global softness
H: Global hardness
ΔH: Enthalpies
ΔG: Free energies
BBB: Blood-brain barrier
P-gp: Permeability glycoprotein
CYP: Cytochrome P450
Log *K*_p_: Log skin permeability coefficient
MMP-9: Matrix metalloproteinase-9
CRP: C-reactive protein
HREC: Human retinal endothelial cell
BAE cell: Bovine aorta endothelial cell
HCAECs: Human coronary arterial endothelial cells
NF-κB: Nuclear factor-κB
NOS: Nitric oxide synthase
