# Supplementary material for: Interactions of selected cardiovascular active natural compounds with CXCR4 and CXCR7 receptors: a molecular docking, molecular dynamics, and pharmacokinetic/toxicity prediction study
Source: BMC Complement Med Ther. 2022 Feb 4;22:35. doi: 10.1186/s12906-021-03488-8 (PMC8817505; doi:10.1186/s12906-021-03488-8)
Supplement: Supplementary file 1 — Additional file 1: Fig. S1. The 2D and 3D docking interaction of Curcumin, Trans-resveratrol, Quercetin, and (1 s,4 s)-Eucalyptol with protein 3ODU of CXCR4 receptor. Fig. S2. The 2D and 3D docking interaction of Curcumin, Trans-resveratrol, Quercetin, and (1 s,4 s)-Eucalyptol with protein 6K3F of CXCR 7 receptor. [file 12906_2021_3488_MOESM1_ESM.docx]

**Supplementary Figures**

| **Curcumin** | 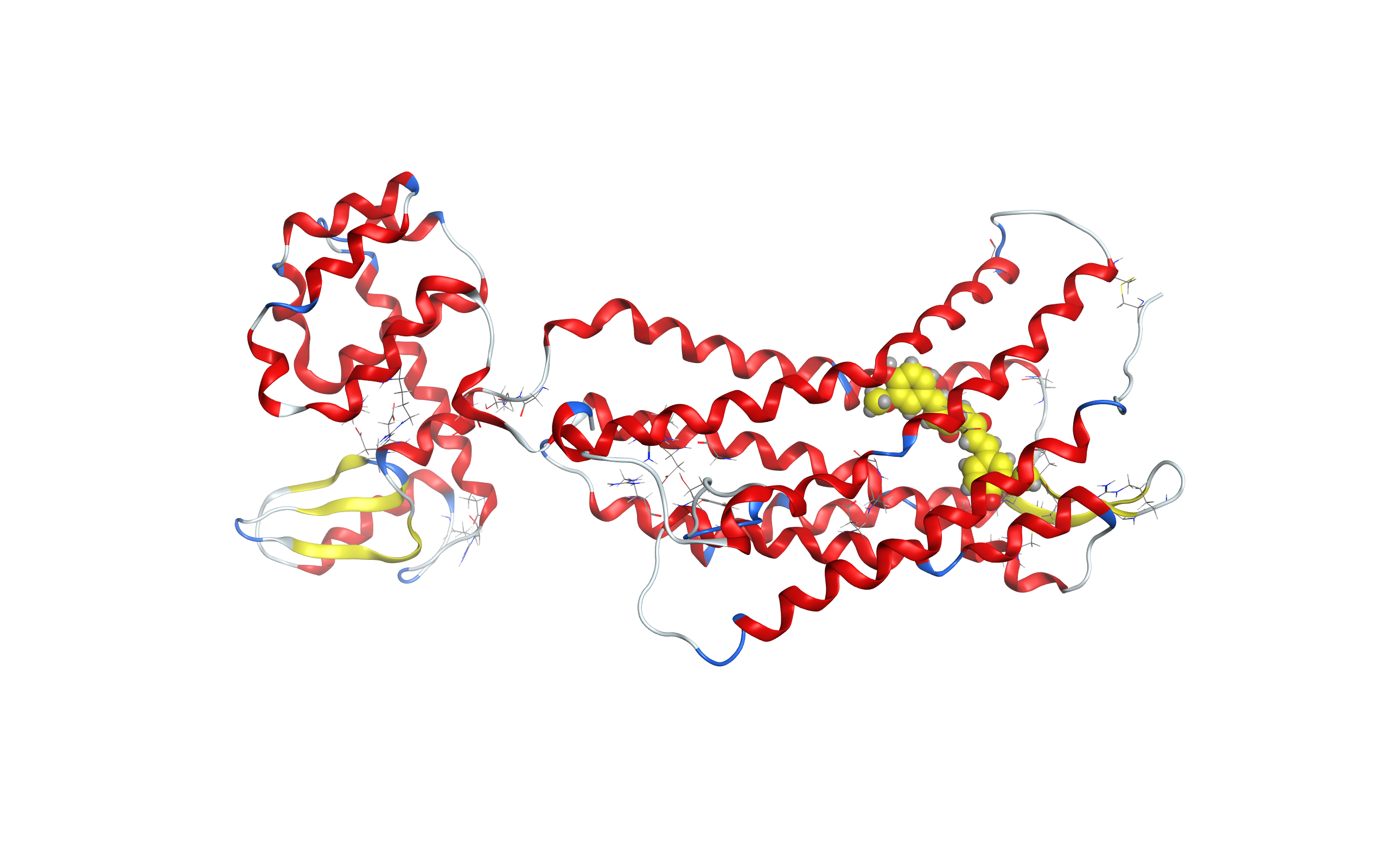 |
| --- | --- |
| **Trans-resveratrol** | 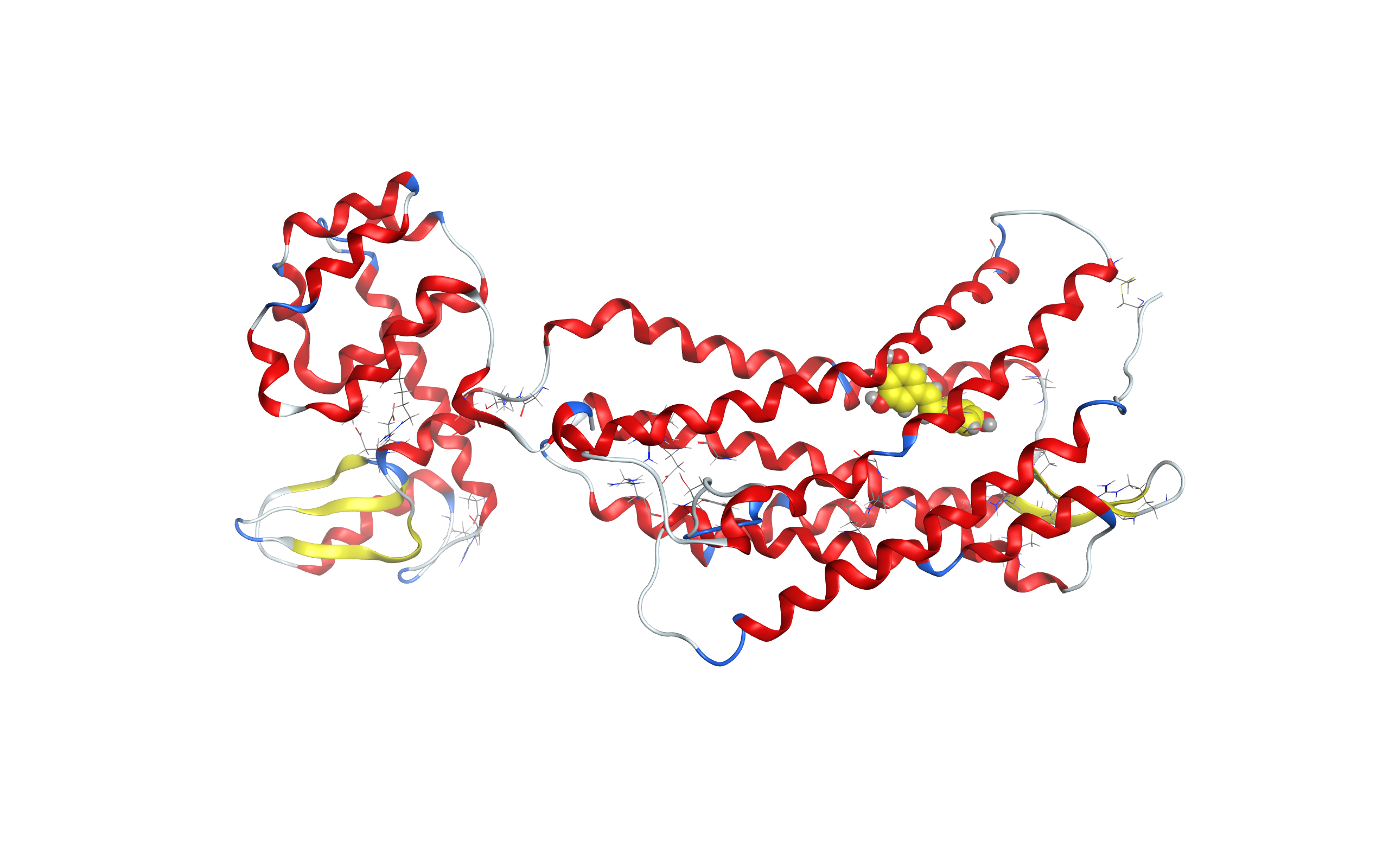 |
| **Quercetin** | 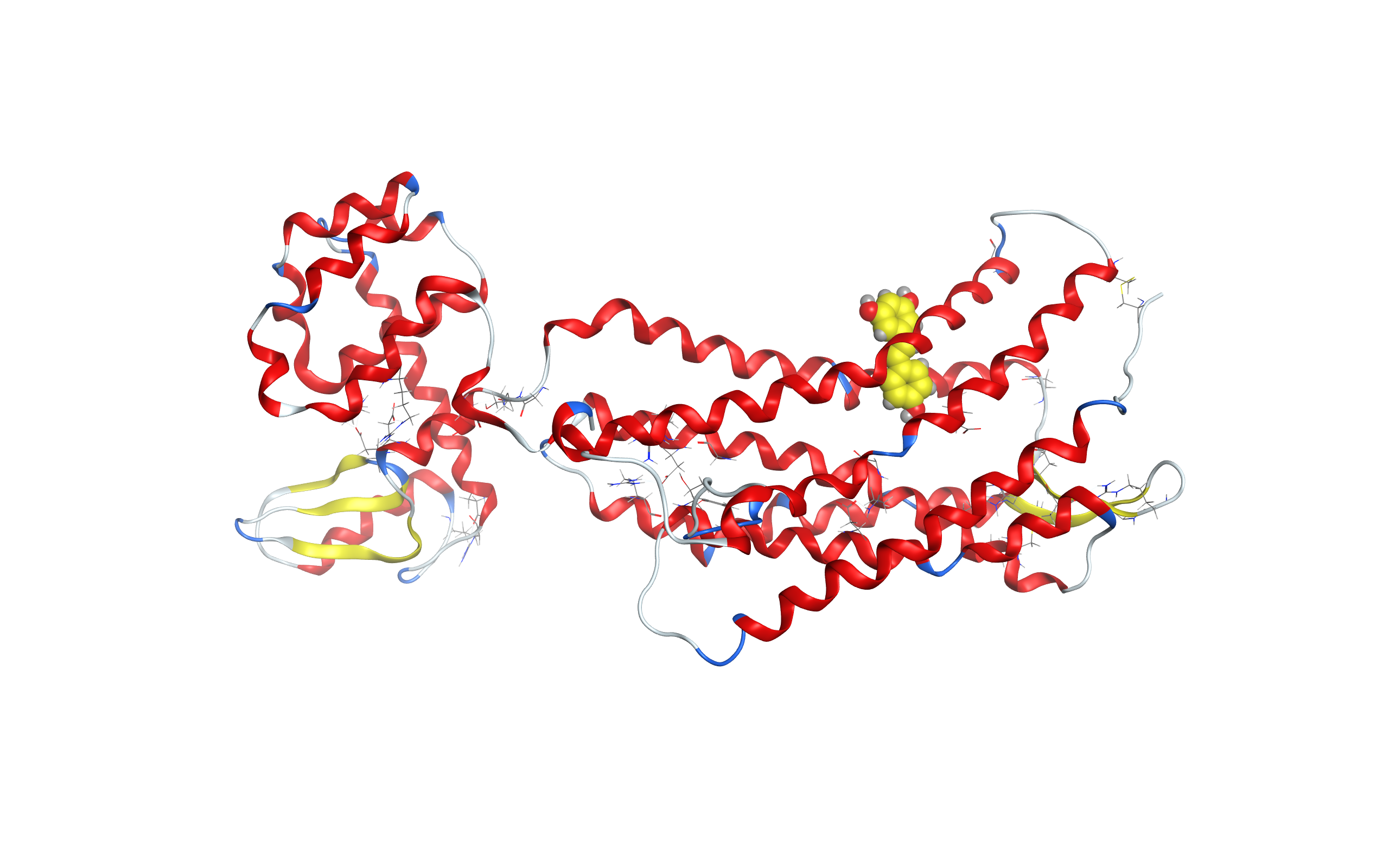 |
| **(1s,4s)-Eucalyptol** | 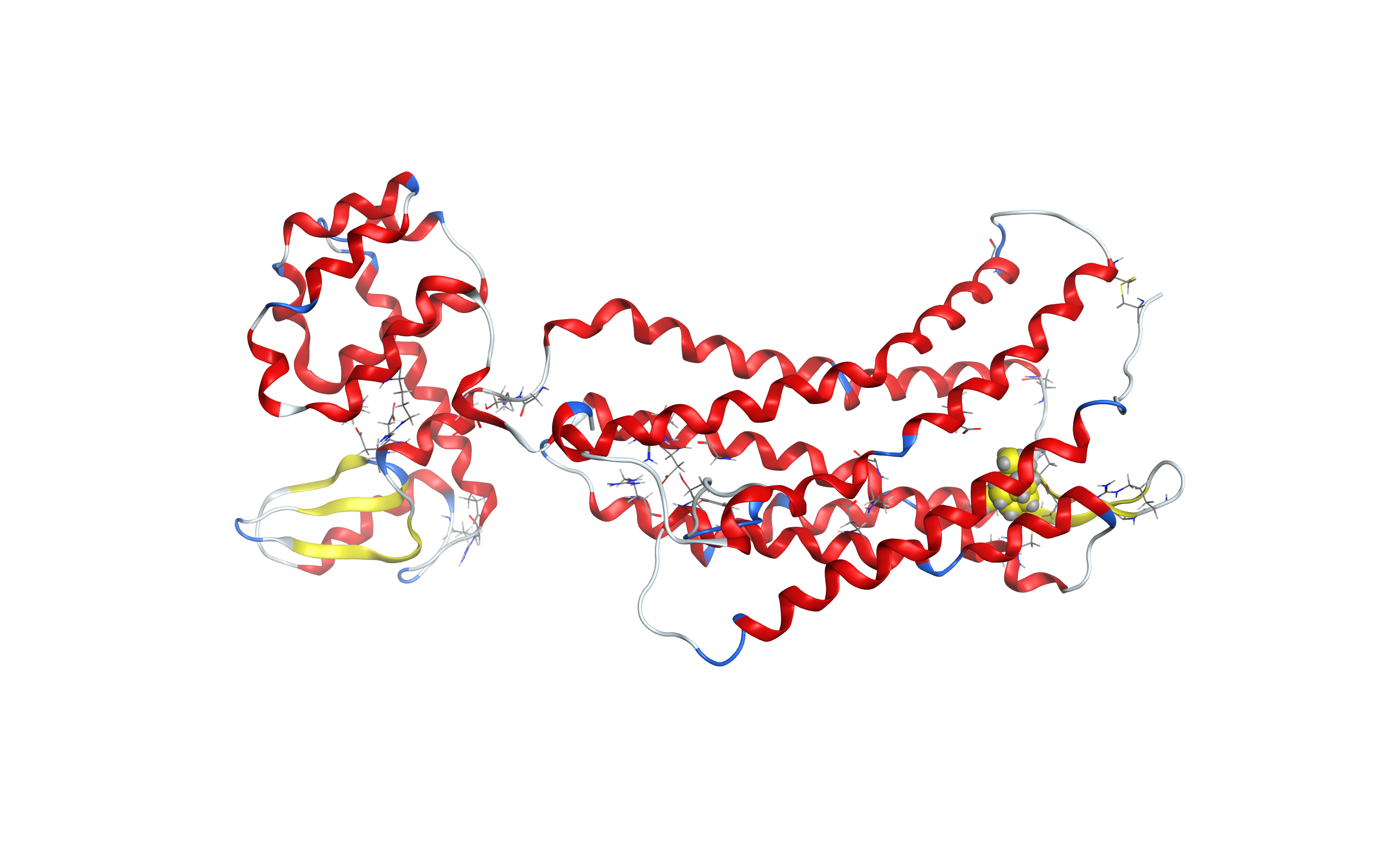 |

**Fig. S1** The 2D and 3D docking interaction of Curcumin, Trans-resveratrol, Quercetin, and (1s,4s)-Eucalyptol with protein 3ODU of CXCR4 receptor

| **Curcumin** | 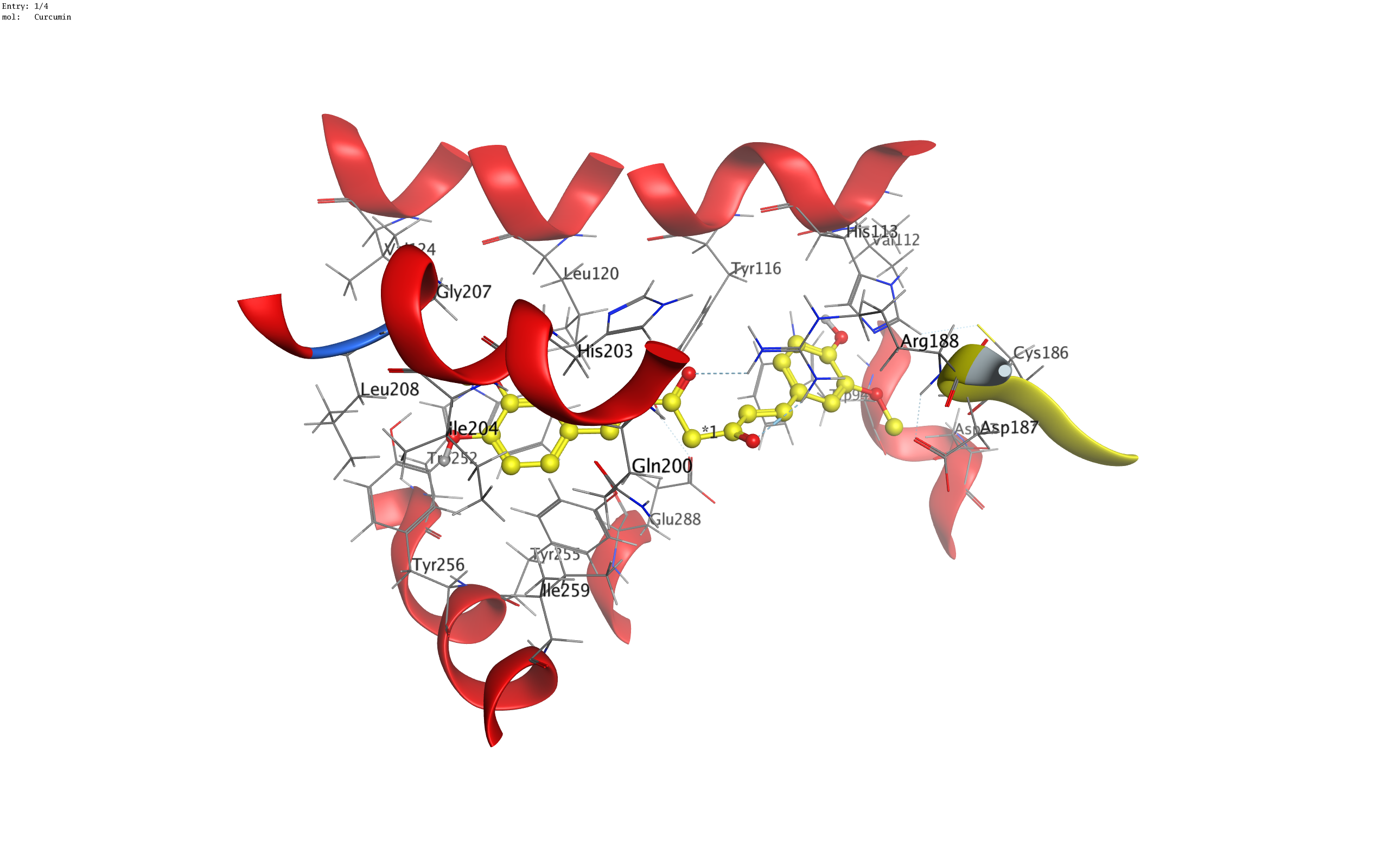 |
| --- | --- |
| **Trans-resveratrol** | 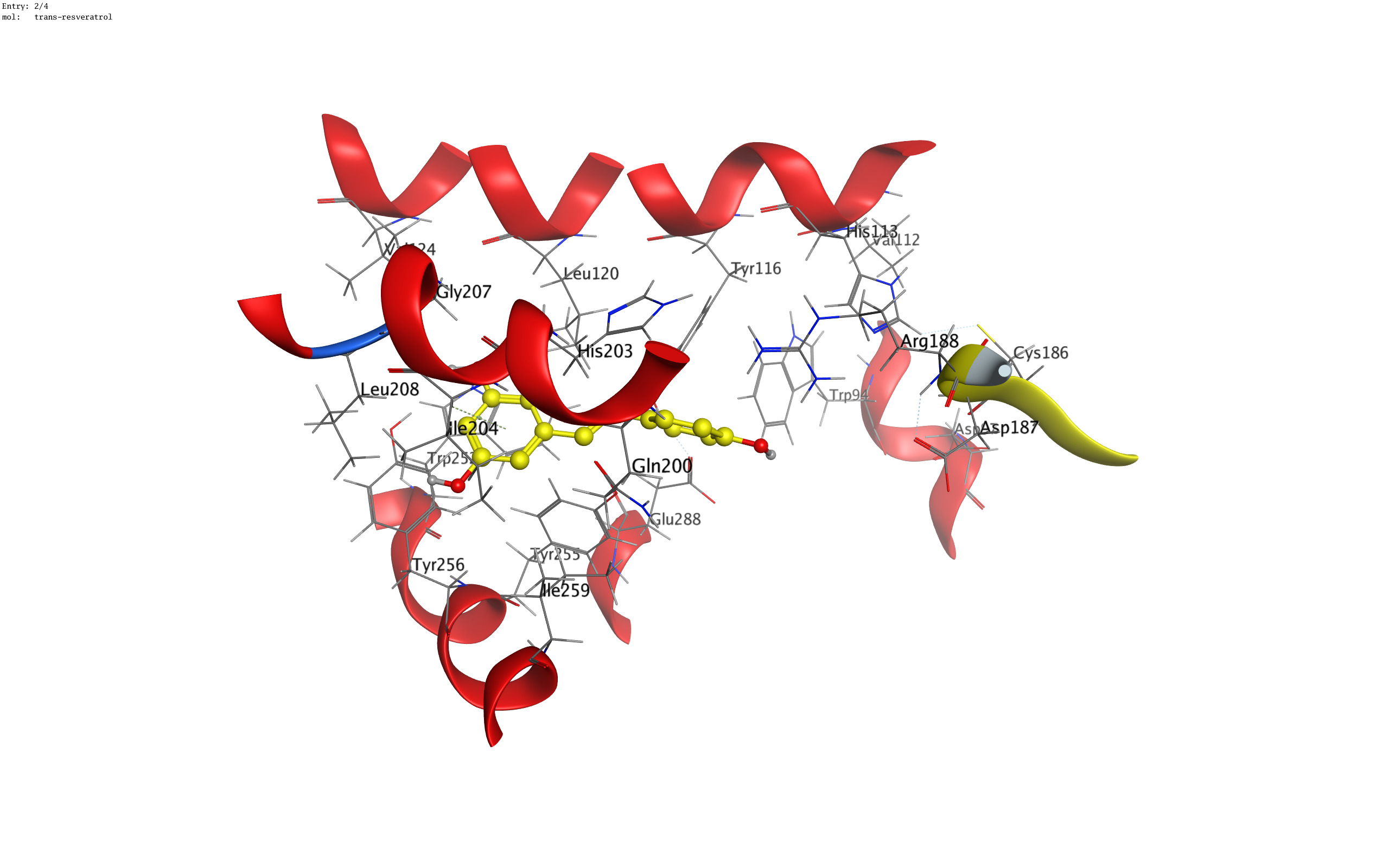 |
| **Quercetin** | 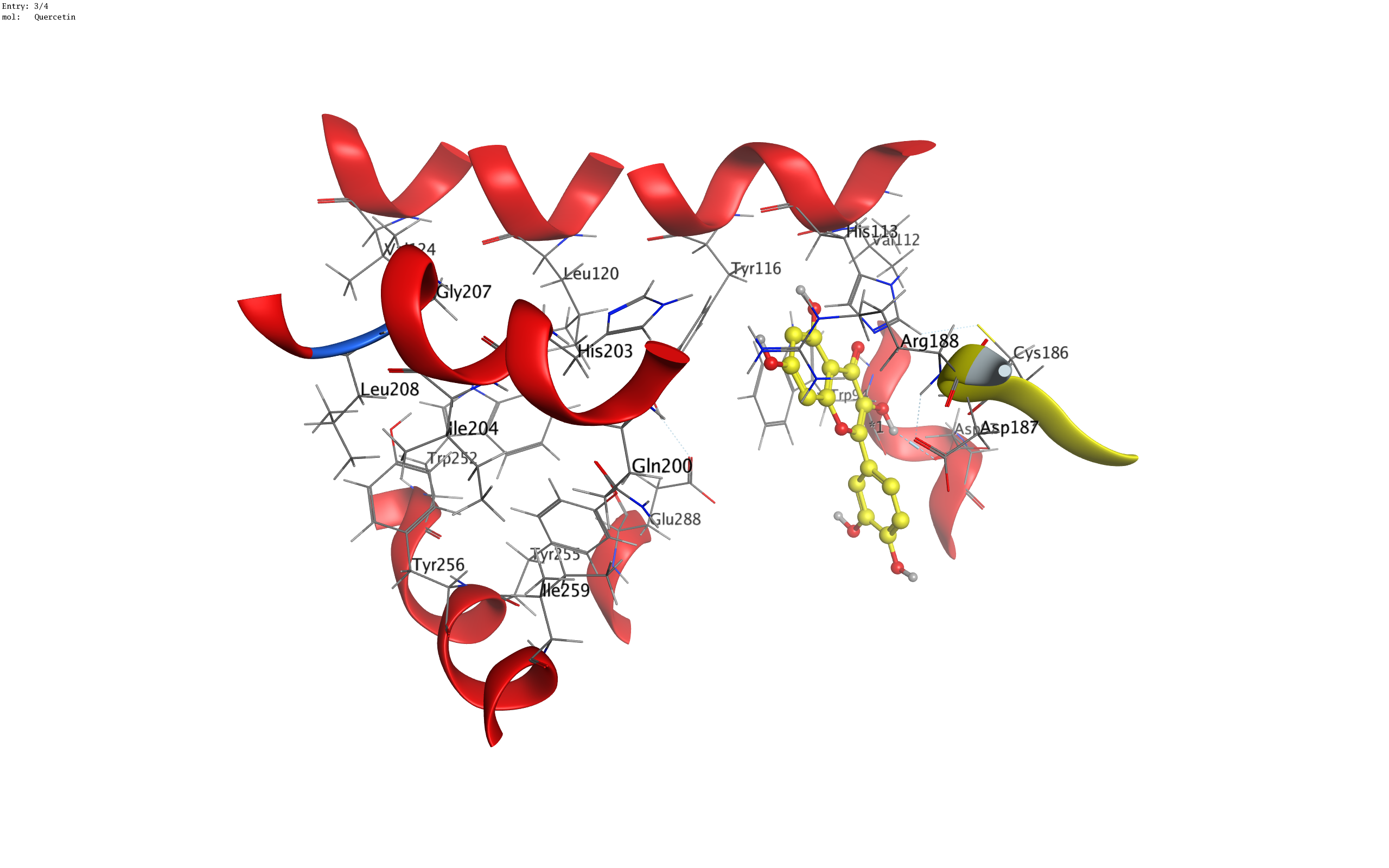 |
| **(1s,4s)-Eucalyptol** | 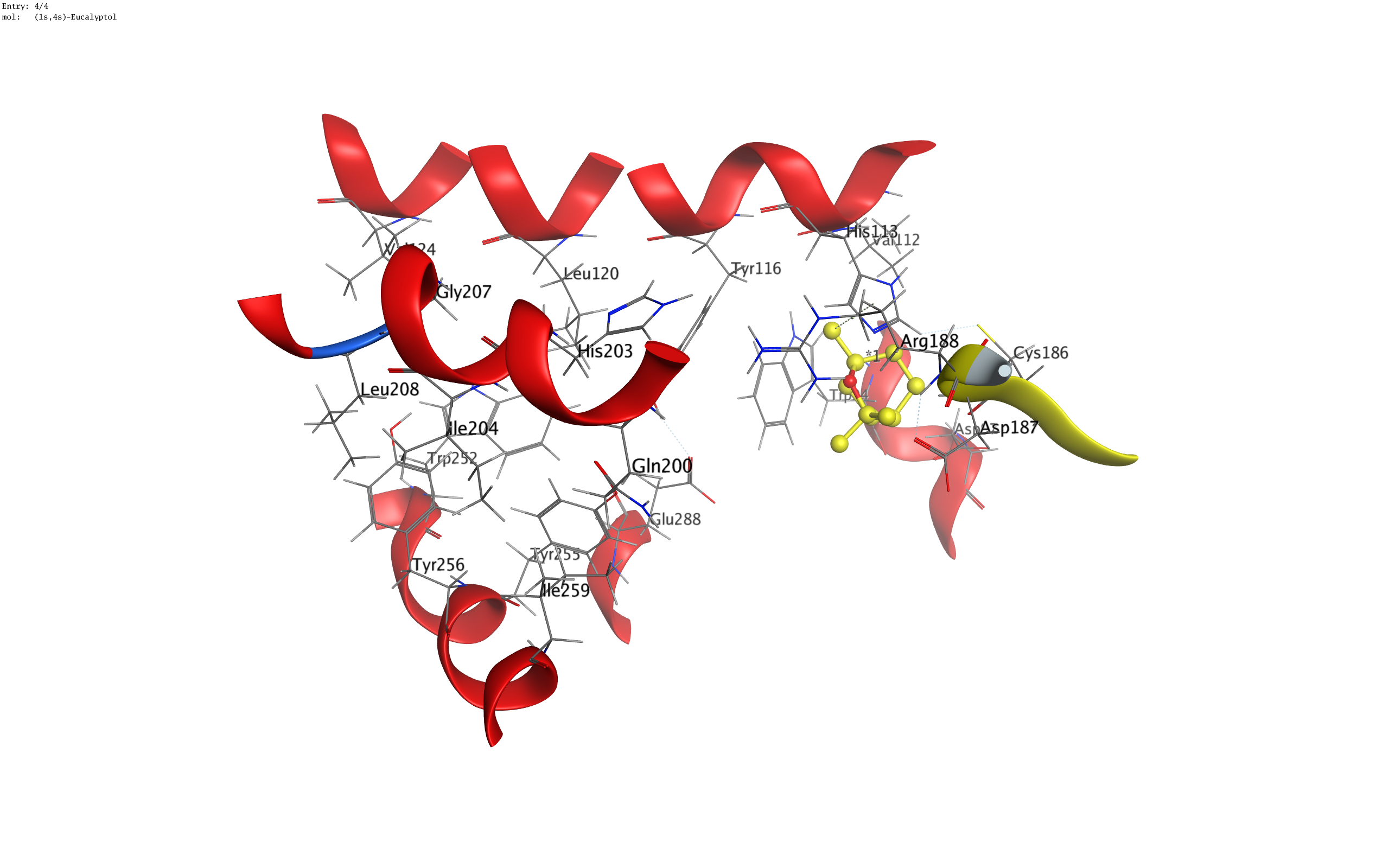 |

**Fig. S2** The docking sites of (A) Curcumin, (B) Trans-resveratrol, (C) Quercetin, and (D) (1s,4s)-Eucalyptol with protein 3ODU of CXCR4 receptor

| **Curcumin** | 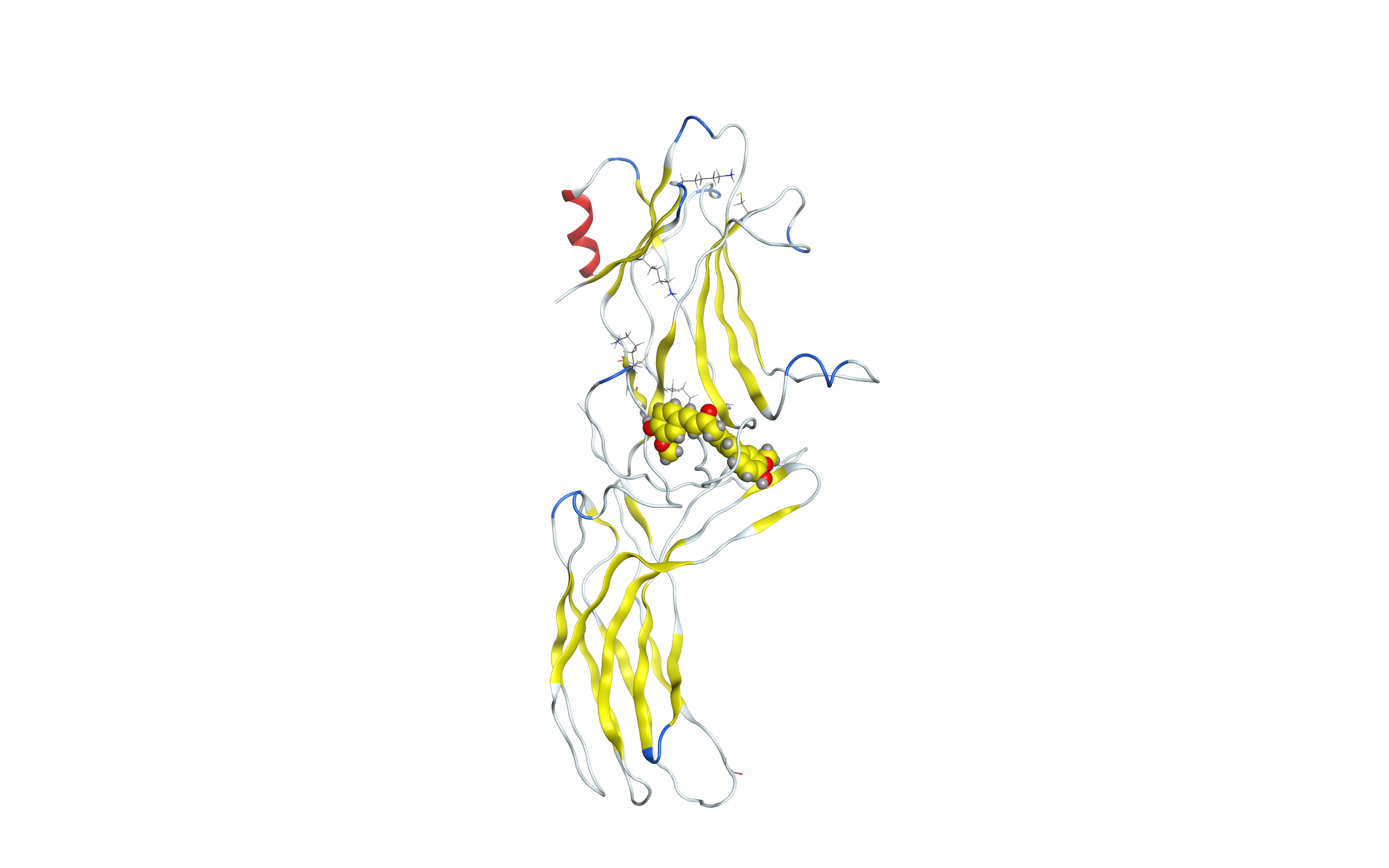 |
| --- | --- |
| **Trans-resveratrol** | 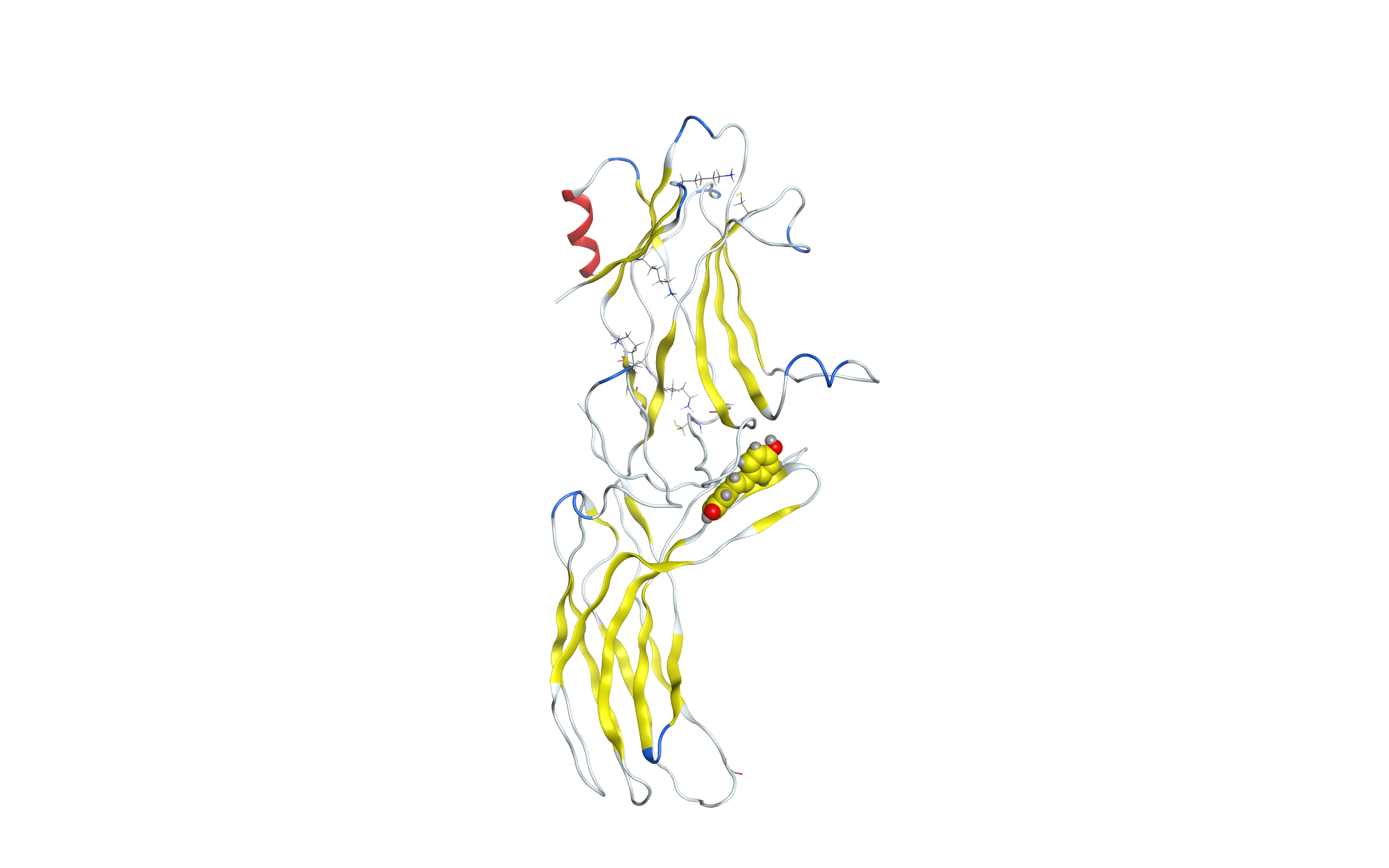 |
| **Quercetin** | 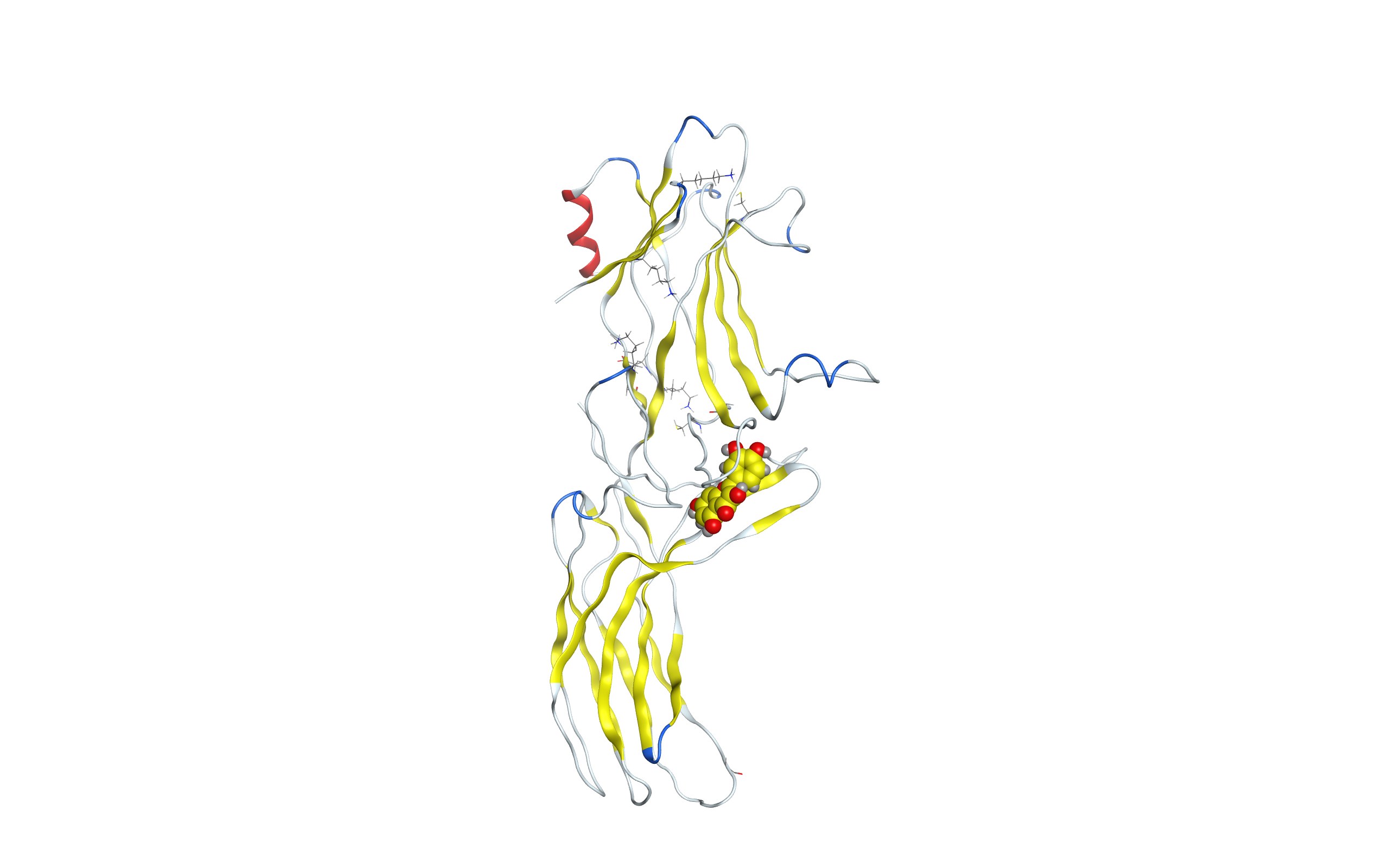 |
| **(1s,4s)-Eucalyptol** | 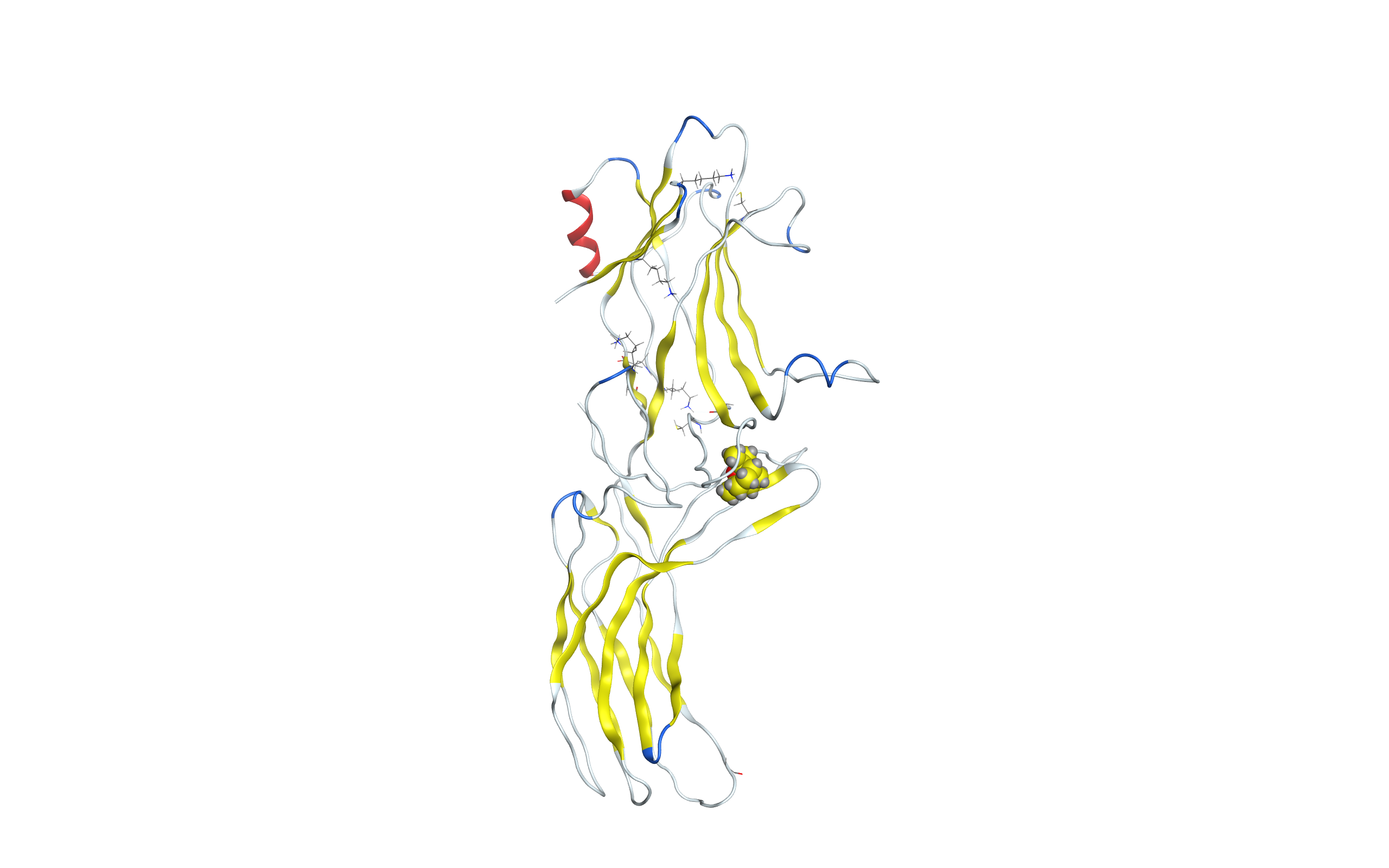 |

**Fig. S3** The 2D and 3D docking interaction of Curcumin, Trans-resveratrol, Quercetin, and (1s,4s)-Eucalyptol with protein 6K3F of CXCR 7 receptor

| **Curcumin** | 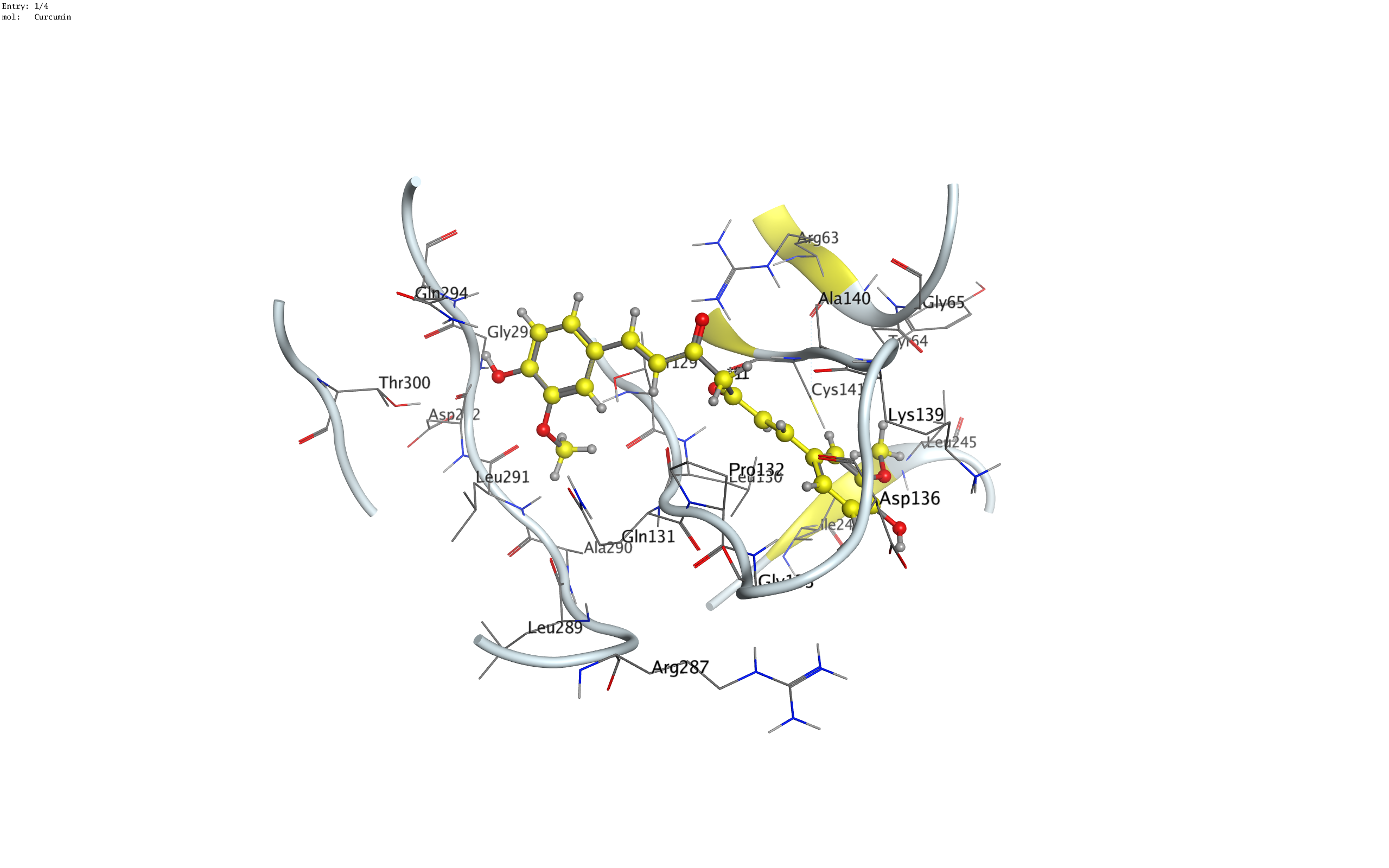 |
| --- | --- |
| **Trans-resveratrol** | 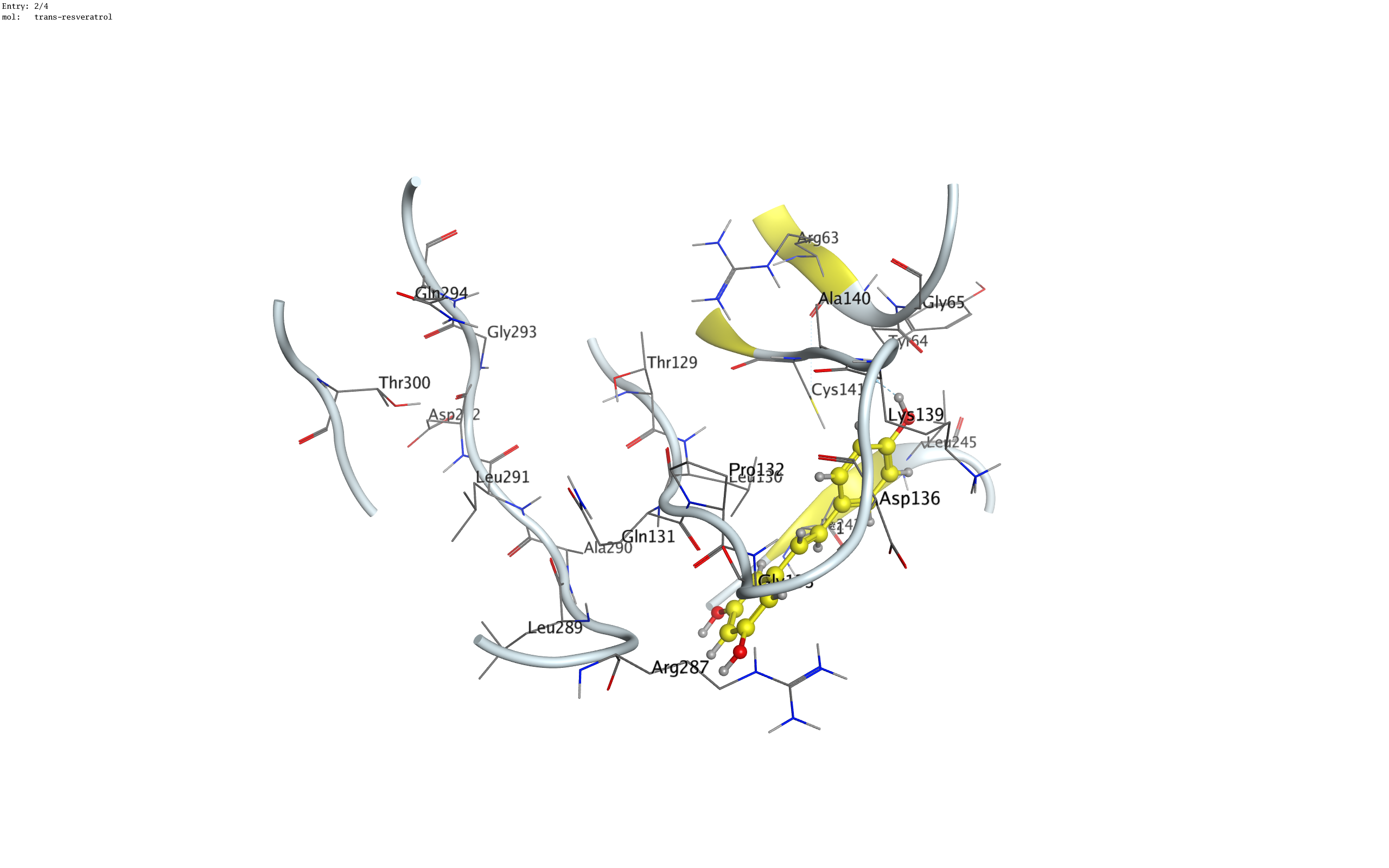 |
| **Quercetin** | 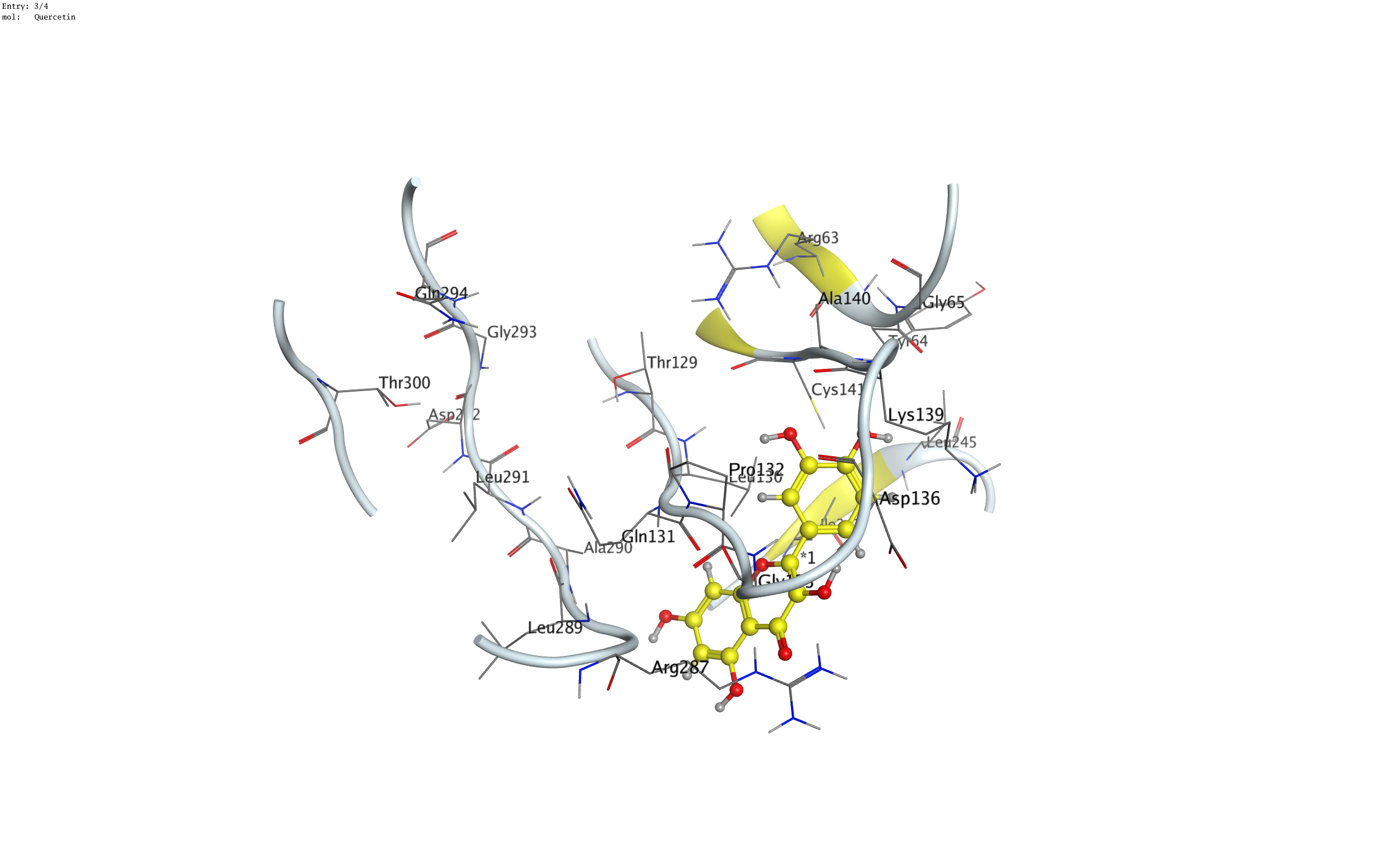 |
| **(1s,4s)-Eucalyptol** | 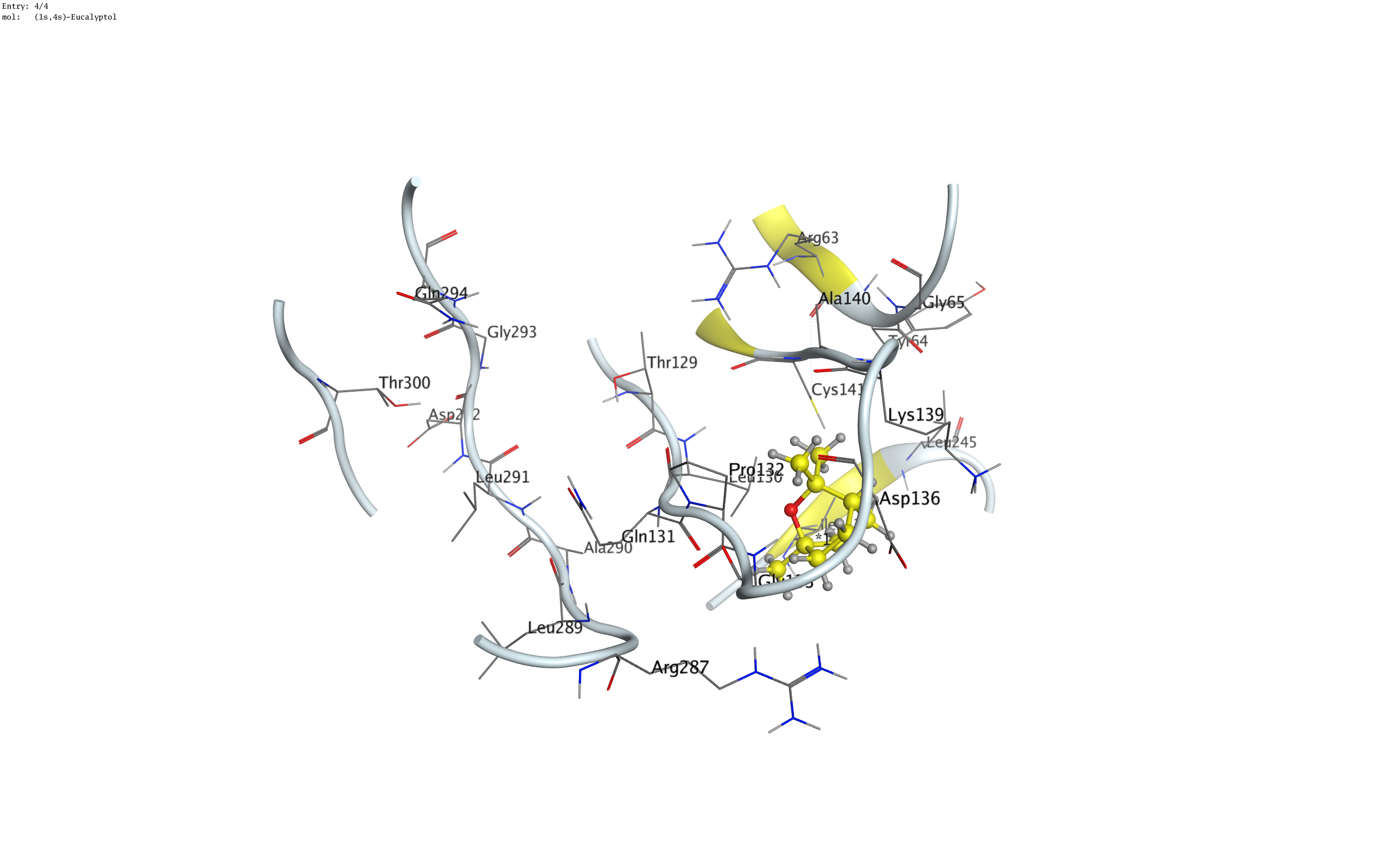 |

**Fig. S4** The docking sites of (A) Curcumin, (B) Trans-resveratrol, (C) Quercetin, and (D) (1s,4s)-Eucalyptol with protein 6K3F of CXCR7 receptor

| 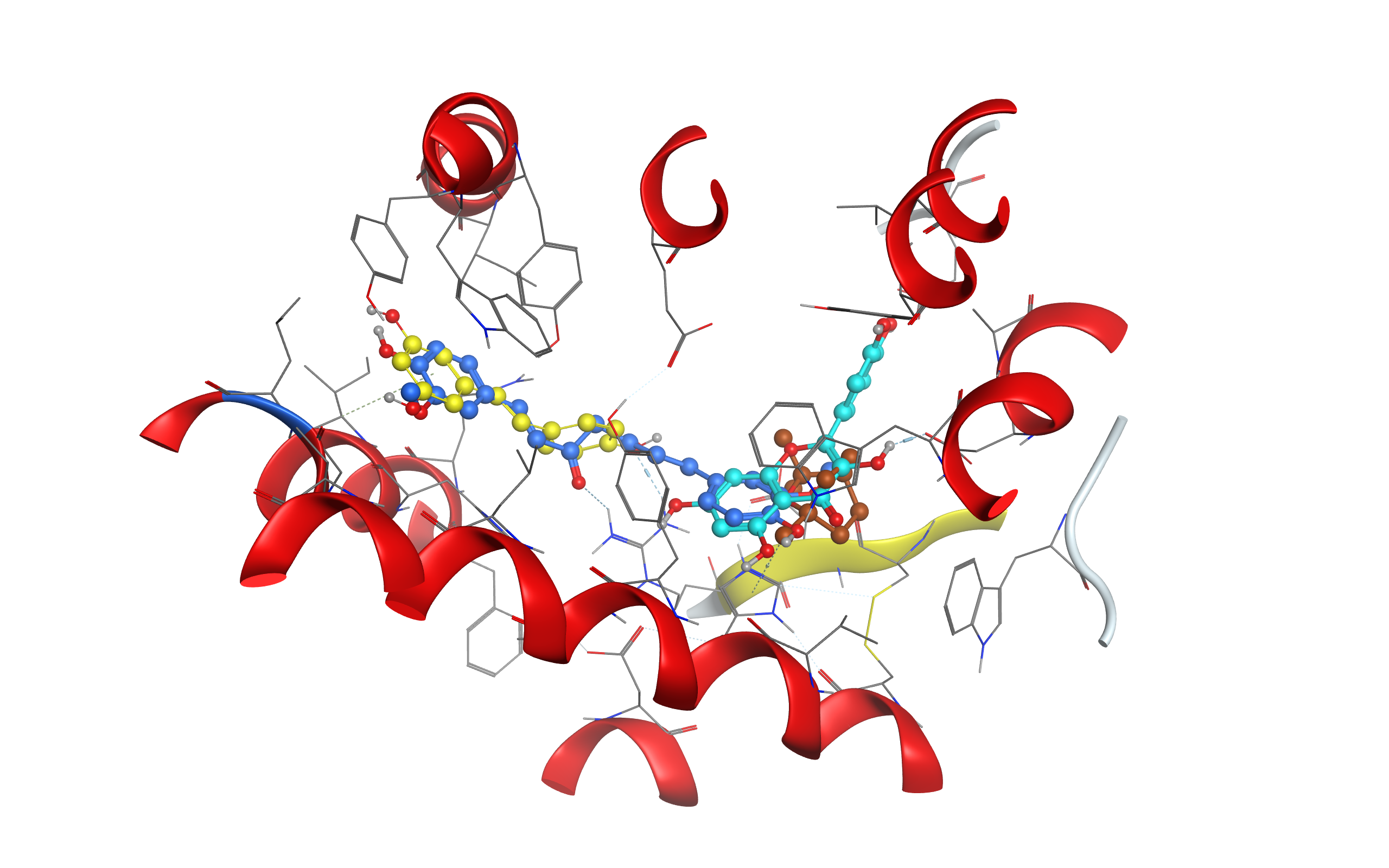 | |
| --- | --- |
| **Curcumin** | 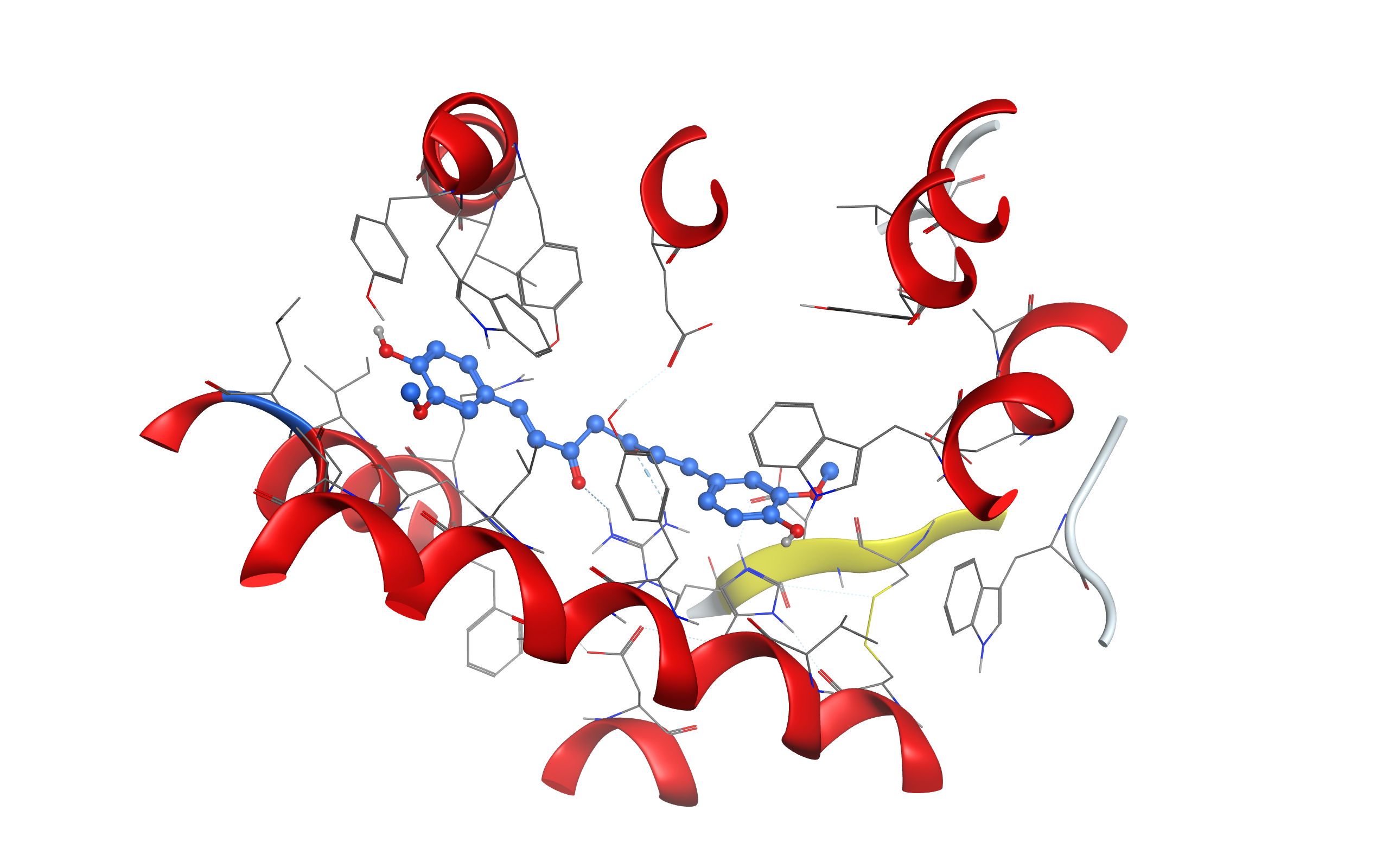 |
| **Trans-resveratrol** | 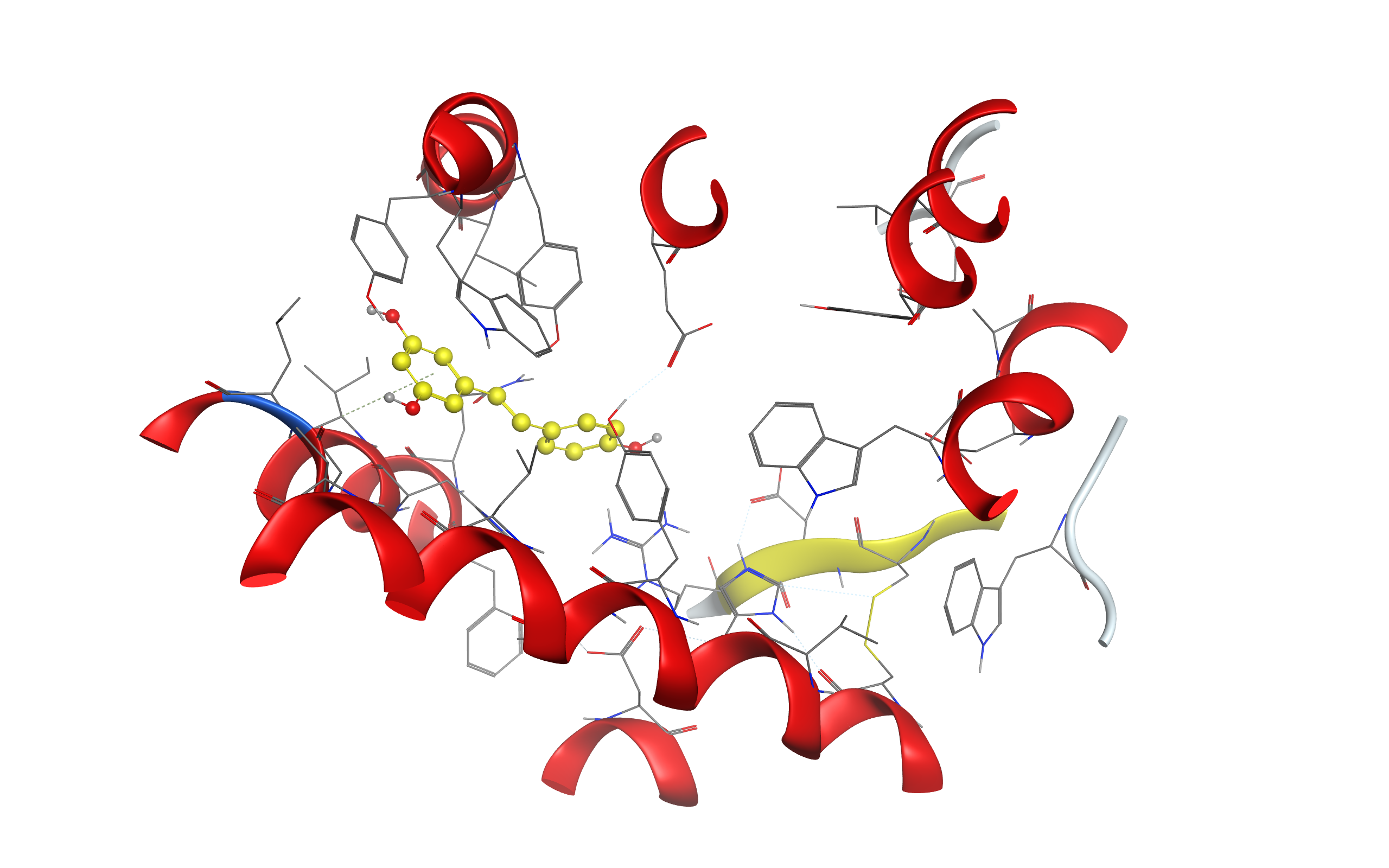 |
| **Quercetin** | 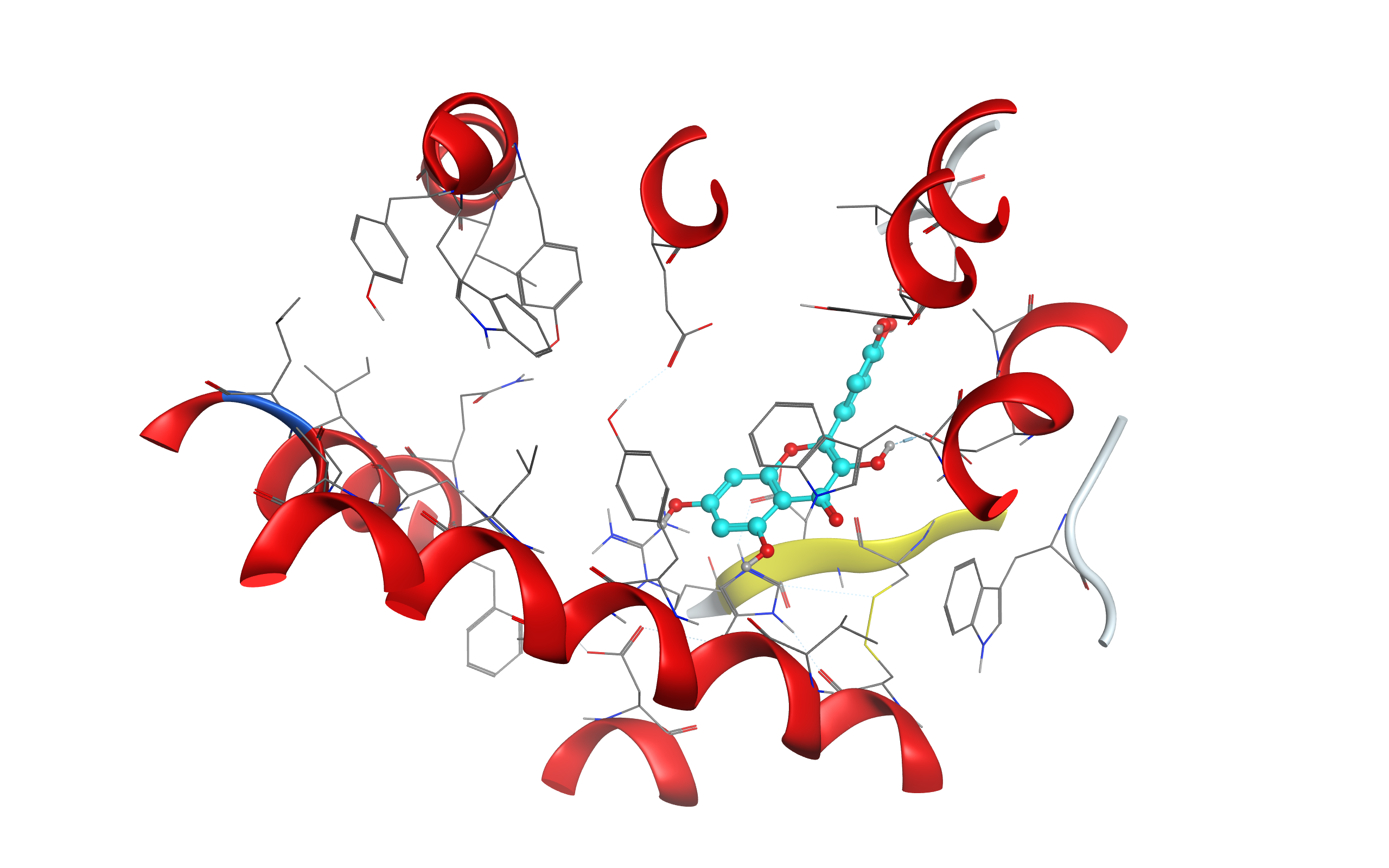 |
| **(1s,4s)-Eucalyptol** | 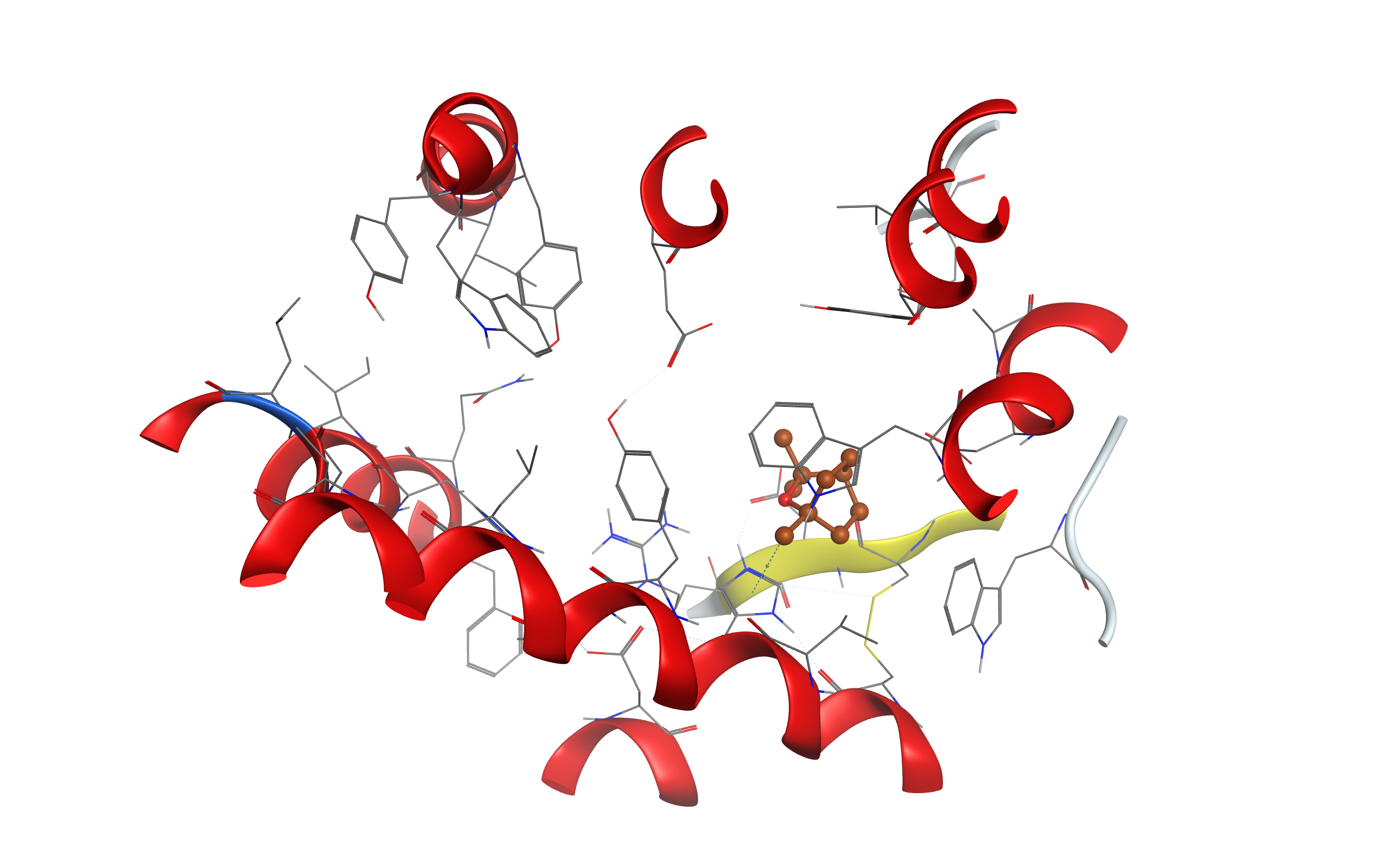 |

**Fig. S5** The docked conformation of (A) Curcumin, (B) Trans-resveratrol, (C) Quercetin, and (D) (1s,4s)-Eucalyptol at the inhibition binding site of receptor protein and superimposed structures of docked ligands with protein 3ODU of CXCR4 receptor

| 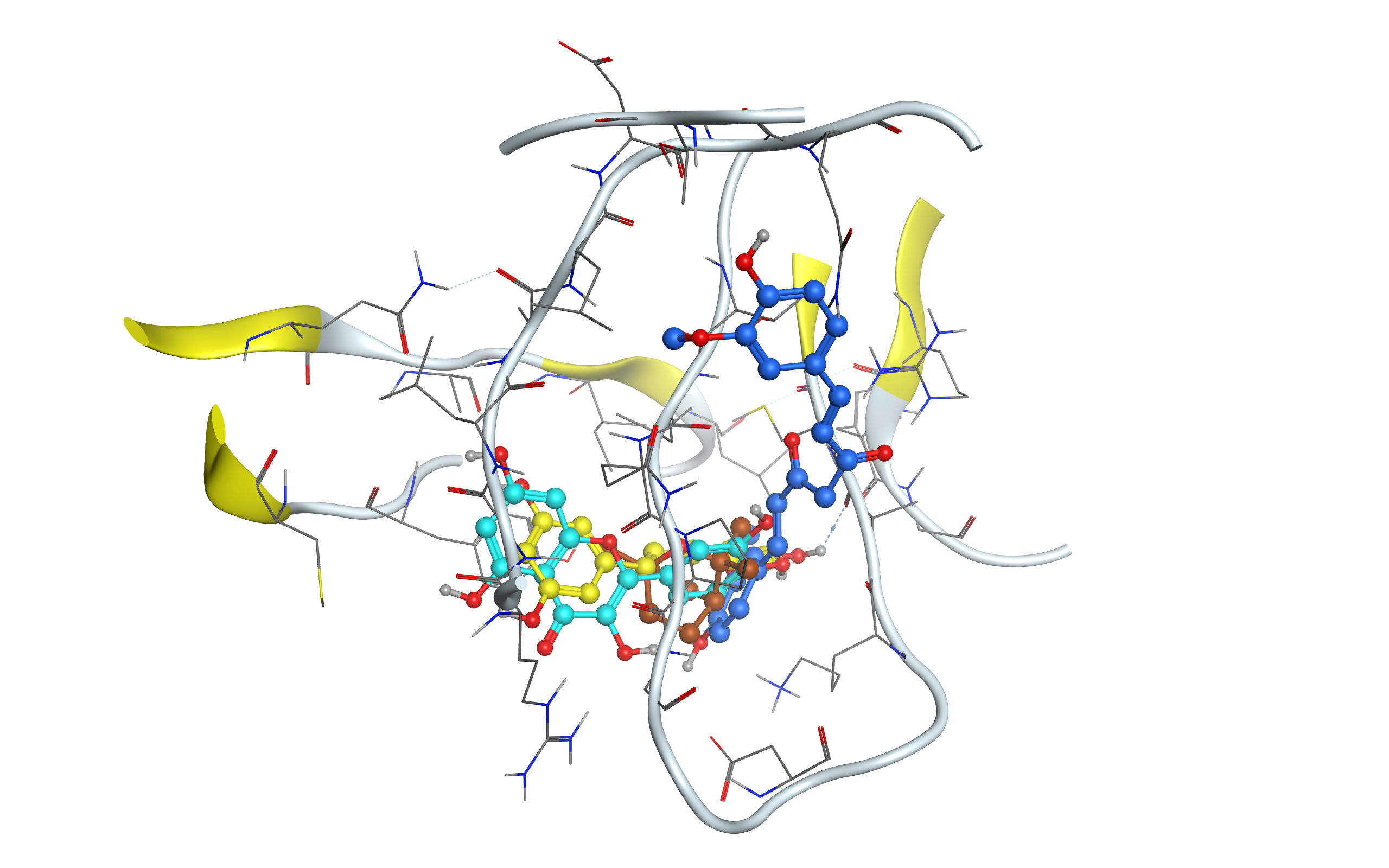 | |
| --- | --- |
| **Curcumin** | 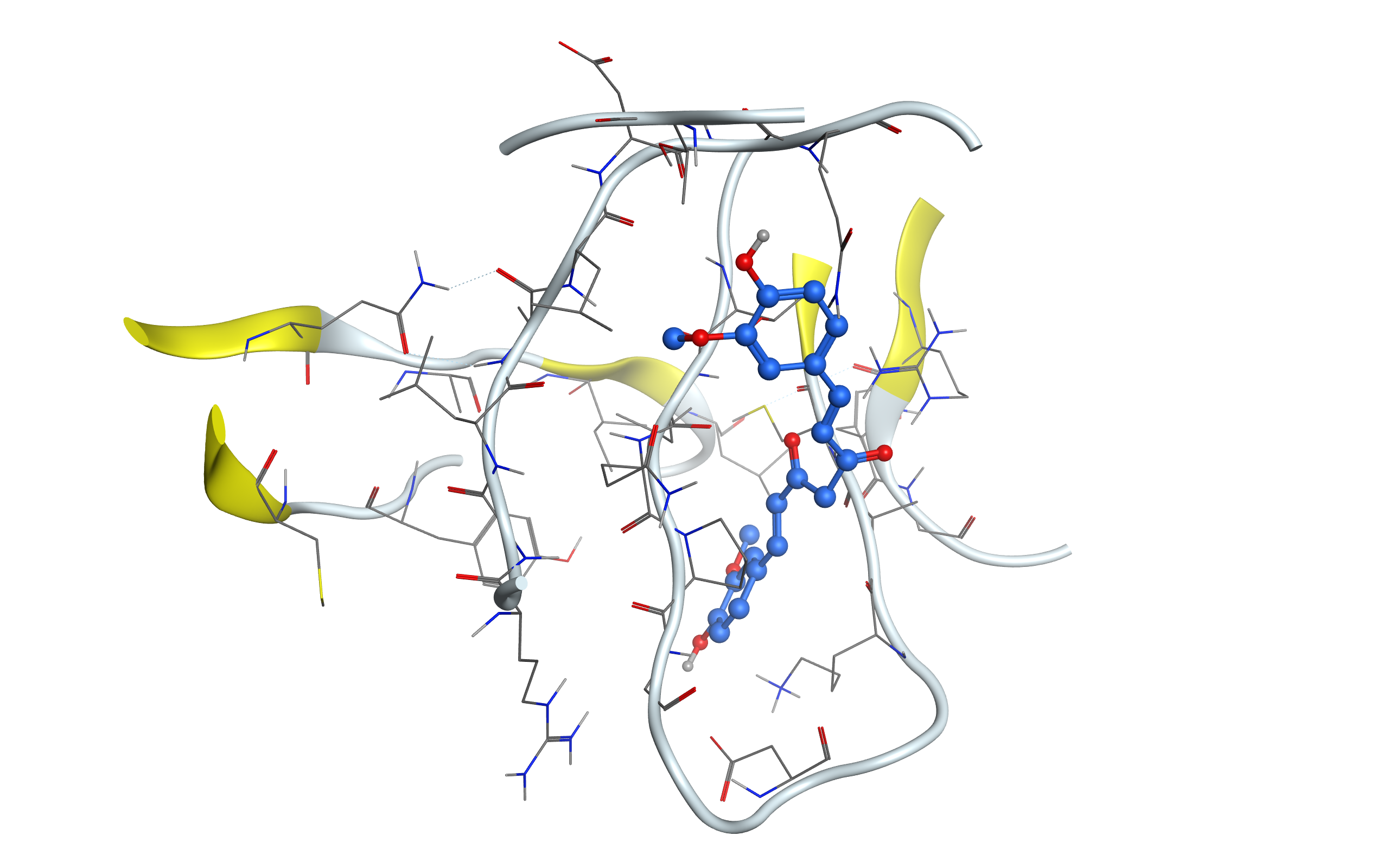 |
| **Trans-resveratrol** | 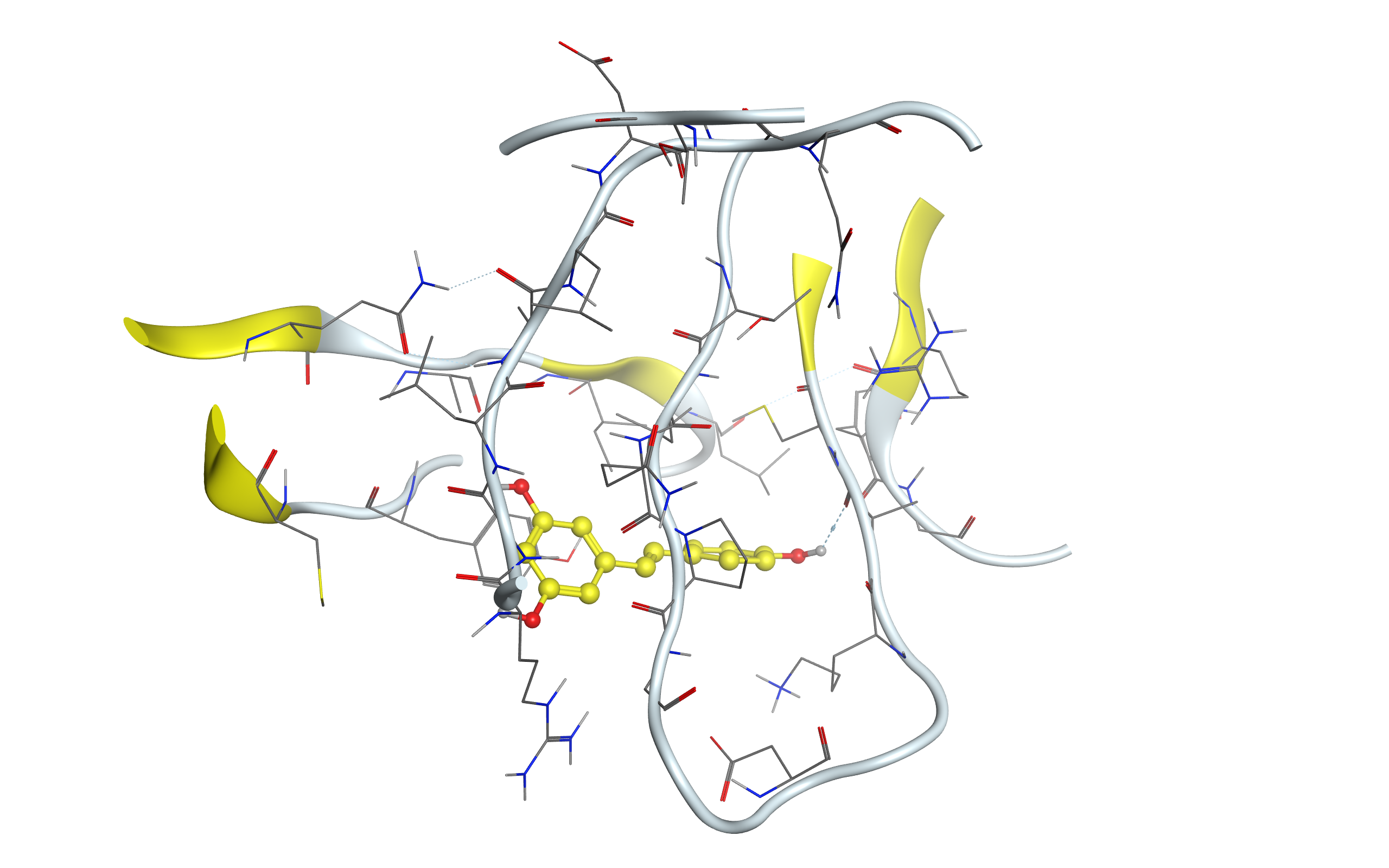 |
| **Quercetin** | 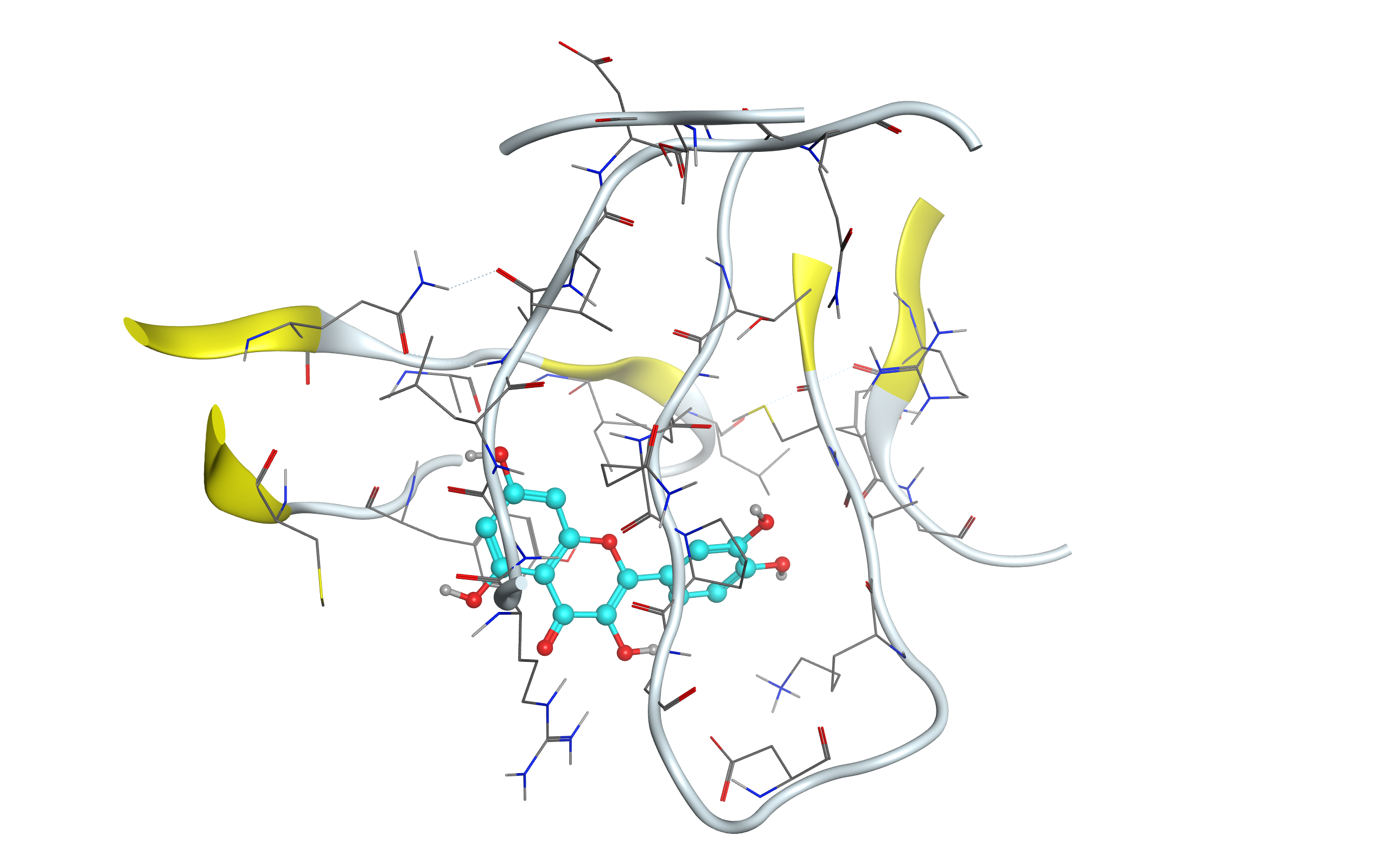 |
| **(1s,4s)-Eucalyptol** | 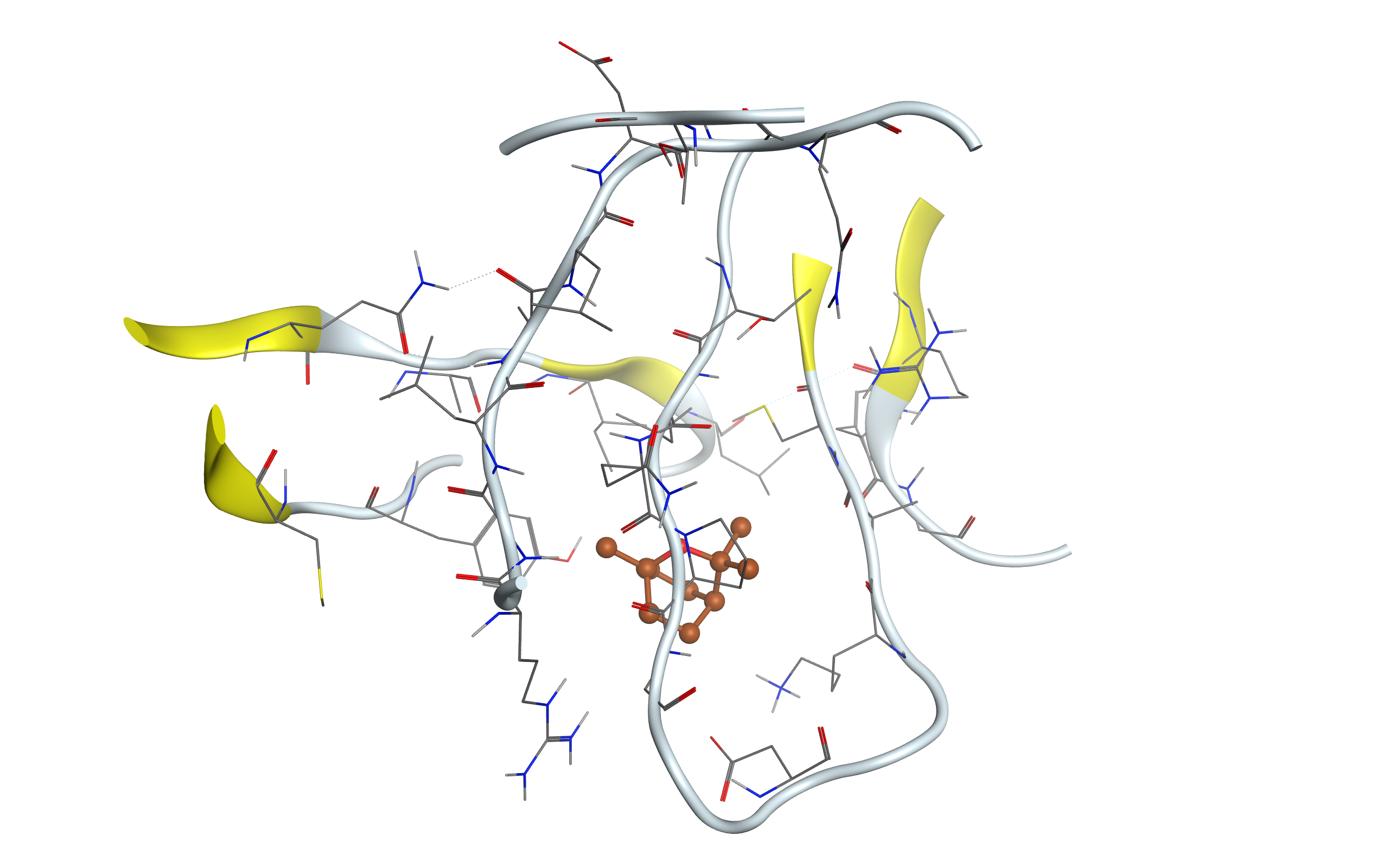 |

**Fig. S6** The docked conformation of (A) Curcumin, (B) Trans-resveratrol, (C) Quercetin, and (D) (1s,4s)-Eucalyptol at the inhibition binding site of receptor protein and superimposed structures of docked ligands with protein 6K3F of CXCR 7 receptor

| **Curcumin** | 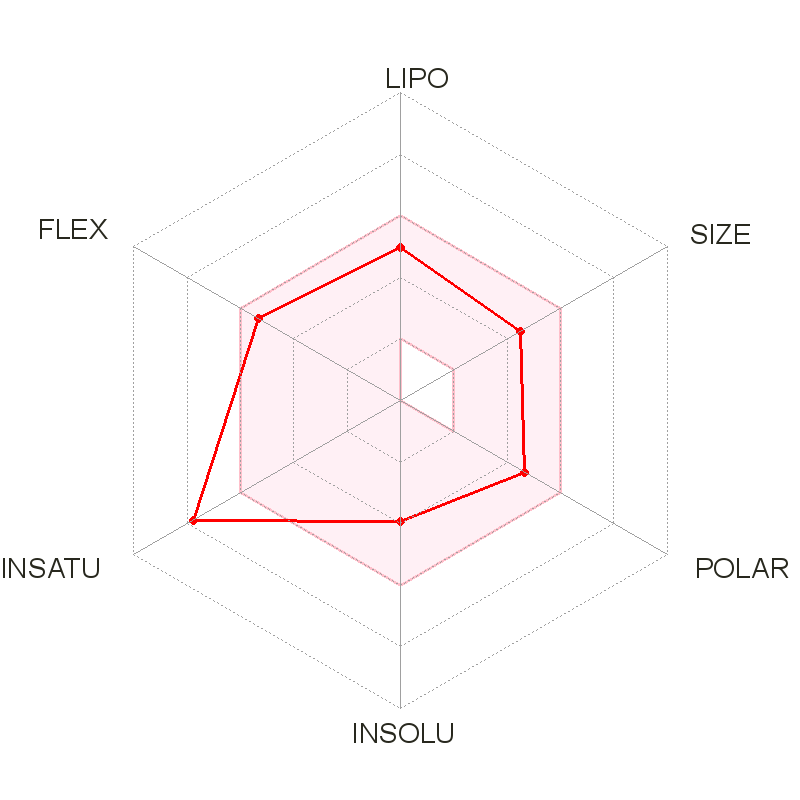 |
| --- | --- |
| **Trans-resveratrol** | 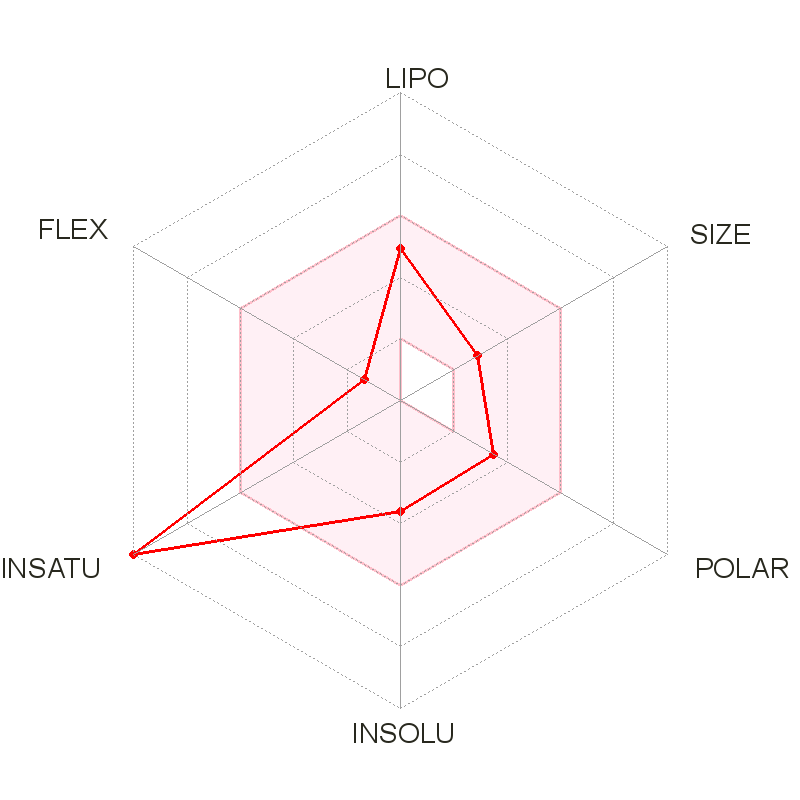 |
| **Quercetin** | 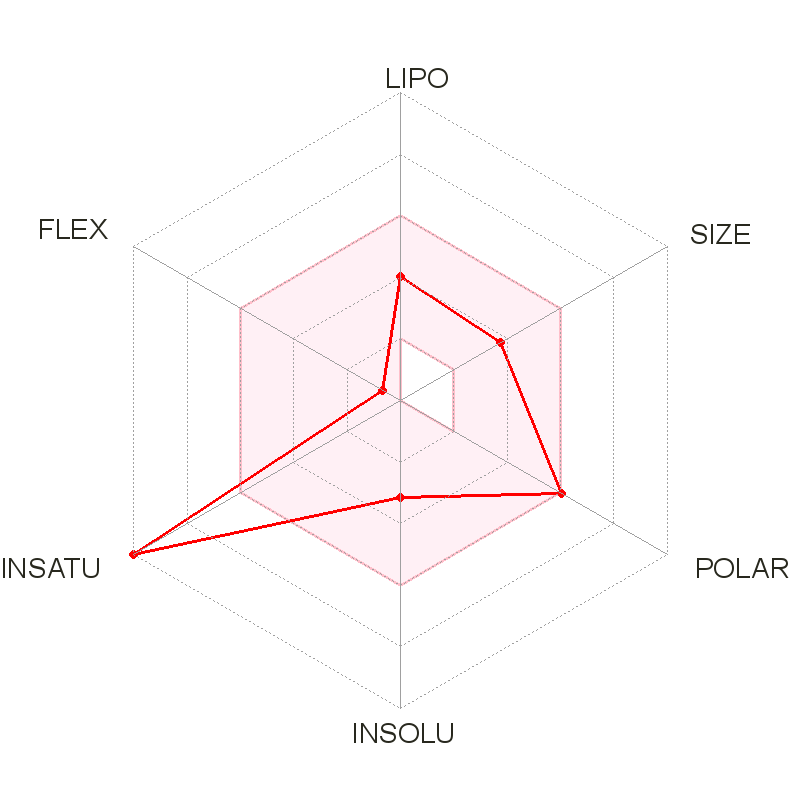 |
| **(1s,4s)-Eucalyptol** | 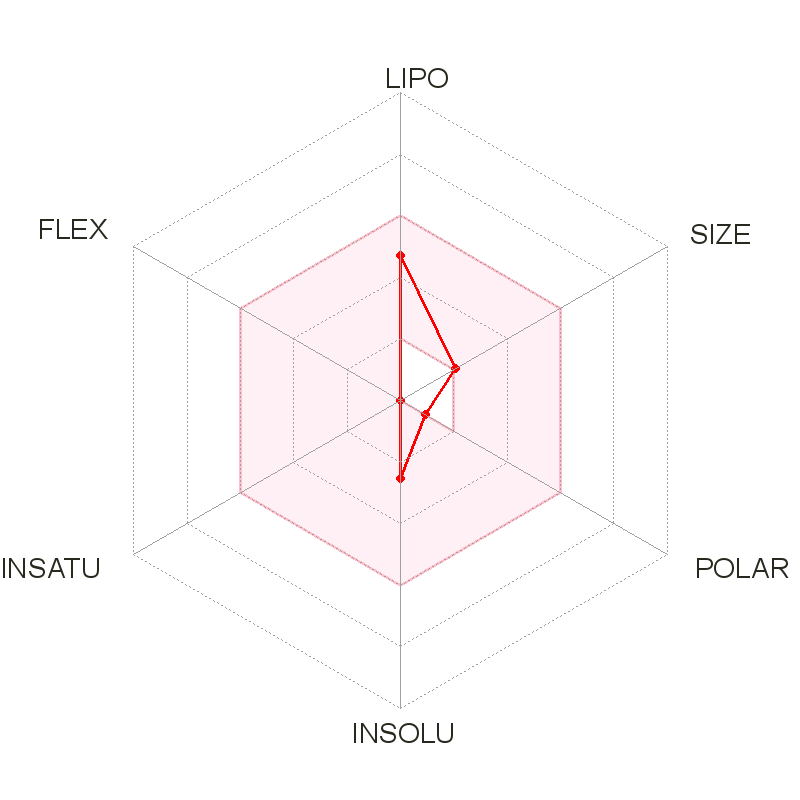 |

**Fig. S7** The bioavailability radar with the pink area. It represents the optimal range for physicochemical properties for oral bioavailability. Lipophilicity (-0.7 < XlogP3 <+0.5), size (150 < MW < 500 g/mol), polarity (20A^2^ <TPSA < 130A^2^), Insolubility (0 < Log S (ESOL) < 6), Insaturation (0.25 < Fraction Csp^3^ < 1), and flexibility (number of rotatable bonds < 9). The compounds, (A) Curcumin, (B) Trans-resveratrol, (C) Quercetin, and (D) (1s,4s)-Eucalyptol, are predicted orally bioavailable

| **Curcumin** | 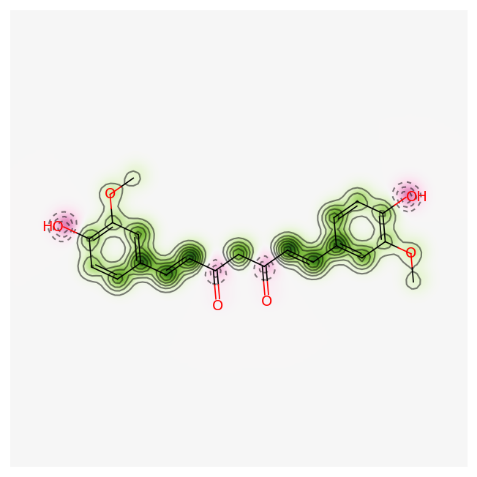 |
| --- | --- |
| **Trans-resveratrol** | 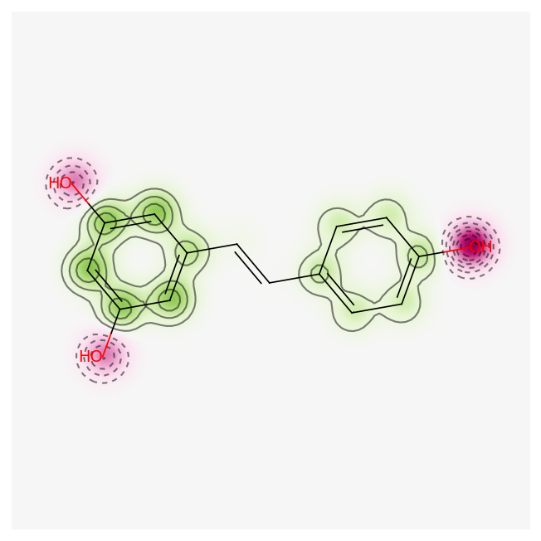 |
| **Quercetin** | 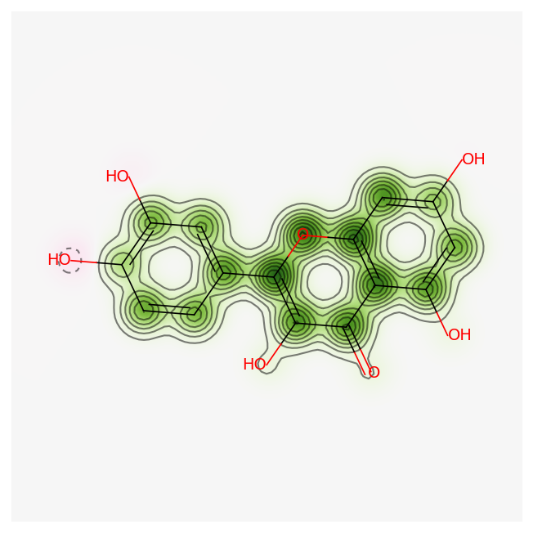 |
| **(1s,4s)-Eucalyptol** | 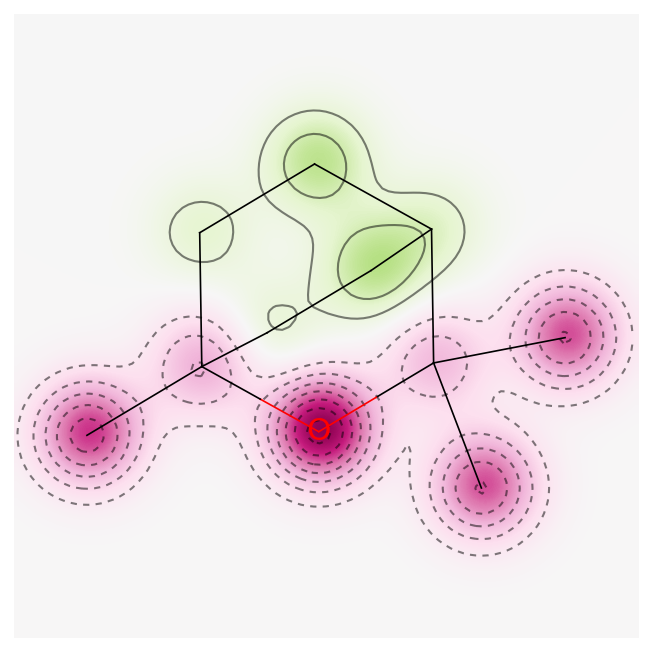 |

**Fig. S8** The predicted probability maps of (A) Curcumin, (B) Trans-resveratrol, (C) Quercetin, and (D) (1s,4s)-Eucalyptol. The more contour lines and intense green color means a higher positive contribution of an atom or a fragment to the hERG blockage, while pink means that it contributes to decrease of hERG blockage, and gray means no contribution

|  | **HOMO** | **LUMO** |
| --- | --- | --- |
| **Curcumin** | 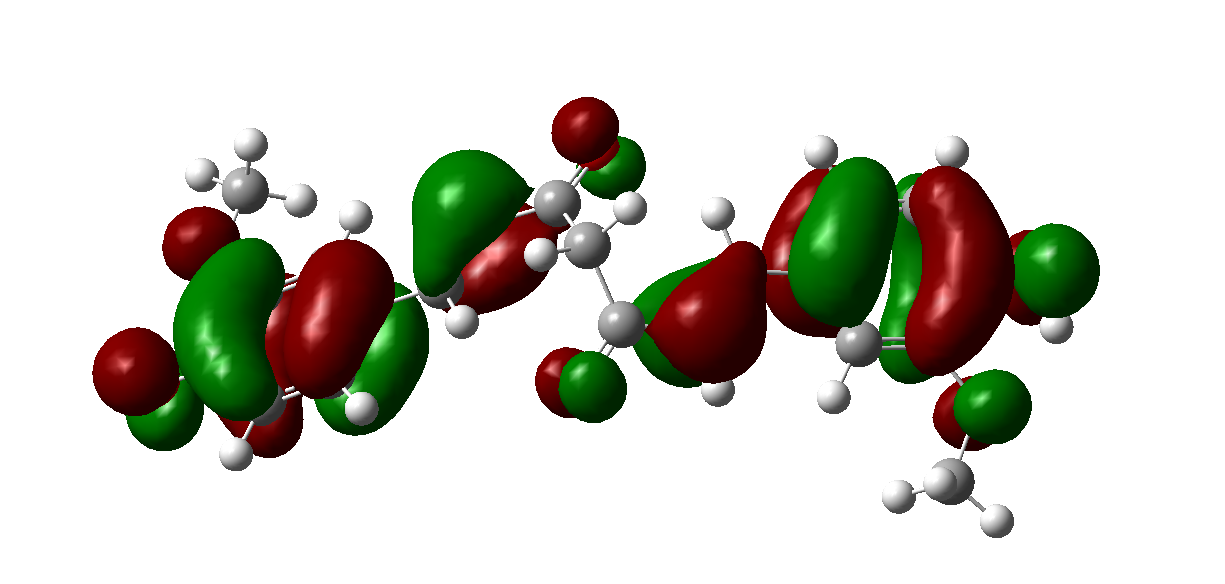 | 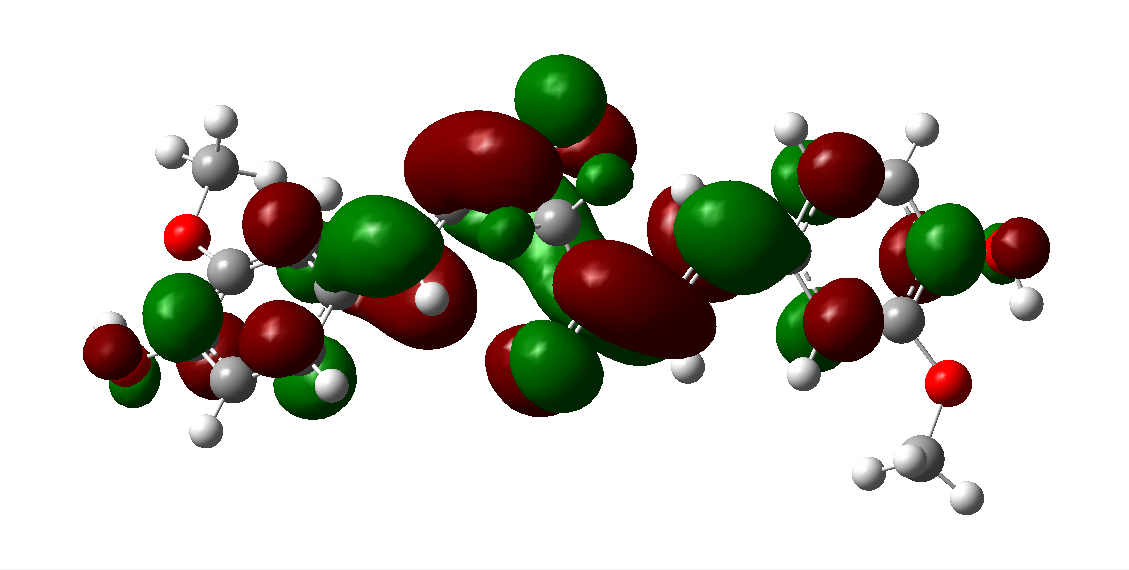 |
| **Trans-resveratrol** | 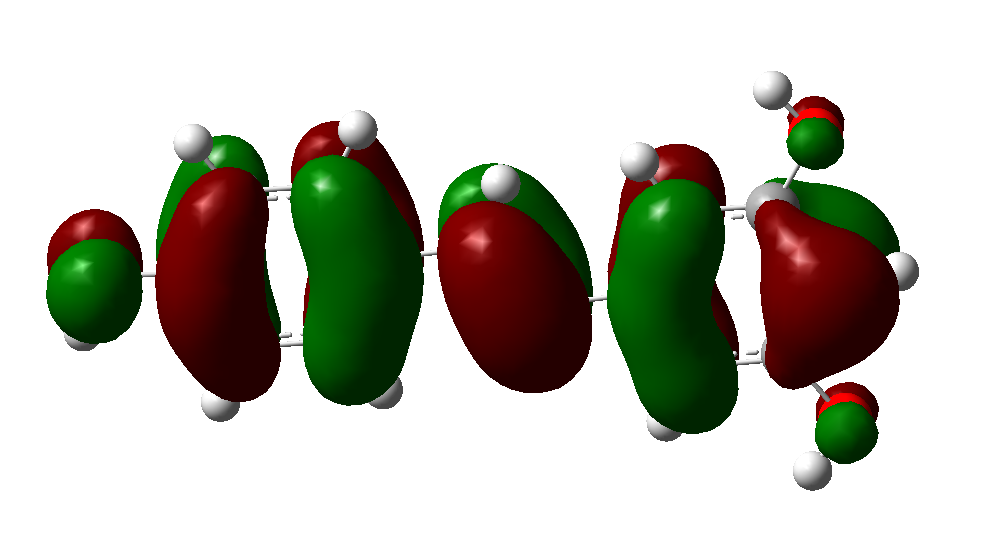 | 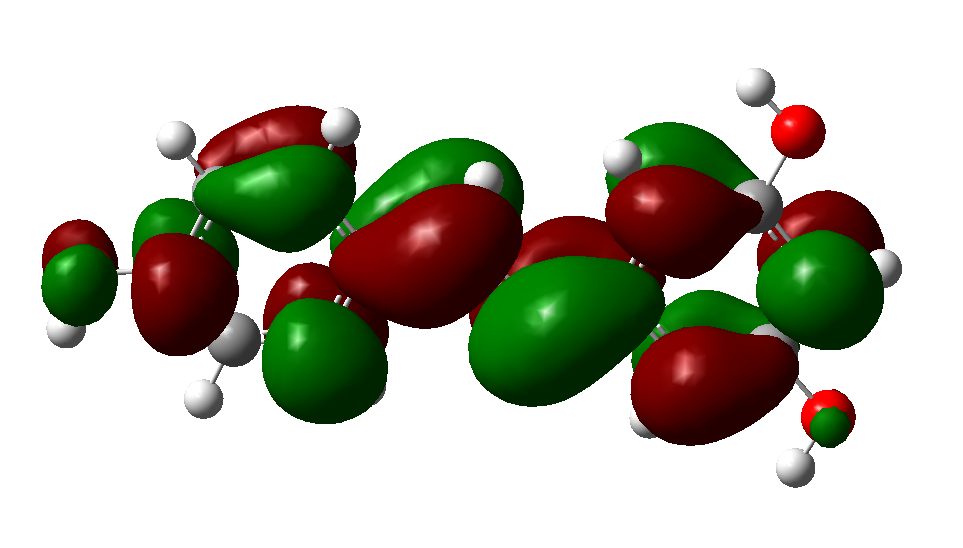 |
| **Quercetin** | 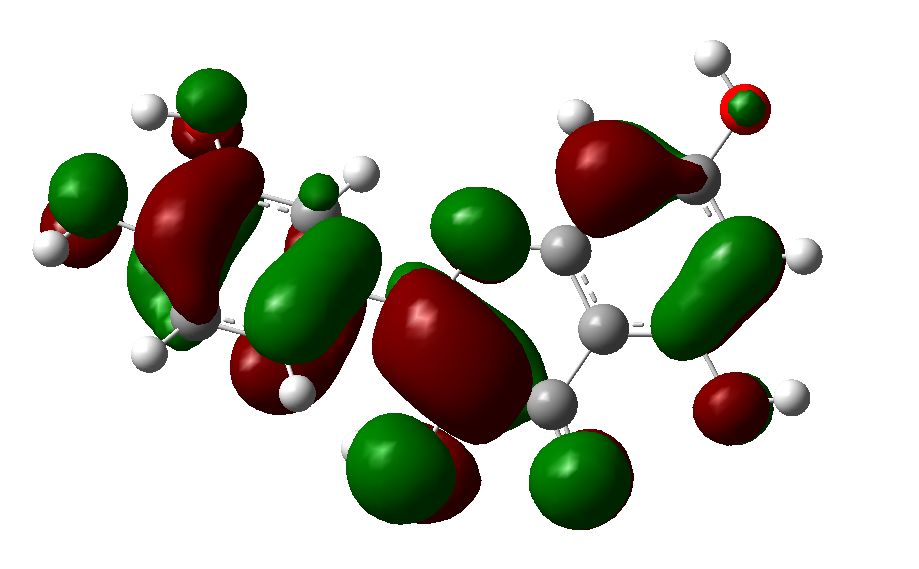 | 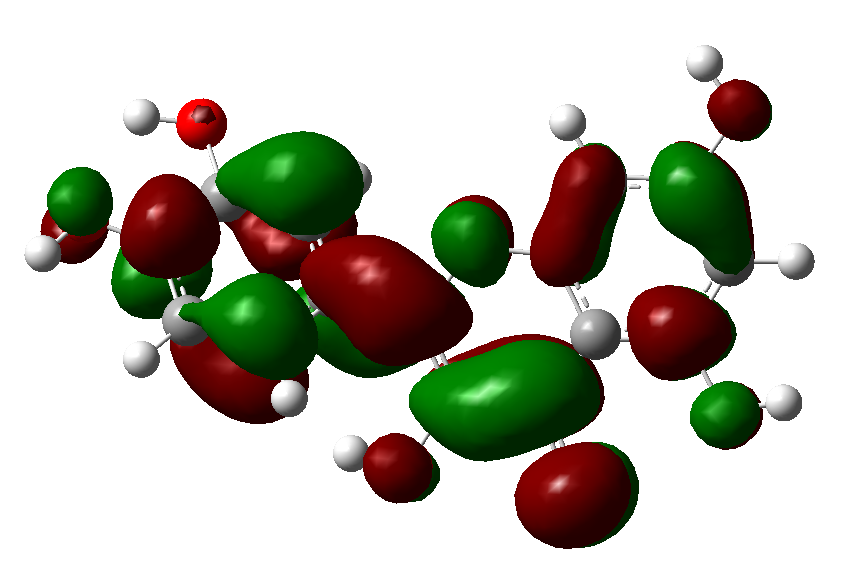 |
| **(1s,4s)-Eucalyptol** | 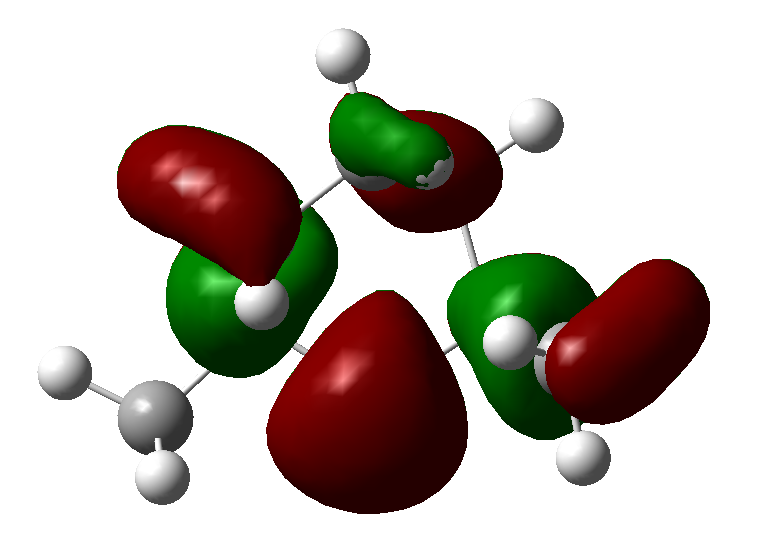 | 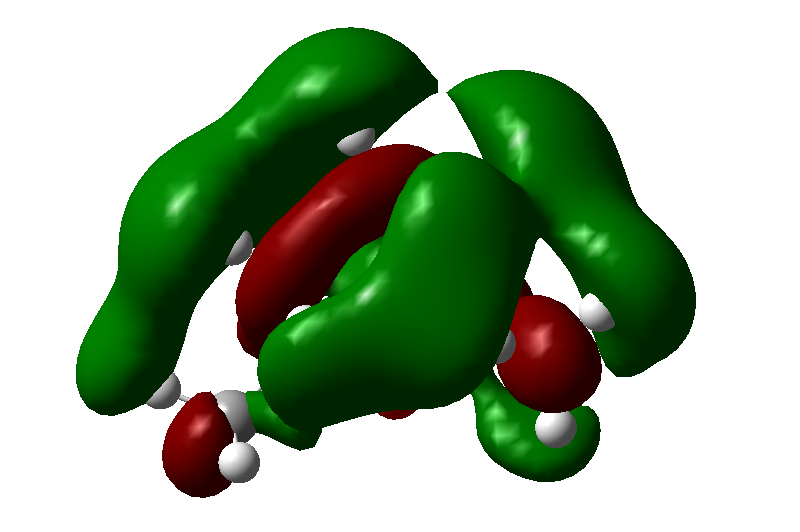 |

**Fig. S9** The 3D HOMO and LUMO frontier molecular orbital of (A) Curcumin, (B) Trans-resveratrol, (C) Quercetin, and (D) (1s,4s)-Eucalyptol.

**
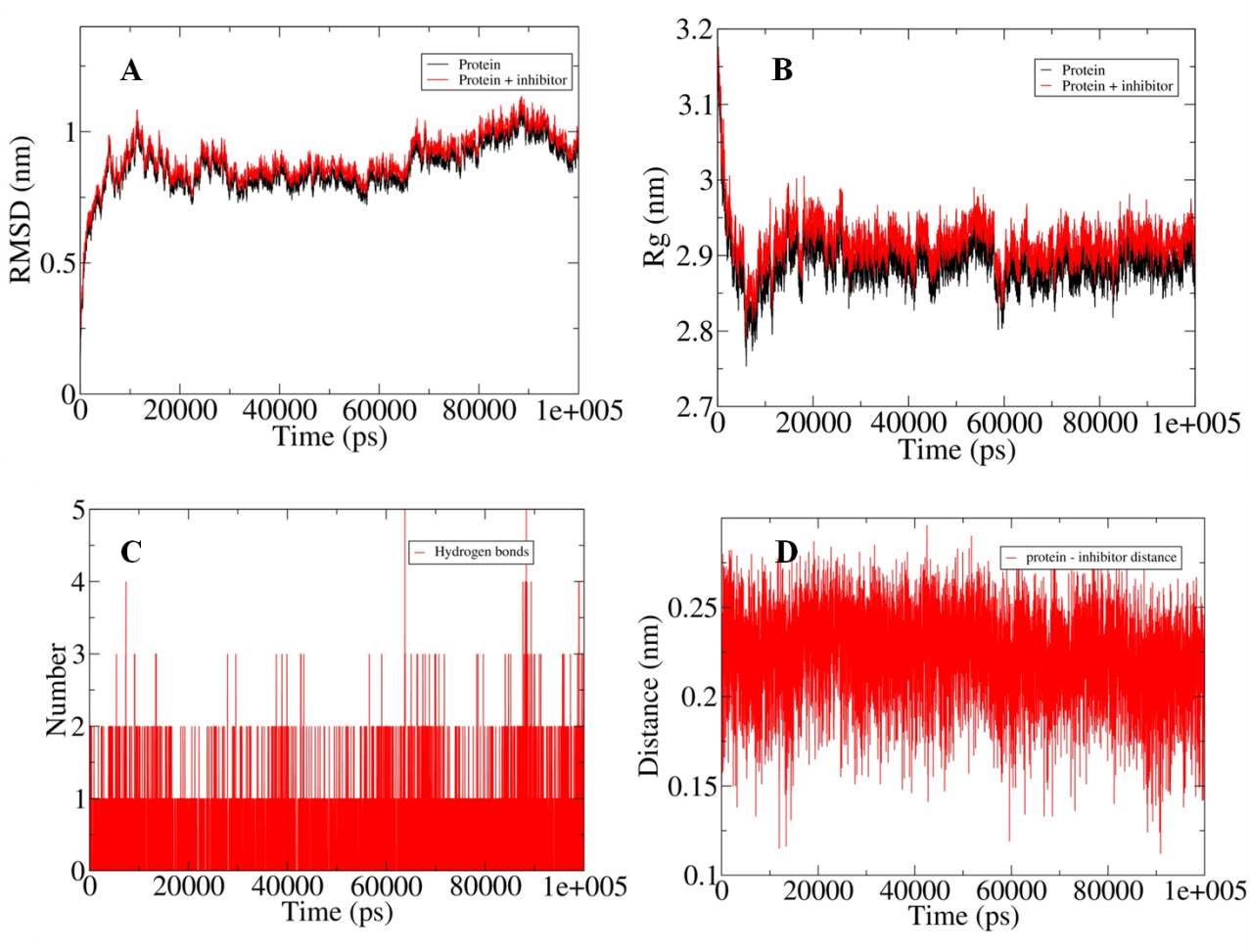
**

**Fig. S10** The results obtained from 100 ns molecular dynamics (MD) simulations with: (A) Changes in the root mean square deviation (RMSD) values for free protein and docked system, (B) Changes in radius of gyration (Rg), (C): Changes in the pattern of hydrogen binding, (D): Calculated distance between the protein and inhibitor
